# Regenerating forests around Taï National Park sustain biodiversity despite constrained floristic recovery

**DOI:** 10.1371/journal.pone.0356900

**Published:** 2026-10-21

**Authors:** Aya Diane Larissa Houphouët, Yao Charles Sangne, Abdoulaye Diarrassouba, Evans Ehouman, Bruno Hérault

**Affiliations:** 1 Université Jean Lorougnon Guédé, Unité de Formation et de Recherche en Environnement, Daloa, Côte d’ Ivoire; 2 Office Ivoirien des Parcs et Réserves, Soubré, Côte d’ Ivoire; 3 CIRAD, Forêts et Sociétés, Montpellier, France; 4 Forêts et Sociétés, Univ Montpellier, CIRAD, Montpellier, France; University of Mississippi, BRAZIL

## Abstract

Extensive deforestation and disturbance have reshaped forest landscapes in West Africa, including areas surrounding Taï National Park, the largest remaining block of Upper Guinean rainforest. Although many degraded areas are currently undergoing regeneration, the capacity of these secondary forests to recover their biodiversity attributes remains insufficiently assessed within this park. This study quantifies the multidimensional restoration of biodiversity across 125 secondary forest plots and 15 primary forest plots representing 14,731 inventoried individuals in four sectors of the park. Using a Bayesian modelling framework, we estimate recovery trajectories of Shannon diversity, floristic composition, functional traits (wood density, leaf mass per area, seed mass), and conservation-relevant species. The different biodiversity attributes recovered at contrasting rates. Diversity was restored more rapidly (λ = 0.06) than floristic composition (λ = 0.03), and these recovery rates varied according to environmental variables. Among these, the presence of remnant trees showed the highest median on diversity (0.53 ± 0.61) and floristic composition (0.37 ± 0.17), promoting the rapid recovery of both attributes, followed by prior land use, particularly cocoa farming which also positively influenced the recovery of alpha diversity (λ = 0.03) and floristic composition (λ = 0.02). Regarding functional traits, they displayed contrasting dynamics: leaf mass per area (LMA) and seed mass recovered rapidly along successional gradients, whereas wood density followed a more gradual recovery trajectory. From a conservation perspective, although the proportion of IUCN Red List species remained stable along the successional gradient, old-growth indicator species were significantly more abundant in primary forests. To ensure the long-term conservation of biodiversity, protecting both primary and regenerating forests is essential to preserve the ecological resilience of this park.

## Introduction

Tropical forests in West Africa harbour exceptionally high levels of biodiversity and form part of one of the world’s 25 biodiversity hotspots [1,2]. Despite their ecological importance, these forests have experienced intense deforestation in recent decades, resulting in substantial biodiversity loss [3]. In Côte d’Ivoire, forest conversion has been primarily driven by agricultural expansion, especially cocoa, coffee, and oil palm alongside logging, charcoal production, infrastructure development, and mining [3–5]. These transformations have led not only to biodiversity decline but also to the degradation of ecosystem services and climate regulation.

Against this backdrop of widespread forest loss, restoring degraded lands has emerged as an essential complementary strategy to the conservation of remaining old-growth forests [6]. Secondary forests now represent a substantial proportion of tropical forest cover [7–9] and may contribute meaningfully to biodiversity conservation [10,11]. Recovery is nonetheless complex: different biodiversity attributes do not recover at the same pace. Species diversity, for example, often increases relatively rapidly following agricultural abandonment [12,13], whereas floristic composition may take decades to converge toward old-growth conditions [14,15]. Moreover, recovery trajectories are strongly shaped by land-use intensity, soil degradation, remnant trees that enhance seed dispersal, and by the connectivity between neighboring landscapes [13,16–18].

Importantly, biodiversity recovery cannot be fully understood from taxonomic patterns alone. Beyond changes in species richness and composition, ecological succession entails shifts in species’ life-history strategies and functional attributes [19]. Early successional stages are typically dominated by fast-growing pioneer species, whereas late-successional and shade-tolerant species establish progressively as forest structure develops [14,20]. These successional dynamics are reflected in changes in functional traits, which provide mechanistic insight into ecosystem functioning and resilience [21]. Traits such as wood density, leaf mass per area (LMA), and seed mass are closely linked to growth–mortality trade-offs, resource-use strategies, and regeneration processes [22–25]. Seed mass, in particular, mediates the balance between colonization ability and establishment success [26–28]. Integrating functional composition with taxonomic and compositional analyses therefore provides a more comprehensive framework for evaluating forest recovery.

Old-growth forests are now extremely scarce in West Africa, a region where deforestation rates remain among the highest globally. As a result, conservation increasingly depends on the capacity of secondary forests to sustain biodiversity. Yet it remains unclear whether regenerating forests can meaningfully substitute for primary forests in maintaining endangered species, functional diversity, and ecosystem processes. Addressing this uncertainty is critical for guiding restoration strategies, landscape planning, and protected-area buffering. These issues are especially critical in landscapes subjected to intense anthropogenic pressure, such as those surrounding Taï National Park in south-western Côte d’Ivoire. Taï National Park constitutes the largest remaining continuous block of Upper Guinean humid rainforest and one of the last extensive remnants of tropical rainforest in West Africa. As a major stronghold of regional biodiversity, it harbours numerous endemic and threatened species, including the pygmy hippopotamus (Choeropsis liberiensis), the western chimpanzee (Pan troglodytes verus), and Diana's monkey (Cercopithecus diana), all classified as Endangered on the IUCN Red List. However, episodes of degradation particularly during the 2002–2011 politico-military crises have affected both the park and its periphery. Since 2015, degraded areas have been undergoing natural regeneration [29], creating a mosaic of intact old-growth forest, disturbed old-growth forest, and secondary forests of varying ages. In such a globally significant forest landscape, understanding the extent and pace of biodiversity recovery is essential for informing regional conservation and restoration strategies.

To date, few studies in Upper Guinean forests have jointly quantified taxonomic, functional, compositional, and conservation-relevant recovery using reference-based models. Here, we provide one of the first multidimensional assessments of natural forest regeneration surrounding Taï National Park, integrating community composition, functional traits, biomass, and species conservation status within a unified Bayesian framework. To provide conceptual clarity, we define “recovery” as the degree of convergence of biodiversity attributes in secondary forests toward reference conditions observed in intact old-growth forests. Recovery therefore refers not only to increases in species diversity over time, but also to compositional similarity and functional convergence relative to old-growth benchmarks. This reference-based approach allows us to distinguish between rapid gains in taxonomic diversity and slower or incomplete recovery of compositional and functional attributes. Building on this framework, we analyse biodiversity recovery trajectories in secondary forests surrounding Taï National Park. Specifically, we address the following questions: (i) What are the recovery trajectories of Shannon diversity and floristic composition, and which factors influence them? (ii) How do key functional traits such as wood density, leaf mass per area, and seed mass change along secondary succession, and can they serve as indicators of forest recovery? and (iii) Do secondary forests contribute to the persistence and recovery of conservation-relevant species?

## Methods

### Study area

The study was conducted in and around Taï National Park (TNP), located in south-western of Côte d’Ivoire between 5°15′ to 6°07′ N and 7°25′ to 7°54′ W (Fig 1). TNP constitutes the largest remaining continuous block of Upper Guinean humid rainforest and is recognised as a UNESCO World Heritage Site [30,31]. The park covers approximately 5,360 km² and represents one of the last extensive remnants of primary tropical rainforest in West Africa [32]. The region is characterised by a humid tropical climate with two rainy seasons (March to July and September to November) and two relatively drier periods (a short dry season in August and a longer one from December to February). Mean annual rainfall ranges between 1,700 and 2,200 mm, and mean annual temperature is approximately 25–27 °C. Soils are predominantly ferralitic and vary locally depending on topography and drainage conditions. Vegetation is dominated by evergreen and semi-deciduous moist forest. The park harbours high levels of plant and vertebrate diversity, including numerous endemic and threatened species characteristic of the Upper Guinean forest block [33,34]. Surrounding landscapes have been extensively transformed by agricultural expansion, particularly cocoa cultivation, as well as logging and artisanal gold mining.

**Fig 1:**
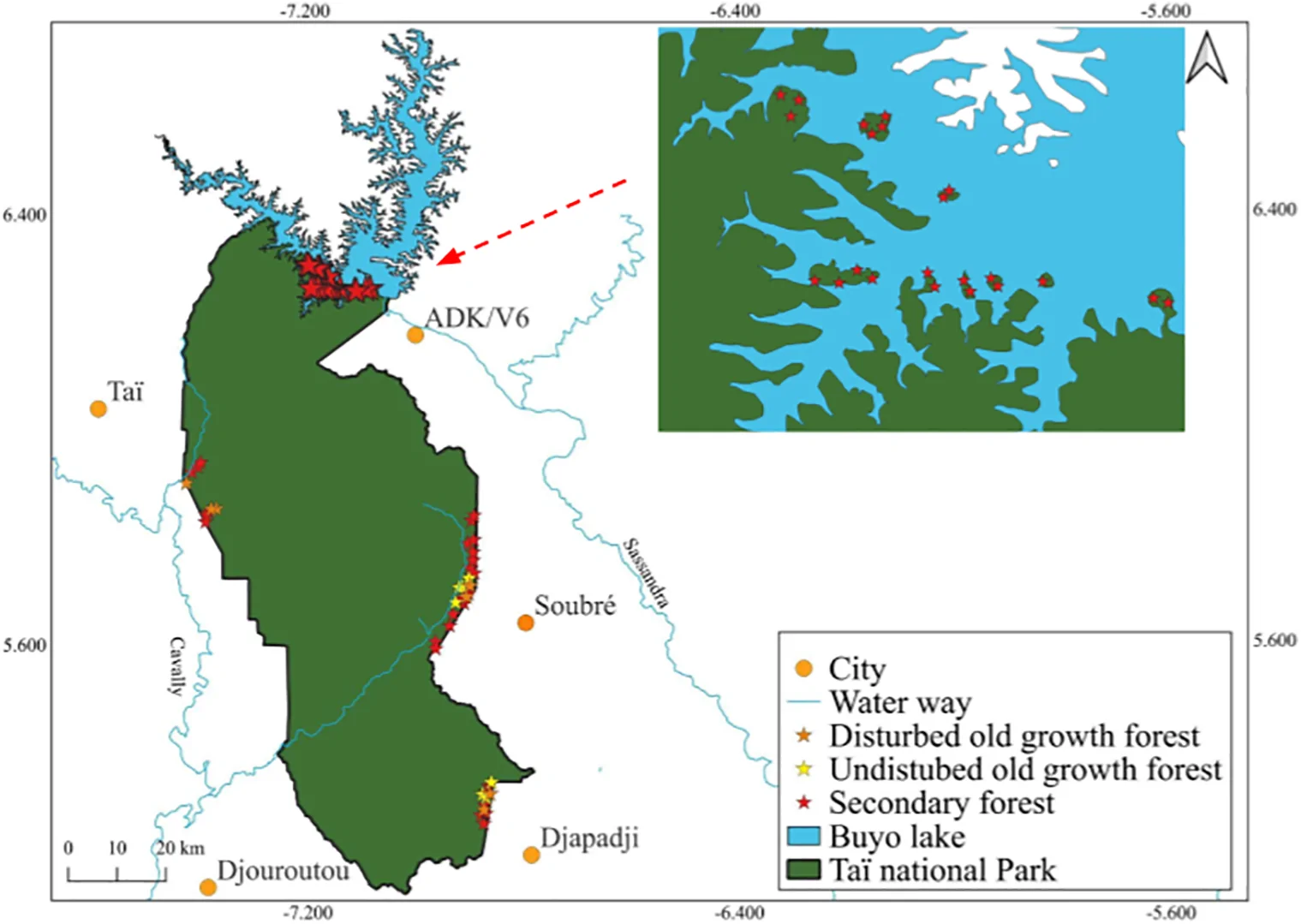
Spatial distribution of sampling plots across Taï National Park and surrounding landscapes in south-western Côte d’Ivoire. Sampling plots represent a chronosequence of secondary forests (9–26 years since abandonment; n = 125) and reference intact old-growth forests (n = 15). Plots are distributed within and around park boundaries. The map also shows Buyo Lake, major waterways, and nearby cities to provide geographic context. The inset highlights the northern sampling cluster near Buyo Lake, where forest fragmentation has created a mosaic of secondary and old-growth stands. Source: © OIPR.

During the post-electoral crises of 2000 and 2010, reduced surveillance led to forest encroachment and degradation in parts of the park and its periphery. Since 2015, restoration and natural regeneration processes have been ongoing in previously degraded areas [35]. As a result, the current landscape forms a mosaic of intact old-growth forest, disturbed old-growth forest, and secondary forests of varying ages originating from former agricultural lands or mining sites.

### Vegetation data

A total of 140 vegetation plots were established in and around Taï National Park. Of these, 125 plots were located in secondary forests and 15 plots in intact old-growth forests, which served as reference sites. Each plot measured 25 × 25 m (0.0625 ha). Secondary forests represented a chronosequence ranging from 9 to 26 years since abandonment. Forest age was determined based on information from local communities, park management records, and field evidence of past land use. Following the post-electoral crisis period, agricultural activities inside the park were discontinued and cocoa plantations were progressively removed as part of restoration and management interventions. Subsequent natural succession gave rise to stands of varying ages, forming the basis of the chronosequence analysed in this study. Within each 25 × 25 m plot, all woody stems with a diameter at breast height (DBH) ≥ 2.5 cm were inventoried. DBH was measured at 1.3 m above ground level. For multi-stemmed individuals, each stem meeting the diameter threshold was recorded separately. Lianas were excluded from the inventory. Species were identified in the field by experienced botanists. When identification was uncertain, specimens were examined using the regional floras of Aké-Assi [36] and Hutchinson & Dalziel [37] to ensure accurate determination. Although voucher specimens were not systematically deposited in a public herbarium, taxonomic verification procedures were applied to ensure consistency and reliability across plots. Nomenclature was standardised according to the Angiosperm Phylogeny Group IV (APG IV) classification system, and species names were harmonised to avoid synonymy or spelling inconsistencies within the dataset.

All original data used in this study are publicly available and can be accessed from Zenodo (https://doi.org/10.5281/zenodo.21505800).

### Functional trait data

We analysed three functional traits: wood density (WD), leaf mass per area (LMA), and seed mass. These traits were selected because of their established links to growth–mortality trade-offs, resource-use strategies, regeneration processes, and successional dynamics in tropical forests [22,23].

Wood density values were retrieved using the R package *BIOMASS* [38] through the function *getWoodDensity()*, which assigns species-level values when available and substitutes genus-level averages when species-specific data are missing. This procedure ensured complete coverage of wood density values across all species in the dataset. Seed mass and LMA values were obtained primarily from the TRY global plant trait database [39], including regional datasets integrated into TRY [40]. Seed mass data were additionally compiled from the SER-SID database (ser-sid.org). For species lacking values in these databases, seed mass measurements were supplemented using published data from [41].

When multiple trait values were available for a given species across datasets, the median value was used to reduce the influence of extreme observations. Species-level trait values were then assigned to all corresponding individuals in the vegetation dataset. Trait coverage varied among traits. Wood density values were available for 100% of species, seed mass for approximately 49% of individuals, and LMA for 56% of individuals.

### Environmental variables

To account for ecological and anthropogenic drivers of recovery, we recorded a set of environmental and disturbance-related variables expected to influence regeneration processes (Table 1).

**Table 1:** Plot-level environmental variables used to assess biotic legacies, edaphic constraints, landscape isolation, and ongoing anthropogenic activities potentially influencing biodiversity recovery in secondary forests surrounding Taï National Park, Côte d’Ivoire.

Variables	Description	Values
Animal	Number of large/medium-sized mammal species detected through indirect signs	0-3
Hydromorphy	Presence of hydromorphic soil features in surface horizons	0/1
Island	Plot located in isolated island	0/1
Remnants	Density of isolated old-growth trees persisting after land conversion	trees.ha^-1^
Activities	Number of disturbance types observed (poaching, agriculture,mining)	0-3

- Large and medium-sized mammals were included because they play a key role in seed dispersal and recruitment dynamics in tropical forests. Their presence can enhance colonization by late-successional species and influence community composition. Mammal occurrence was assessed through indirect field signs of species known to function as seed dispersers or large herbivores.
- Remnant trees were quantified as indicators of biological legacies from pre-disturbance forests. Such trees can provide local seed sources, structural complexity, and perching sites for frugivores, thereby potentially accelerating succession.
- Soil hydromorphy was recorded as a proxy for edaphic constraints on species establishment and growth, as waterlogged conditions can limit recruitment of certain functional groups.
- Island configuration was included to capture landscape isolation effects, which may reduce propagule influx and alter recovery trajectories.
- Signs of ongoing human activities were documented to account for continued disturbance pressure that may slow or redirect successional dynamics.

Together, these variables capture biotic, abiotic, and anthropogenic drivers of succession and were incorporated as covariates in subsequent models of biodiversity recovery.

### Conservation-relevant species

To evaluate the contribution of secondary forests to biodiversity conservation, we considered two categories of conservation-relevant species: (i) old-growth indicator species and (ii) species listed under threatened categories of the IUCN Red List.

Indicator species characteristic of old-growth forests were identified using the method of [42], implemented in the R package *indicspecies*. We compared two groups of plots: intact old-growth forests and secondary forests younger than 20 years. The indicator value (IndVal) combines measures of specificity and fidelity to a given habitat type, allowing the identification of species strongly associated with old-growth conditions. Species were considered significant old-growth indicators when their indicator value was associated with a permutation test P-value < 0.002. A stringent significance threshold was applied to reduce the risk of false positives and to ensure robust identification of species reliably associated with old-growth forest conditions. For each plot, we subsequently quantified the occurrence and abundance of these indicator species.

For all inventoried species, conservation status was assessed using the IUCN Red List of Threatened Species (https://www.iucnredlist.org/). Species classified as Vulnerable (VU) or Endangered (EN) were considered conservation-relevant for this analysis. The presence and abundance of these species were quantified across forest types to evaluate their persistence and potential recovery during secondary succession.

### Biodiversity metrics

Diversity was quantified using Shannon diversity, expressed as the Hill number of order 1 (q = 1). Diversity of order 1 corresponds to the exponential of Shannon entropy and represents the effective number of species in a community. Estimates were computed using the R package *entropart* [43]. Sample coverage was first estimated for each plot using the function *coverage()*. As coverage was consistently above 0.90 across plots, diversity was estimated at a standardized coverage level of 0.90 to ensure comparability among plots. This coverage-based standardization minimizes bias due to incomplete sampling while avoiding distortions caused by unequal sampling effort.

Composition reflects both species identity and their relative abundances within each plot. To quantify compositional similarity between secondary forests (SF) and intact old-growth forests (OGF), we calculated the abundance-based Chao–Jaccard similarity index for all possible SF–OGF plot pairs. This index accounts for unseen shared species and corrects for undersampling, thereby providing a robust estimate of compositional similarity. To define the reference state, similarities among old-growth plots (OGF–OGF) were also calculated. These within-OGF similarities represent the natural variability of intact forests and define the benchmark toward which secondary forests are expected to converge during succession [15]. Although the 15 reference plots are modest relative to the park's total area, they were distributed across four sectors of the park to capture part of its spatial heterogeneity, and their pairwise similarity was used to explicitly characterise — rather than assume — the natural variability of old-growth forest composition used as the recovery benchmark.

### Modeling strategies

All models were built within a Bayesian framework. A Bayesian approach was chosen because of its flexibility in handling complex hierarchical structures, its ability to accommodate different likelihood distributions across response variables, and its explicit quantification of uncertainty through posterior distributions. A log-normal likelihood was used because all response variables considered (diversity, composition, and functional traits) are strictly defined on the positive real line (ℝ⁺) and exhibited right-skewed distributions [44]. Log-normal likelihoods were implemented in Stan using its standard parameterization, with mean parameters defined on the original scale and passed to the distribution through the log link. Models were implemented in Stan and fitted using Hamiltonian Monte Carlo (HMC) sampling for parameter estimation [45].

### Q1- biodiversity recovery trajectories

To estimate recovery trajectories and intrinsic recovery rates, we applied the exponential recovery model developed by [46]. This model assumes that the instantaneous recovery rate of an ecosystem attribute is proportional to the distance between its current state and its asymptotic value [44]. Such a formulation captures saturating recovery dynamics commonly observed during secondary succession.

For secondary forests, the likelihood model for a biodiversity attribute *X* (diversity or composition) in plot *p* at time *t* since abandonment was specified as:


$$\begin{array}{c} X_{p,t}\sim LN\left(\theta_{\text{0}}^{X}+\left(\;\theta_{+\infty }^{X}-\theta_{\text{0}}^{X}\right).\left(\text{1}-e^{{-\lambda }^{X}t_{p}}\right),\sigma_{X}^{\text{2}}\right) \end{array}$$ (1)

where:

- $X_{p,t}$ is the observed value of attribute *X* in plot *p* at time *t*,
- $\theta_{\text{0}}^{X}$ represents the initial value of *X* at the beginning of the recovery process,
- $\theta_{+\infty }^{X}$ is the asymptotic value of *X*, corresponding to the long-term equilibrium conditions,
- $\lambda^{X}$ is the intrinsic recovery rate controlling the speed of convergence toward the asymptote,
- $\sigma_{X}^{\text{2}}$ represents the residual variance among secondary forest plots.

For intact old-growth forests, time since regeneration is not defined and attribute values are assumed to represent the asymptotic state. The likelihood was therefore specified as:


$$\begin{array}{c} X_{p,t}\sim LN\left(\theta_{+\infty }^{X},\sigma_{X}^{\text{2}}\right) \end{array}$$ (2)

This formulation allows old-growth forests to directly inform the asymptotic parameter $\theta_{+\infty }^{X}$.

To account for anthropogenic disturbance effects in old-growth forests, asymptotic values were adjusted by including a disturbance effect term:


$$\begin{array}{c} X_{p,t}\sim LN\left(\theta_{+\infty }^{X}+\theta_{dist}^{X},\sigma_{X}^{\text{2}}\right) \end{array}$$ (3)

where $\theta_{dist}^{X}$ represents the effect of disturbance intensity on the asymptotic value of attribute *X*.

To evaluate how environmental and disturbance-related variables influenced recovery dynamics, covariates (Table 1) were incorporated into the recovery rate parameter $\lambda_{p}^{X}$. Because recovery rates are strictly positive, we modelled them using an exponential link function:


$$\begin{array}{c} {\;\lambda }_{p}^{X}=\lambda_{land}^{X}\times e^{\left(\sum_{j=\text{1}}^{J}\beta_{COVj}^{X}.{COV}_{j,p}\right)} \end{array}$$ (4)

where:

- $\lambda_{land}^{X}$ is the baseline recovery rate associated with previous land use,
- $\beta_{COVj}^{X}$ represents the effect of covariate *j* on recovery rate,
- ${COV}_{j,p}$ is the standardized value of covariate *j* for plot *p*.

All continuous covariates were standardized prior to analysis to facilitate parameter interpretation and comparison of effect sizes.

### Q2 - Functional trait dynamics

To analyse how functional traits change during succession, we used an individual-level Bayesian model linking trait values to both stem diameter *DBH* and forest age *t*. This approach captures shifts in trait distributions along the tree size gradient during recovery, consistent with the idea that secondary forests progressively approach old-growth trait–size relationships as forests mature [46]. Because trait values were strictly positive and right-skewed, they were modelled using a log-normal likelihood. For each tree *n* in secondary forests, trait values *Y* (wood density, leaf mass per area, or seed mass) were modelled as:


$$\begin{array}{c} Y_{n,p}\sim LN\left(\left(\alpha_{Y}+\beta_{Y}{.DBH}_{n}\right).\left(\text{1}-e^{{-\lambda }^{Y}t_{p}}\right),\sigma_{y}^{\text{2}}\right) \end{array}$$ (5)

where ${DBH}_{n}$ is the diameter of individual *n*, $t_{p}$ is time since abandonment for the plot *p* where the individual occurs, $\alpha_{Y}$ is the baseline value of the trait when the diameter effect is null, $\beta_{Y}$ quantifies the effect of DBH on the expected trait value, $\lambda^{Y}$ is the convergence rate toward the asymptotic state, and $\sigma_{y}^{\text{2}}$ is the residual variance. Under this formulation, the expected trait–size relationship converges toward the old-growth reference function $\left(\alpha_{Y}+\beta_{Y}{.DBH}_{n}\right)$ as forest age increases, thereby allowing trait distributions to progressively align with old-growth conditions along the DBH gradient.

For intact old-growth forests, trait values were assumed to reflect the asymptotic (reference) trait–size relationship:


$$\begin{array}{c} Y_{n}\sim LN\left(\left(\alpha_{Y}+\beta_{Y}{.DBH}_{n}\right),\sigma_{y}^{\text{2}}\right) \end{array}$$ (6)

To account for possible anthropogenic disturbance shifts away from intact reference conditions, we included an additive disturbance effect on the expected trait–size relationship:


$$\begin{array}{c} Y_{n}\sim LN\left(\theta_{Y}.\left(\alpha_{Y}+\beta_{Y}{.DBH}_{n}\right),\sigma_{y}^{\text{2}}\right) \end{array}$$ (7)

where $\theta_{Y}$ represents the effect of disturbance intensity on the expected value of trait *Y* (i.e., $\theta_{Y}$  > 1 indicates higher expected trait values under disturbance, whereas $\theta_{Y}$  < 1 indicates lower expected trait values).

**Q3 - Conservation-relevant species**

To assess whether conservation-relevant species are maintained or recovering during succession, we calculated, for each plot, the proportion of individuals belonging to (i) old-growth indicator species and (ii) IUCN-listed species (Vulnerable or Endangered). Secondary forests were grouped into two age classes (≤ 11 years and > 11 years since abandonment) and compared with intact old-growth forests. This comparison allowed us to evaluate whether the relative representation of conservation-relevant species increases with forest age and approaches old-growth conditions.

## Results

Across all forest types, we recorded 14,731 woody individuals (DBH ≥ 2.5 cm), corresponding to 174 species distributed among 138 genera and 48 families.

### Biodiversity recovery trajectories

Shannon diversity and floristic composition did not recover at the same rate (Fig 2, Table 2). Shannon diversity increased rapidly during succession, reaching approximately 75% of its asymptotic value within the first 40 years. The intrinsic recovery rate was λ = 0.06 (90% CI: 0.02–0.17). Diversity started at an estimated 8.94 effective species (θ₀) and converged toward an asymptotic value of 20.39 in old-growth forests (θ₊∞). In contrast, floristic composition recovered more slowly (λ = 0.03; 90% CI: 0.00–0.16). Because λ is constrained to be strictly positive by the model's exponential link function, this interval does not literally include zero; however, its lower bound is very close to zero, indicating that a near-negligible, practically stagnant recovery rate cannot be excluded. On average, secondary forests required over 40 years to approach half of the asymptotic similarity observed in old-growth forests (Fig 2). Composition increased from an initial value of 0.19 to an asymptote of 0.72 (Table 2). Previous land use influenced recovery rates. Recovery was faster in former cocoa farms than in former mining sites for diversity, whereas land-use effects on compositional recovery were negligible (Table 2).

**Table 2:** Posterior summaries of the recovery model parameters for diversity and composition in secondary forests of West Africa.

Parameters	Diversity			Composition		
	Median	5th	95th	Median	5th	95th
$\theta_{\text{0}}$	8.94	3.83	17.96	0.19	0.02	0.71
$\theta_{+\infty }$	20.39	16.15	24.90	0.72	0.52	0.97
λ	0.06	0.02	0.17	0.03	0.00	0.16
$\lambda_{Cocoa}$	0.03	0.01	0.08	0.01	0.00	0.03
$\lambda_{Mining}$	0.02	0.01	0.06	0.01	0.00	0.02
$\lambda_{others}$	0.08	0.02	0.18	0.02	0.00	0.06
$\theta_{dist}$	0.51	−3.88	4.44	−0.27	−0.44	−0.17
σ	0.44	0.40	0.50	1.74	1.68	1.81

**Fig 2:**
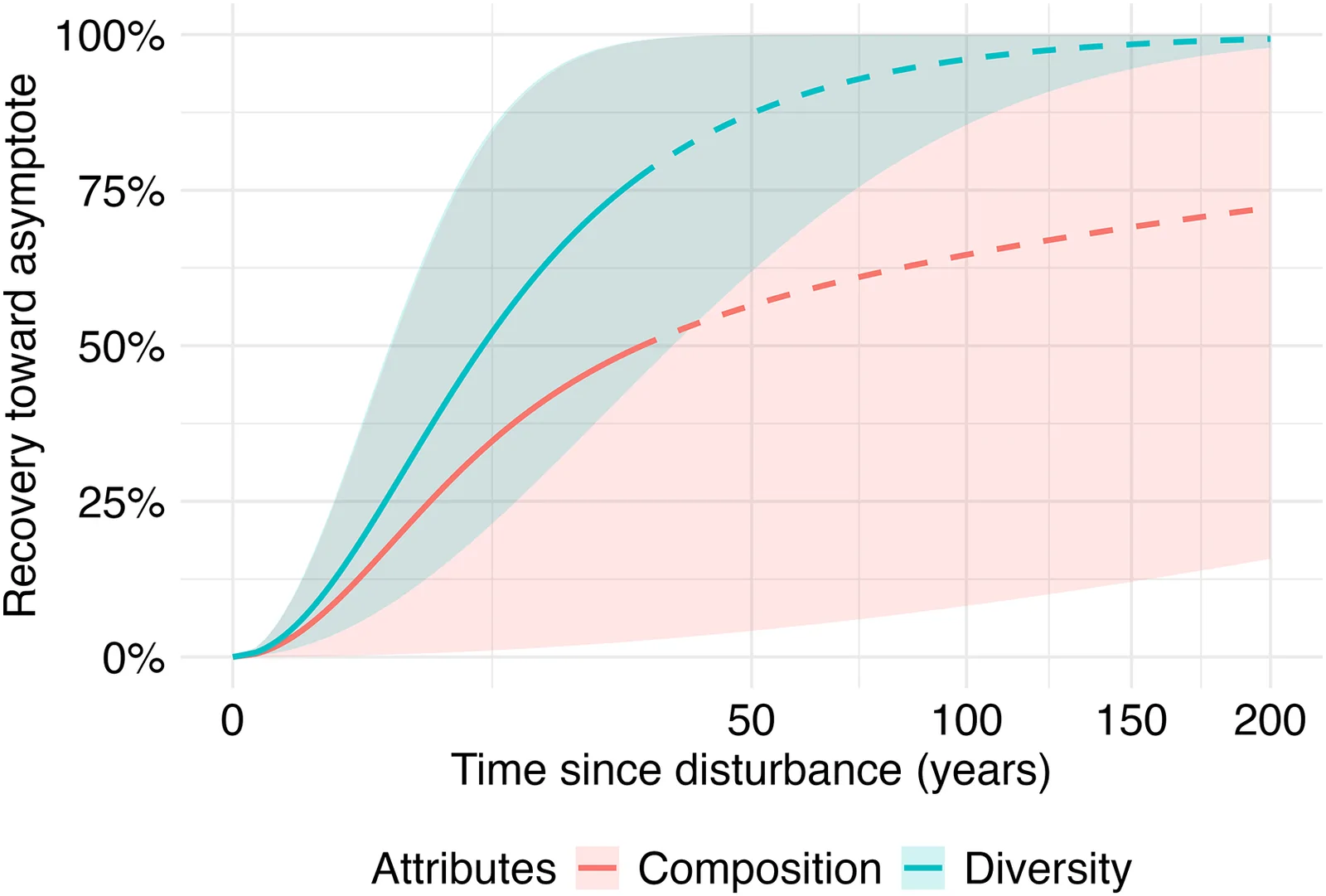
Modelled recovery trajectories of Shannon diversity and floristic composition in secondary tropical forests of West Africa (Taï National Park, Côte d’Ivoire). Lines represent posterior mean recovery trajectories estimated from the Bayesian exponential recovery model; shaded areas indicate 95% credible intervals. Solid lines correspond to the temporal range robustly informed by the underlying 9–26 year chronosequence (up to ~40 years since disturbance); dashed lines represent the model's extrapolation beyond this range, up to 200 years, and should be interpreted with caution given the increasing uncertainty. Recovery is expressed as the percentage of convergence toward old-growth reference conditions, with 100% corresponding to the asymptotic state observed in intact West African old-growth forests.

Posterior medians and 90% credible intervals (5th–95th percentiles) of the exponential recovery model parameters for Shannon diversity (Hill number of order 1) and floristic composition (abundance-based Chao–Jaccard similarity). $\theta_{\text{0}}$ represents the initial value of the recovery trajectory; $\theta_{+\infty }$ the asymptotic value corresponding to old-growth reference conditions; λ the intrinsic recovery rates; $\theta_{dist}$ the effect of past disturbance on the asymptotic value; and σ the residual variance of the log-normal likelihood. Posterior estimates of environmental covariate effects (β parameters) are not shown here and are presented in Fig 3.

**Fig 3:**
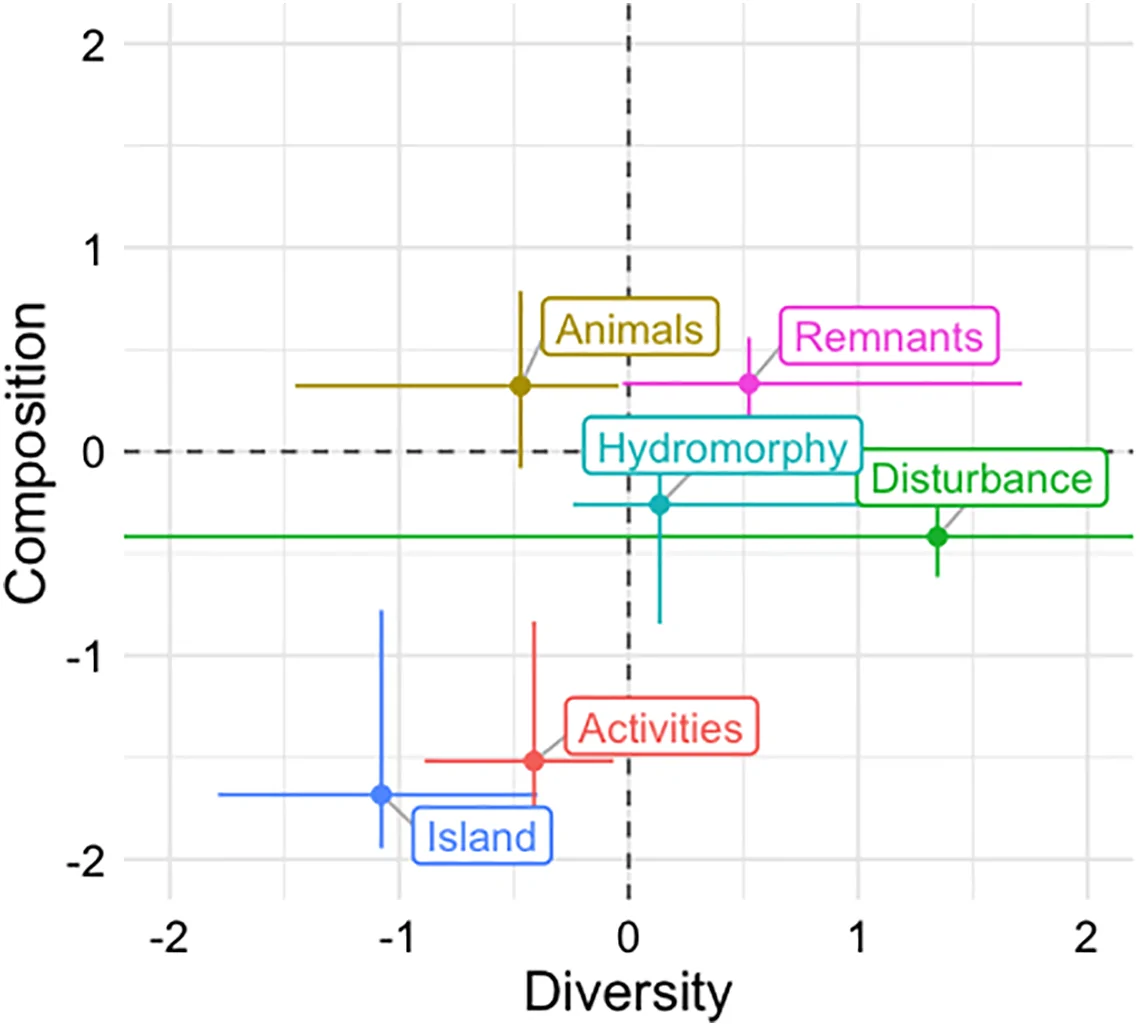
Environmental drivers of diversity and composition recovery rates. Points represent posterior mean effects of standardized covariates on the intrinsic recovery rate (λ) for diversity (x-axis) and composition (y-axis). Error bars indicate 95% credible intervals. Vertical and horizontal dashed lines represent zero effect. Positive values indicate an increase in recovery rate, whereas negative values indicate slower recovery. Covariates include animal presence, remnant tree density, soil hydromorphy, island location, human activities, and disturbance intensity.

Environmental covariates had contrasting effects on recovery rates (Fig 3). Remnant trees showed positive effects on both diversity and composition. Human activities and island configuration were associated with slower recovery of both attributes. Animal presence increased compositional recovery but was negatively associated with diversity recovery. Soil hydromorphy and disturbance exhibited variable effects, with wide credible intervals for diversity but negative effects on compositional recovery.

### Functional trait dynamics

Trait values varied jointly with stem diameter and forest age, with distinct convergence rates among traits (Table 3).

**Table 3:** Posterior summaries of the recovery model parameters for functional traits in secondary forests of West Africa.

Parameters	Seed mass			Wood density			Leaf mass per area		
	Median	5th	95th	Median	5th	95th	Median	5th	95th
α	20.43	19.89	21.01	0.61	0.61	0.62	60.12	59.63	60.60
β	1.26	0.41	2.11	−0.17	−0.18	−0.16	4.63	4.63	5.46
λ	1.21	0.72	2.25	0.15	0.15	0.16	1.4	0.9	2.47
θ	0.98	0.95	0.99	0.97	0.96	0.99	0.98	0.99	0.99
σ	2.85	2.81	2.89	0.22	0.22	0.23	3.62	3.5	3.69

Wood density showed a gradual increase with forest age and remained structured by stem diameter (Fig 4). Larger individuals exhibited slightly lower wood density values than smaller individuals, consistent with the negative size effect (β = −0.17). Convergence toward old-growth conditions was slow (λ = 0.15). The disturbance parameter (θ = 0.97) indicated slightly lower expected values under disturbance relative to intact old-growth forests.

**Fig 4:**
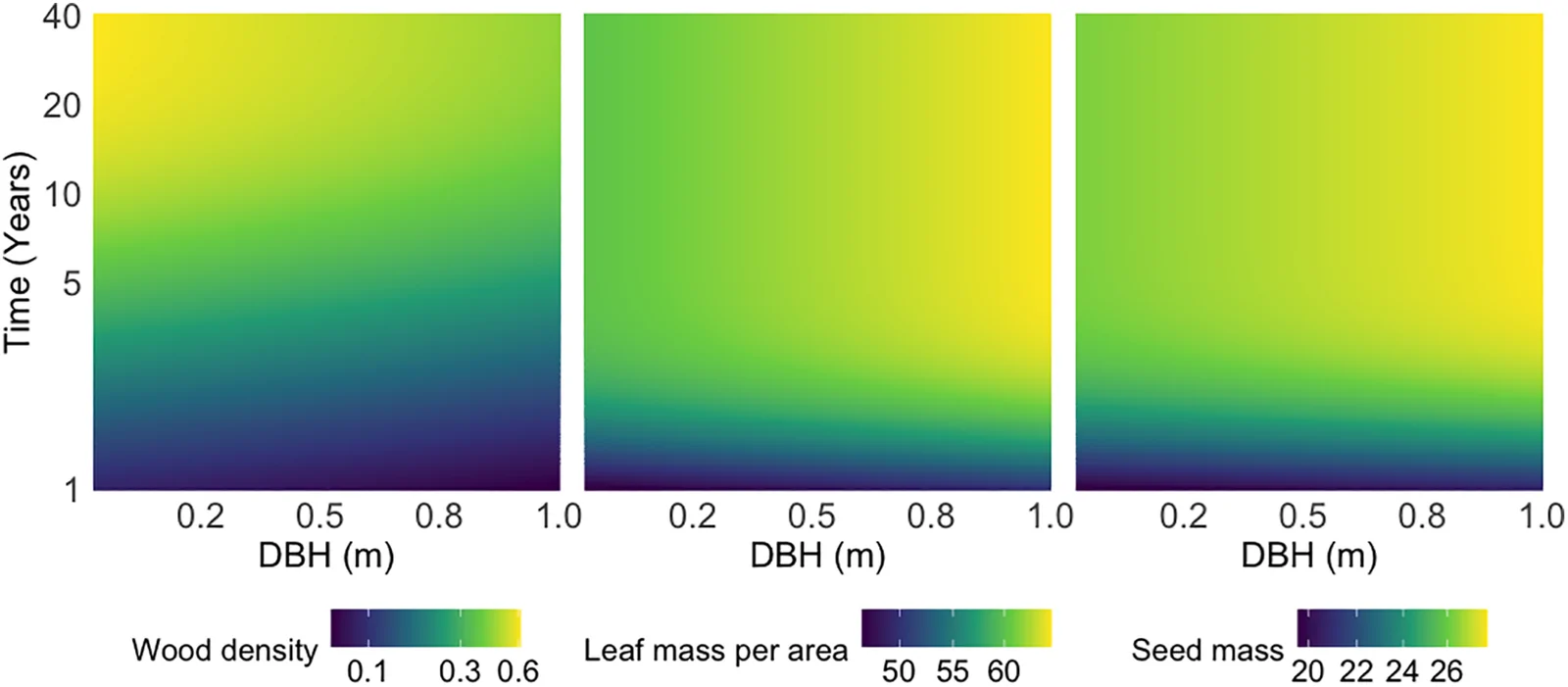
Modelled trajectories of functional trait distributions along the DBH and successional age gradients in secondary forests of West Africa. Heatmaps represent posterior mean predicted values of wood density (g.cm^-3^), leaf mass per area (LMA; g.m^-2^), and seed mass (mg) as functions of stem diameter (DBH, m; x-axis) and time since abandonment (years; y-axis). Color gradients indicate expected trait values derived from the individual-level convergence model. Predictions illustrate progressive alignment of trait–size relationships toward old-growth reference conditions as forest age increases.

LMA was strongly influenced by stem diameter, with larger trees displaying higher values (β = 4.63). In contrast to wood density, LMA converged rapidly toward old-growth reference conditions (λ = 1.40). Predicted values exceeded 60 g.m^-2^ within the first decade of succession across most of the DBH gradient (Fig 4). The disturbance effect was minimal (θ = 0.98).

Seed mass increased with stem diameter (β = 1.26) and exhibited rapid convergence dynamics (λ = 1.21). Predicted seed mass values for larger individuals exceeded 25 mg within the first 10 years of succession and remained stable thereafter (Fig 4). The disturbance parameter was close to unity (θ = 0.98; 90% CI: 0.95–0.99), indicating limited deviation from intact old-growth reference values.

Posterior medians and 90% credible intervals (5th–95th percentiles) of the individual-level exponential convergence model parameters for seed mass, wood density, and leaf mass per area. α represents the expected trait value when DBH = 0; β quantifies the effect of stem diameter on trait values; λ is the intrinsic convergence rate toward old-growth reference conditions; θ represents the multiplicative effect of disturbance on trait values in old-growth forests; and σ is the residual variance of the log-normal likelihood.

### Conservation-relevant species

Among the 174 recorded species, 16 (9.2%) were classified as either Endangered (EN) or Vulnerable (VU) according to the IUCN Red List (Appendix 1), and 44 species (25.3%) were identified as old-growth indicator species based on the IndVal analysis (Appendix 2).

IUCN-listed species were present across all forest stages. Their proportion remained relatively stable among young secondary forests (≤ 11 years), older secondary forests (> 11 years), and intact old-growth forests, with values ranging between 8% and 9% of individuals (Fig 5).

**Fig 5:**
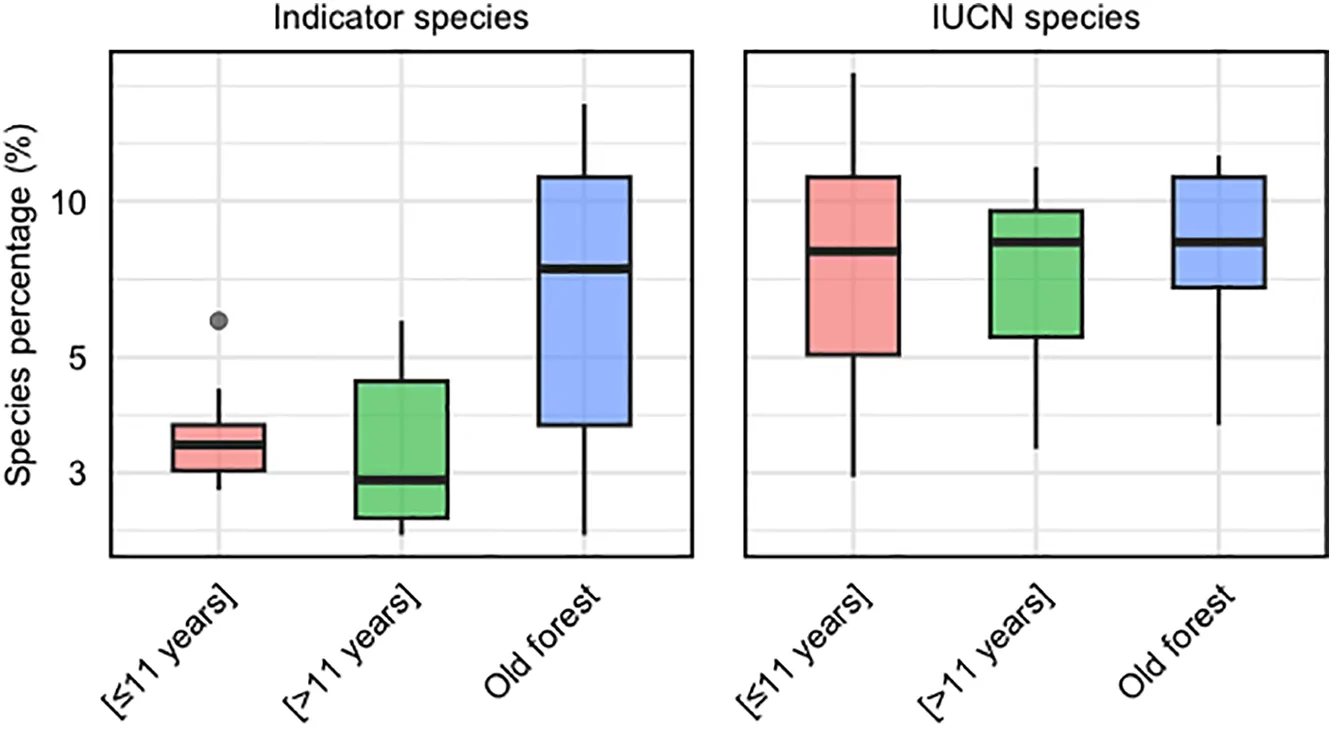
Proportion of conservation-relevant species across successional stages in West African tropical forests. Boxplots show the percentage of individuals belonging to old-growth indicator species (left panel) and IUCN-listed species categorized as Vulnerable or Endangered (right panel) across three forest categories: secondary forests ≤ 11 years since abandonment, secondary forests > 11 years, and intact old-growth forests. Boxes represent interquartile ranges, horizontal lines indicate medians, and whiskers show the range of observed values.

In contrast, the proportion of old-growth indicator species increased with forest age. Indicator species represented approximately 3–5% of individuals in young secondary forests, 4–6% in intermediate secondary forests, and 7–11% in old-growth forests (Fig 5).

## Discussion

In the last extensive block of Upper Guinean rainforest in West Africa, our results show that secondary forests surrounding Taï National Park retain substantial ecological potential. Although biodiversity dimensions recover at different rates, species diversity rebounds relatively quickly, functional attributes re-establish within decades, and conservation-relevant species persist across successional stages. These findings provide encouraging evidence that, when protected from further disturbance, naturally regenerating forests in this region can contribute meaningfully to biodiversity conservation and landscape resilience.

### Rapid diversity recovery masks slower and context-dependent compositional convergence

The rapid recovery of diversity likely reflects the resilience of secondary forests and the progressive accumulation of species following land abandonment [47]. Similar patterns have been reported in Neotropical forests, where up to 90% of species richness can recover within two decades [47–49]. In contrast, floristic composition depends on the return of late-successional species, many of which are dispersal-limited and characterized by larger seed mass [50,51]. As a result, compositional convergence may require several decades to centuries. Comparable trajectories have been observed in the Congo Basin [52], the Neotropics [53], and Southeast Asia [51], reinforcing the generality of this asynchronous recovery pattern.

Importantly, compositional recovery was not only slower but also highly variable, reflecting strong context dependence. It should be noted that the lower bound of the credible interval for the compositional recovery rate is very close to zero, meaning our data cannot exclude a near-negligible recovery rate that would be practically equivalent to stagnation over the sampled chronosequence (9–26 years); this uncertainty highlights the need for longer-term monitoring to distinguish slow recovery from an effective stall. Previous land use played a central role in shaping these recovery trajectories. Abandoned cocoa plantations often retain partial soil fertility [54] and remnant trees [55,56], which facilitate regeneration and canopy re-establishment [57,58]. In contrast, artisanal gold mining removes topsoil, disrupts seed banks, alters soil structure, and may lead to heavy metal accumulation, reducing microbial activity and long-term fertility [16,59]. Such disturbances strongly constrain recolonization by forest specialists and slow compositional convergence [40].

Environmental factors further modulated recovery trajectories. Remnant trees emerged as a key driver accelerating both diversity and compositional recovery. These trees create favorable microhabitats, increase structural heterogeneity, and act as local seed sources [54,60]. They also attract seed dispersers such as birds and bats, facilitating the recruitment of late-successional species [61,62]. Similar patterns have been documented elsewhere, where species richness and compositional similarity to primary forests increase around remnant trees after two decades of succession [63,64]. Animal presence showed contrasting effects on the two attributes, increasing compositional recovery while being negatively associated with diversity recovery (Fig 3); this is consistent with zoochorous dispersal selectively favouring a narrower set of heavy-seeded, shade-tolerant, mature-forest specialists, driving compositional convergence at the expense of the many wind- and self-dispersed generalist pioneers that inflate species diversity in less animal-frequented secondary forests. Conversely, human activities and forest isolation slowed recovery. Hunting and logging associated with gold mining reduce populations of large seed dispersers, thereby limiting recruitment of large-seeded forest species [65,66]. Forests on islands created a fragmentation experience that reduced propagule influx and strong edge effects, favoring pioneer and generalist species [67–69]. In line with island biogeography theory [70], limited connectivity constrains long-term compositional recovery. Hydromorphic soils also exerted contrasting effects, promoting herbaceous dominance (e.g., Marantaceae) that can inhibit woody recruitment and slow structural and compositional reassembly [58,71–73].

Together, these results show that while diversity rebounds relatively quickly in regenerating forests of West Africa, floristic composition follows a slower and highly context-dependent trajectory. Around Taï National Park, this asynchrony underscores the importance of maintaining landscape connectivity, remnant trees, and effective protection from continued disturbance to support long-term compositional recovery.

### Rapid reassembly of leaf and regeneration traits contrasts with slow structural maturation

Functional traits recovered at markedly different rates, revealing distinct ecological dimensions of succession in the forests surrounding Taï National Park. In intact old-growth forests, trait distributions reflect conservative resource-use strategies adapted to closed-canopy conditions, where light is limiting and competition for water and nutrients is intense [74]. Dominant species tend to be shade tolerant and invest in durable tissues, including dense wood and structurally robust leaves.

In contrast, secondary forests initially host species that prioritize rapid growth over structural investment. The lower wood density observed in younger forests likely reflects the dominance of pioneer species that colonized soon after abandonment. These heliophilous species capitalize on high light availability created by disturbance, investing in fast height growth rather than dense, mechanically resistant wood. As succession proceeds and canopy closure increases, later-arriving species face reduced light and heightened resource competition [75]. These species typically allocate more biomass to dense wood, enhancing mechanical stability and resistance to disturbance [76]. Such ontogenetic and successional shifts in species composition likely explain the slow convergence of wood density, consistent with broader evidence that structural maturation in tropical forests requires several decades [20,77].

In contrast, leaf mass per area (LMA) reassembled rapidly. LMA increased with both forest age and stem diameter, reflecting adjustments to vertical light gradients and hydraulic constraints. Leaves positioned higher in the canopy experience greater irradiance and wind exposure, promoting thicker and denser leaf construction [78,79]. Increased hydraulic path length in taller individuals further constrains water transport to the canopy, favoring structural reinforcement of leaves. Smaller trees and understory individuals, by contrast, operate under lower light intensity and shorter hydraulic distances, allowing thinner and lighter leaves. The rapid convergence of LMA suggests that canopy stratification and associated microclimatic gradients re-establish relatively quickly during succession. Because LMA is assigned as a fixed species-level value to every individual of a given species, rather than measured directly on individual trees, our data cannot capture in situ or ontogenetic adjustment of leaf traits within individuals. The observed convergence in LMA therefore necessarily reflects progressive species turnover toward taxa with characteristically higher LMA, not physiological or plastic adaptation of existing trees. In addition, LMA values were available for only 56% of individuals, and species lacking a trait record were excluded from this analysis; while we have no indication of systematic bias in which species were missing, this limited coverage should be kept in mind when assessing the generality of the LMA convergence pattern.

Seed mass dynamics reflect a distinct regeneration axis. Pioneer species typically produce numerous small and light seeds that disperse effectively into disturbed environments [28,80]. Although these seeds contain limited reserves, they benefit from high-light conditions and rapid germination. In contrast, shade-tolerant species produce fewer but larger seeds rich in reserves, enhancing seedling survival under low-light and competitive understory conditions [81–83]. Small-seeded species compensate for lower individual survival through high fecundity and persistence in soil seed banks [80,84]. The relatively rapid stabilization of seed mass distributions indicates that regeneration strategies diversify early in succession, even before full structural maturation of the forest.

Together, these patterns show that while leaf-level adjustments and regeneration strategies reassemble within decades, structural attributes associated with long-lived woody tissues recover more slowly. Around Taï National Park, this decoupling between rapid functional adjustment and delayed structural maturation highlights that forest recovery involves multiple ecological dimensions operating on different temporal scales.

### Naturally regenerating forests contribute meaningfully to conservation

The presence of IUCN-listed species across all forest stages indicates that naturally regenerating forests around Taï National Park maintain conservation value throughout succession. The proportion of Endangered and Vulnerable species remained relatively stable across young secondary, older secondary, and old-growth forests, suggesting that early successional habitats contribute to sustaining threatened taxa. Similar complementary roles of secondary forests have been reported in other tropical landscapes [85–87].

In contrast, old-growth indicator species were markedly more abundant in intact forests than in secondary stands (Fig 5). Although indicator species were present in both young and older secondary forests likely reflecting the persistence of remnant trees [88], their proportion remained substantially lower than in old-growth forests. This pattern suggests that while secondary forests support elements of primary forest composition, full recovery of old-growth identity requires longer time spans and sustained protection. Comparable trends have been documented in West Africa, where indicator species progressively increase but remain less abundant in secondary stands than in primary forests [72].

Together, these findings highlight the complementary but not substitutive role of secondary forests in the Upper Guinean region. While they do not yet replicate the full compositional integrity of old-growth forests, their capacity to maintain threatened species and retain primary-forest elements underscores their importance for biodiversity conservation in West Africa.

## Conclusion

Following episodes of intense degradation during the post-electoral crises, the forests surrounding Taï National Park have undergone a decade of natural regeneration. Our results show that this regeneration is not merely structural [58,89] but ecologically meaningful. Secondary forests recover species diversity rapidly, reassemble key functional attributes within decades, and retain threatened taxa across successional stages. Although full compositional convergence toward old-growth forests remains slower and strongly context-dependent, the progressive return of primary-forest elements demonstrates substantial ecological resilience in the last major block of Upper Guinean rainforest. In a region where deforestation has drastically reduced forest cover, these findings provide concrete evidence that protecting regenerating forests around Taï is not a secondary objective but a central pillar of long-term conservation. Safeguarding both intact old-growth stands and naturally recovering forests is essential to maintain biodiversity, ecological functions, and landscape connectivity in West Africa.

## Supporting information

S1 Appendix IUCN-listed tree species recorded in Taï National Park (Côte d’Ivoire). Species classified as Endangered (EN) or Vulnerable (VU) according to the IUCN Red List. Values of 1 indicate the assigned conservation category.
(DOCX)

S2 Appendix Old-growth indicator species identified using the Dufrêne–Legendre indicator value method. Indicator species associated with intact old-growth forests, identified using the Dufrêne–Legendre indicator value (IndVal) method. Only species with significant association (P < 0.002) are reported. IndVal values range from 0 (no association) to 1 (perfect specificity and fidelity to old-growth forests).
(DOCX)

## References

[1] Myers N, Mittermeier RA, Mittermeier CG, da Fonseca GA, Kent J. Biodiversity hotspots for conservation priorities. Nature. 2000;403(6772):853–8. doi: 10.1038/35002501 10706275

[2] Sechrest W, Brooks TM, da Fonseca GAB, Konstant WR, Mittermeier RA, Purvis A, et al. Hotspots and the conservation of evolutionary history. Proc Natl Acad Sci U S A. 2002;99(4):2067–71. doi: 10.1073/pnas.251680798 11854502 PMC122320

[3] Barima YSS, Kouakou ATM, Bamba I, Sangne YC, Godron M, Andrieu J. Cocoa crops are destroying the forest reserves of the classified forest of Haut-Sassandra (Ivory Coast). Glob Ecol Conserv. 2016;8:85–98. doi: 10.1016/j.gecco.2016.08.009

[4] Ruf F, Koné D. Climate change, cocoa migrations and deforestation in West Africa: What does the past tell us about the future. Sustain Sci. 2015;10:101–11. doi: 10.1007/s11625-014-0282-4

[5] Sanial E, Ruf F, Louppe D, Mietton M, Hérault B. Local farmers shape ecosystem service provisioning in West African cocoa agroforests. Agrofor Syst. 2023;97(3):401–14. doi: 10.1007/s10457-021-00723-6

[6] Depecker J, Asimonyio JA, Miteho R, Hatangi Y, Kambale JL, Verleysen L. The association between rainforest disturbance and recovery, tree community composition, and community traits in the Yangambi area in the Democratic Republic of the Congo. J Trop Ecol. 2022;38(6):426–36. doi: 10.1017/S0266467422000347

[7] Chazdon RL. Tropical forest recovery: legacies of human impact and natural disturbances. Perspect Plant Ecol Evol Syst. 2003;6(1–2):51–71. doi: 10.1078/1433-8319-00042

[8] Chazdon RL. Second growth: The promise of tropical forest regeneration in an age of deforestation. Second growth. University of Chicago Press. 2014.

[9] Évaluation des ressources forestières mondiales. Rome: FAO. 2020.

[10] Brancalion PHS, Niamir A, Broadbent E, Crouzeilles R, Barros FSM, Almeyda Zambrano AM, et al. Global restoration opportunities in tropical rainforest landscapes. Sci Adv. 2019;5(7):eaav3223. doi: 10.1126/sciadv.aav3223 31281881 PMC6609219

[11] Teixeira HM, Cardoso IM, Bianchi FJJA, da Cruz Silva A, Jamme D, Peña-Claros M. Linking vegetation and soil functions during secondary forest succession in the Atlantic forest. For Ecol Manag. 2020;457:117696. doi: 10.1016/j.foreco.2019.117696

[12] Aide TM, Clark ML, Grau HR, López-Carr D, Levy MA, Redo D. Deforestation and reforestation of Latin America and the Caribbean (2001–2010). Biotropica. 2013;45(2):262–71. doi: 10.1111/j.1744-7429.2012.00908.x

[13] Arroyo-Rodríguez V, Melo FPL, Martínez-Ramos M, Bongers F, Chazdon RL, Meave JA, et al. Multiple successional pathways in human-modified tropical landscapes: New insights from forest succession, forest fragmentation and landscape ecology research. Biol Rev Camb Philos Soc. 2017;92(1):326–40. doi: 10.1111/brv.12231 26537849

[14] Chazdon RL, Finegan B, Capers RS, Salgado‐Negret B, Casanoves F, Boukili V, et al. Composition and dynamics of functional groups of trees during tropical forest succession in Northeastern Costa Rica. Biotropica. 2010;42(1):31–40. doi: 10.1111/j.1744-7429.2009.00566.x

[15] Poorter L, Craven D, Jakovac CC, van der Sande MT, Amissah L, Bongers F, et al. Multidimensional tropical forest recovery. Science. 2021;374(6573):1370–6. doi: 10.1126/science.abh3629 34882461

[16] Peterson GD, Heemskerk M. Deforestation and forest regeneration following small-scale gold mining in the Amazon: The case of Suriname. Environmental Conservation. 2001;28(2):117–26. doi: 10.1017/S0376892901000121

[17] Derroire G, Tigabu M, Odén PC, Healey JR. The effects of established trees on woody regeneration during secondary succession in tropical dry forests. Biotropica. 2016;48(3):290–300. doi: 10.1111/btp.12287

[18] Amani BHK, N’Guessan AE, Derroire G, N’dja JK, Elogne AGM, Traoré K, et al. The potential of secondary forests to restore biodiversity of the lost forests in semi-deciduous West Africa. Biological Conservation. 2021;259:109154. doi: 10.1016/j.biocon.2021.109154

[19] Wright SJ, Kitajima K, Kraft NJB, Reich PB, Wright IJ, Bunker DE, et al. Functional traits and the growth-mortality trade-off in tropical trees. Ecology. 2010;91(12):3664–74. doi: 10.1890/09-2335.1 21302837

[20] Poorter L, Rozendaal DMA, Bongers F, de Almeida-Cortez JS, Almeyda Zambrano AM, Álvarez FS, et al. Wet and dry tropical forests show opposite successional pathways in wood density but converge over time. Nat Ecol Evol. 2019;3(6):928–34. doi: 10.1038/s41559-019-0882-6 31011177

[21] Lavorel S, Garnier E. Predicting changes in community composition and ecosystem functioning from plant traits: Revisiting the holy grail. Funct Ecol. 2002;16(5):545–56. doi: 10.1046/j.1365-2435.2002.00664.x

[22] Chave J, Coomes D, Jansen S, Lewis SL, Swenson NG, Zanne AE. Towards a worldwide wood economics spectrum. Ecol Lett. 2009;12(4):351–66. doi: 10.1111/j.1461-0248.2009.01285.x 19243406

[23] Poorter L. The relationships of wood-, gas- and water fractions of tree stems to performance and life history variation in tropical trees. Ann Bot. 2008;102(3):367–75. doi: 10.1093/aob/mcn103 PMC2701806 18573862

[24] Schmitt S, Maréchaux I, Chave J, Fischer FJ, Piponiot C, Traissac S. Functional diversity improves tropical forest resilience: Insights from a long‐term virtual experiment. Journal of Ecology. 2020;108(3):831–43. doi: 10.1111/1365-2745.13320

[25] Herault B, Ouallet J, Blanc L, Wagner F, Baraloto C. Growth responses of neotropical trees to logging gaps. Journal of Applied Ecology. 2010;47(4):821–31. doi: 10.1111/j.1365-2664.2010.01826.x

[26] Baraloto C, Forget PF, Goldberg DE. Seed mass, seedling size and neotropical tree seedling establishment. J Ecol. 2005;93(6):1156–66. doi: 10.1111/j.1365-2745.2005.01041.x

[27] Kitajima K. Do shade-tolerant tropical tree seedlings depend longer on seed reserves? Functional growth analysis of three Bignoniaceae species. Funct Ecol. 2002;16(4):433–44. doi: 10.1046/j.1365-2435.2002.00641.x

[28] Moles AT, Falster DS, Leishman MR, Westoby M. Small-seeded species produce more seeds per square metre of canopy per year, but not per individual per lifetime. J Ecol. 2004;92(3):384–96. doi: 10.1111/j.0022-0477.2004.00880.x

[29] Doua-Bi GYA, Zo-Bi IC, Amani BHK, Elogne AGM, N’dja JK, N’Guessan AE, et al. Taking advantage of natural regeneration potential in secondary forests to recover commercial tree resources in Côte d’Ivoire. For Ecol Manag. 2021;493(May):119240. doi: 10.1016/j.foreco.2021.119240

[30] Châtelain C, Aké-Assi L, Spichiger R, Gautier L. Cartes de distribution des plantes de Côte d’Ivoire. Suisse: Conservatoire et Jardin botanique de la Ville de Genève. 2011.

[31] Plan d’Aménagement et de Gestion (PAG) du Parc national de Taï 2014-2018. OIPR. 2014.

[32] Plan d’aménagement et de gestion du parc national de Taï (PAG) 2020-2029. Abidjan, Côte d’Ivoire. 2020.

[33] Kouamélan EP. Biodiversité des poissons du parc national de Taï: une ressource naturelle à sauvegarder. Abidjan: Éditionns Universitaires Européennes. 2018.

[34] Van Rompaey R, Adou Yao C, Déngueadhé KBE, Aké-Assi L, Wittebolle G, N’Guessan K. Biodiversité végétale et écologie du paysage dans le Sud du Parc national de Taï (Côte d’Ivoire). Wageningen, Pays-Bas: Programme Tropenbos Côte d’Ivoire. 2001.

[35] Bakayoko A, Neuba DFR, Kouadio YL, Nehoun B. Suivi de la restauration végétale naturelle des zones aménagées dans les secteurs de Djapadji, Soubré et ADK/V6 du Parc national de Taï. Abidjan (Côte d’Ivoire). 2018.

[36] Aké-Assi L. Flore de la Côte-d’Ivoire: Catalogue Systématique, Biogéographie et Écologie. Conservatoire et Jardin botanique de la Ville de Genève. 2001.

[37] Hutchinson J, Dalziel JM. Flora of West Tropical Africa. 2nd ed. Keay RWJ, Hepper FN, editors. London: Crown Agents for Overseas Governments and Administrations. 1954.

[38] Réjou‐Méchain M, Tanguy A, Piponiot C, Chave J, Hérault B. biomass: An r package for estimating above‐ground biomass and its uncertainty in tropical forests. Methods Ecol Evol. 2017;8(9):1163–7. doi: 10.1111/2041-210x.12753

[39] Kattge J, Bönisch G, Díaz S, Lavorel S, Prentice IC, Leadley P, et al. TRY plant trait database – enhanced coverage and open access. Glob Change Biol. 2020;26(1):119–88. doi:10.1111/gcb.14904 31891233

[40] Dago MR, Zo-Bi IC, Badouard V, Patacca M, Hérault B. Concomitant effects of multiple disturbances (logging, fire, biological invasion) on native tree abundances into West Africa’s semi-deciduous forests. Biol Conserv. 2023;285:1101220. doi:10.1016/j.biocon.2023.110220

[41] De La Mensbruge G. La germination et les plantules des essences arborées de la forêt dense humide de la Côte d’Ivoire. CTFT. 1966.

[42] Dufrene M, Legendre P. Species assemblages and indicator species: The need for a flexible asymmetrical approach. Ecological Monographs. 1997;67(3):345. doi: 10.2307/2963459

[43] Marcon E, Hérault B. Entropart: An R package to measure and partition diversity. J Stat Softw. 2015;67:1–26. doi: 10.18637/jss.v067.i08

[44] Maurent E, Hérault B, Piponiot C, Derroire G, Delgado D, Finegan B. A common framework to model recovery in disturbed tropical forests. Ecol Model. 2023;483:110418. doi: 10.1016/J.ECOLMODEL.2023.110418

[45] Carpenter B, Gelman A, Hoffman MD, Lee D, Goodrich B, Betancourt M, et al. Stan: A probabilistic programming language. J Stat Softw. 2017;76:1. doi: 10.18637/jss.v076.i01 36568334 PMC9788645

[46] Hérault B, Piponiot C. Key drivers of ecosystem recovery after disturbance in a neotropical forest: Long-term lessons from the Paracou experiment, French Guiana. For Ecosyst. 2018;5(1):2. doi: 10.1186/s40663-017-0126-7

[47] Barros TC, Elias F, Romano LL, Ferreira J. Natural recovery of plant species diversity in secondary forests in Eastern Amazonia: Contributions to passive forest restoration. Braz J Bot. 2020;43(1):165–75. doi: 10.1007/s40415-020-00585-9

[48] Chazdon RL, Letcher SG, van Breugel M, Martínez-Ramos M, Bongers F, Finegan B. Rates of change in tree communities of secondary Neotropical forests following major disturbances. Philos Trans R Soc Lond B Biol Sci. 2007;362(1478):273–89. doi: 10.1098/rstb.2006.1990 17255036 PMC2311434

[49] Lennox GD, Gardner TA, Thomson JR, Ferreira J, Berenguer E, Lees AC, et al. Second rate or a second chance? Assessing biomass and biodiversity recovery in regenerating Amazonian forests. Glob Chang Biol. 2018;24(12):5680–94. doi: 10.1111/gcb.14443 30216600

[50] Chazdon RL, Norden N, Colwell RK, Chao A. Monitoring recovery of tree diversity during tropical forest restoration: Lessons from long-term trajectories of natural regeneration. Philos Trans R Soc Lond B Biol Sci. 2023;378(1867):20210069. doi: 10.1098/rstb.2021.0069 36373917 PMC9661944

[51] Deng F, He Y, Zang R. Recovery of functional diversity following shifting cultivation in tropical monsoon forests. Forests. 2018;9(9):506. doi: 10.3390/f9090506

[52] Makelele IA, Verheyen K, Boeckx P, Cizungu Ntaboba L, Mujinya Bazirake B, Ewango C, et al. Afrotropical secondary forests exhibit fast diversity and functional recovery, but slow compositional and carbon recovery after shifting cultivation. J Veg Sci. 2021;32(5):e13071. doi: 10.1111/jvs.13071

[53] Rozendaal DMA, Bongers F, Aide TM, Alvarez-Dávila E, Ascarrunz N, Balvanera P, et al. Biodiversity recovery of Neotropical secondary forests. Sci Adv. 2019;5(3):eaau3114. doi: 10.1126/sciadv.aau3114 30854424 PMC6402850

[54] Koffi AB, Tondoh EJ, Ehouman E, Freycon V, Piponiot C, Amani BHK. Drivers of soil organic carbon recovery during secondary succession along a West African climate gradient. Ecol Indic. 2025;178:1–11. doi: 10.1016/j.ecolind.2025.114073

[55] Konan IK, N’Guessan AE, Dago MR, Kouassi A, N’dja JK, Aussenac R. Maximizing tree diversity in cocoa agroforestry: Taking advantage of planted, spontaneous, and remnant trees. Agrofor Syst. 2025;99(5):1–14. doi: 10.1007/s10457-025-01180-1

[56] Kouassi AK, Zo-Bi IC, Aussenac R, Kouamé IK, Dago MR, N’guessan AE, et al. The great mistake of plantation programs in cocoa agroforests – Let’s bet on natural regeneration to sustainably provide timber wood. Trees, Forests and People. 2023;12:100386. doi: 10.1016/j.tfp.2023.100386

[57] N’Guessan AE, Hérault B, Zo-Bi IC, Aussenac R, Dago M-R, Konan IK, et al. Rethinking cocoa agroforestry: Towards sustainable coexistence of remnant, spontaneous and planted trees. Perspect. 2026;(70):1–4. doi: 10.19182/perspective/38076

[58] Houphouët ADL, Sangne YC, Diarrassouba A, Adou Yao YC, Betbeder J, Hérault B. Forest structure recovery around West Africa’s last great rainforest: Modelling complex dynamics in Taï national park. Trees, Forests and People. 2025;22:101005. doi: 10.1016/j.tfp.2025.101005

[59] Albert KM. Role of revegetation in restoring fertility of degraded mined soils in Ghana: A review. Int J Biodivers Conserv. 2015;7(2):57–80. doi: 10.5897/IJBC2014.0775

[60] Pérez-Cárdenas N, Mora F, Arreola-Villa F, Arroyo-Rodríguez V, Balvanera P, Flores-Casas R. Effects of landscape composition and site land-use intensity on secondary succession in a tropical dry forest. Forest Ecology and Management. 2021;482:118818. doi: 10.1016/j.foreco.2020.118818

[61] Tucker MM, Kashian DM. Pre-fire forest remnants affect post-fire plant community structure and composition. For Ecol Manag. 2018;408:103–11. doi: 10.1016/J.FORECO.2017.10.038

[62] Wu MM, Liang Y, He HS, Liu B, Ma T. Remnant trees location and abundance play different roles in forest landscape recovery. For Ecol Manag. 2022;511:120154. doi: 10.1016/j.foreco.2022.120154

[63] Sandor ME, Chazdon RL. Remnant trees affect species composition but not structure of tropical second-growth forest. PLoS One. 2014;9(1):e83284. doi: 10.1371/journal.pone.0083284 24454700 PMC3890367

[64] Sterck FJ, Poorter L, Schieving F. Leaf traits determine the growth-survival trade-off across rain forest tree species. Am Nat. 2006;167(5):758–65. doi: 10.1086/503056 16671019

[65] Brodie JF, Helmy OE, Brockelman WY, Maron JL. Bushmeat poaching reduces the seed dispersal and population growth rate of a mammal-dispersed tree. Ecol Appl. 2009;19(4):854–63. doi: 10.1890/08-0955.1 19544729

[66] Markl JS, Schleuning M, Forget PM, Jordano P, Lambert JE, Traveset A, et al. Meta-analysis of the effects of human disturbance on seed dispersal by animals. Conserv Biol. 2012;26(6):1072–81. doi: 10.1111/j.1523-1739.2012.01927.x 22971077

[67] Laurance WF. Theory meets reality: How habitat fragmentation research has transcended island biogeographic theory. Biol Conserv. 2008;141(7):1731–44. doi: 10.1016/j.biocon.2008.05.011

[68] Murcia C. Edge effects in fragmented forests: Implications for conservation. Trends Ecol Evol. 1995;10(2):58–62. doi: 10.1016/S0169-5347(00)88977-6 21236953

[69] Traoré S, Zo-Bi IC, Piponiot C, Aussenac R, Hérault B. Fragmentation is the main driver of residual forest aboveground biomass in West African low forest-high deforestation landscapes. Trees, Forests and People. 2024;15:100477. doi: 10.1016/j.tfp.2023.100477

[70] MacArthur RH, Wilson EO. The Theory of Island Biogeography. Princeton University Press. 2001.

[71] Cuni-Sanchez A, White LJT, Calders K, Jeffery KJ, Abernethy K, Burt A. African savanna-forest boundary dynamics: A 20-year study. PLOS ONE. 2016;11(6):156934. doi: 10.1371/journal.pone.0156934 PMC4919100 27336632

[72] Amani BHK, N’Guessan AE, Van der Meersch V, Derroire G, Piponiot C, Elogne AGM. Lessons from a regional analysis of forest recovery trajectories in West Africa. Environmental Research Letters. 2022;17(11). doi: 10.1088/1748-9326/ac9b4f

[73] Pouteau R, Picard J, Doumenge C, Brncic T, Gillet J-F, Doucet J-L, et al. The puzzling ecology of African Marantaceae forests. Am J Bot. 2024;111(4):e16320. doi: 10.1002/ajb2.16320 38629307

[74] Poorter L, Rozendaal DMA, Bongers F, Almeida de JS, Álvarez FS, Andrade JL, et al. Functional recovery of secondary tropical forests. Proc Natl Acad Sci U S A. 2021;118(49):e2003405118. doi: 10.1073/pnas.2003405118 34845017 PMC8670493

[75] Liang J, Crowther TW, Picard N, Wiser S, Zhou M, Alberti G, et al. Positive biodiversity-productivity relationship predominant in global forests. Science. 2016;354(6309):aaf8957. doi: 10.1126/science.aaf8957 27738143

[76] Hérault B, Bachelot B, Poorter L, Rossi V, Bongers F, Chave J. Functional traits shape ontogenetic growth trajectories of rain forest tree species. J Ecol. 2011;99(6):1431–40. doi: 10.1111/j.1365-2745.2011.01883.x

[77] Norden N, Chazdon RL, Chao A, Jiang Y-H, Vílchez-Alvarado B. Resilience of tropical rain forests: tree community reassembly in secondary forests. Ecol Lett. 2009;12(5):385–94. doi: 10.1111/j.1461-0248.2009.01292.x 19379133

[78] Cavaleri MA, Oberbauer SF, Clark DB, Clark DA, Ryan MG. Height is more important than light in determining leaf morphology in a tropical forest. Ecology. 2010;91(6):1730–9. doi: 10.1890/09-1326.1 20583714

[79] Coble AP, Cavaleri MA. Light acclimation optimizes leaf functional traits despite height-related constraints in a canopy shading experiment. Oecologia. 2015;177(4):1131–43. doi: 10.1007/s00442-015-3219-4 25596955

[80] Dalling JW, Hubbell SP, Silvera K. Seed dispersal, seedling establishment and gap partitioning among tropical pioneer trees. J Ecol. 1998;86(4):674–89. doi: 10.1046/j.1365-2745.1998.00298.x

[81] Peña-Claros M, Boo HD. The effect of forest successional stage on seed removal of tropical rain forest tree species. J Trop Ecol. 2002;18(2):261–74. doi: 10.1017/S0266467402002171

[82] Poorter L, Rose SA. Light-dependent changes in the relationship between seed mass and seedling traits: A meta-analysis for rain forest tree species. Oecologia. 2005;142(3):378–87. doi: 10.1007/s00442-004-1732-y 15503163

[83] Konaté I, Ehouman E, Wourro F, Doua-Bi Y, Tiéoulé F, Coulibaly B. The anatomy of survival — What determines seedling fate after planting in West Africa? For Ecol Manag. 2025;598:123266. doi: 10.1016/j.foreco.2025.123266

[84] Muller-Landau HC. The tolerance-fecundity trade-off and the maintenance of diversity in seed size. Proc Natl Acad Sci U S A. 2010;107(9):4242–7. doi: 10.1073/pnas.0911637107 20160078 PMC2840174

[85] Banks-Leite C, Ewers RM, Kapos V, Martensen AC, Metzger JP. Comparing species and measures of landscape structure as indicators of conservation importance. J Appl Ecol. 2011;48(3):706–14. doi: 10.1111/j.1365-2664.2011.01966.x

[86] Barlow J, Gardner TA, Araujo IS, Avila-Pires TC, Bonaldo AB, Costa JE, et al. Quantifying the biodiversity value of tropical primary, secondary, and plantation forests. Proc Natl Acad Sci U S A. 2007;104(47):18555–60. doi: 10.1073/pnas.0703333104 18003934 PMC2141815

[87] Swanson ME, Franklin JF, Beschta RL, Crisafulli CM, DellaSala DA, Hutto RL. The forgotten stage of forest succession: Early-successional ecosystems on forest sites. Front Ecol Environ. 2011;9(2):117–25. doi: 10.1890/090157

[88] Seidl R, Rammer W, Spies TA. Disturbance legacies increase the resilience of forest ecosystem structure, composition, and functioning. Ecol Appl. 2014;24(8):2063–77. doi: 10.1890/14-0255.1 27053913 PMC4820056

[89] N’Guessan AE, N’dja JK, Yao ON, Amani BHK, Gouli RGZ, Piponiot C, et al. Drivers of biomass recovery in a secondary forested landscape of West Africa. Forest Ecology and Management. 2019;433:325–31. doi: 10.1016/j.foreco.2018.11.021

