# Supplementary material for: Regenerating forests around Taï National Park sustain biodiversity despite constrained floristic recovery
Source: PLoS One. 2026 Oct 21;21(10):e0356900. doi: 10.1371/journal.pone.0356900 (PMC13686277; doi:10.1371/journal.pone.0356900)
Supplement: Supplementary file 1 [file pone.0356900.s004.pdf]

PLOS One

Regenerating forests around Taï National Park sustain biodiversity despite constrained floristic recovery

--Manuscript Draft--

**Manuscript Number:**	PONE-D-26-18781R1
**Article Type:**	Research Article
**Full Title:**	Regenerating forests around Taï National Park sustain biodiversity despite constrained floristic recovery
**Short Title:**	Biodiversity rebounds but composition lags in regenerating West African forests
**Corresponding Author:**	Bruno Hérault<br>CIRAD<br>Montpellier, FRANCE
**Keywords:**	forest restoration; land-use legacy; ecosystem resilience; Protected area management; nature-based solutions; West Africa
**Abstract:**	Extensive deforestation and disturbance have reshaped forest landscapes in West Africa, including areas surrounding Taï National Park, the largest remaining block of Upper Guinean rainforest. Although many degraded areas are currently undergoing regeneration, the capacity of these secondary forests to recover their biodiversity attributes remains insufficiently assessed within this park. This study quantifies the multidimensional restoration of biodiversity across 125 secondary forest plots and 15 primary forest plots representing 14,731 inventoried individuals in four sectors of the park. Using a Bayesian modelling framework, we estimate recovery trajectories of Shannon diversity, floristic composition, functional traits (wood density, leaf mass per area, seed mass), and conservation-relevant species. The different biodiversity attributes recovered at contrasting rates. Diversity was restored more rapidly ($\lambda = 0.06$) than floristic composition ($\lambda = 0.03$), and these recovery varied according to environmental variables. Among these, the presence of remnant trees showed the highest median on diversity (0.53 ± 0.61) and floristic composition (0.37 ± 0.17), promoting the rapid recovery of both attributes, followed by prior land use, particularly cocoa farming which also positively influenced the recovery of alpha diversity ($\lambda = 0.03$) and floristic composition ($\lambda = 0.02$). Regarding functional traits, they displayed contrasting dynamics: specific leaf area and seed mass recovered rapidly along successional gradients, whereas wood density followed a more gradual recovery trajectory. From a conservation perspective, although the proportion of IUCN Red List species remained stable along the successional gradient, old-growth indicator species were significantly more abundant in primary forests. To ensure the long-term conservation of biodiversity, protecting both primary and regenerating forests is essential to preserve the ecological resilience of this park.
**Order of Authors:**	Aya Diane Larissa Houphouët, MSc
	Yao Charles Sangne
	Abdoulaye Diarassouba
	Evans Ehouman
	Bruno Hérault
**Response to Reviewers:**	See the cover letter in PDF for a detailed response
**Additional Information:**	
**Question**	**Response**
**Competing Interests**<br><br>Use the instructions below to enter a competing interest statement for this submission. On behalf of all authors,	The authors have declared that no competing interests exist.

Powered by Editorial Manager® and ProduXion Manager® from Aries Systems Corporation

disclose any competing interests that could be perceived to bias this work—acknowledging all financial support and any other relevant financial or non-financial competing interests.<br><br>This statement is required for submission and **will appear in the published article** if the submission is accepted. Please make sure it is accurate and that any funding sources listed in your Funding Information later in the submission form are also declared in your Financial Disclosure statement.<br><br>View published research articles from *PLOS ONE* for specific examples.<br><br>**NO authors have competing interests**<br><br>Enter: *The authors have declared that no competing interests exist.*<br><br>**Authors with competing interests**<br><br>Enter competing interest details beginning with this statement:<br><br>*I have read the journal's policy and the authors of this manuscript have the following competing interests: [insert competing interests here]*<br><br>* typeset	
**Financial Disclosure**<br><br>Enter a financial disclosure statement that describes the sources of funding for the work included in this submission. Review the submission guidelines for detailed requirements. View published research articles from *PLOS ONE* for specific examples.<br><br>This statement is required for submission and **will appear in the published article** if the submission is accepted. Please make	Yes

sure it is accurate.<br><br>**Funded studies**<br>Enter a statement with the following details:<br>• Initials of the authors who received each award<br>• Grant numbers awarded to each author<br>• The full name of each funder<br>• URL of each funder website<br>• Did the sponsors or funders play any role in the study design, data collection and analysis, decision to publish, or preparation of the manuscript?<br><br>Did you receive funding for this work?	
Please add funding details.<br>as follow-up to "**Financial Disclosure**<br><br>Enter a financial disclosure statement that describes the sources of funding for the work included in this submission. Review the submission guidelines for detailed requirements. View published research articles from *PLOS ONE* for specific examples.<br><br>This statement is required for submission and **will appear in the published article** if the submission is accepted. Please make sure it is accurate.<br><br>**Funded studies**<br>Enter a statement with the following details:<br>• Initials of the authors who received each award<br>• Grant numbers awarded to each author<br>• The full name of each funder<br>• URL of each funder website<br>• Did the sponsors or funders play any role in the study design, data collection and analysis, decision to publish, or preparation of the manuscript?<br><br>Did you receive funding for this work?"	BH received funding from the French Facility for Global Environment (Grant Terri4Sol). The funder had no role in study design, datacollection and analysis, decision to publish or preparation of the manuscript.<br><br>https://www.ffem.fr/en
Please select the country of your main research funder (please select carefully	FRANCE - FR

as in some cases this is used in fee calculation).<br>as follow-up to "**Financial Disclosure**<br><br>Enter a financial disclosure statement that describes the sources of funding for the work included in this submission. Review the submission guidelines for detailed requirements. View published research articles from *PLOS ONE* for specific examples.<br><br>This statement is required for submission and **will appear in the published article** if the submission is accepted. Please make sure it is accurate.<br><br>**Funded studies**<br>Enter a statement with the following details:<br>• Initials of the authors who received each award<br>• Grant numbers awarded to each author<br>• The full name of each funder<br>• URL of each funder website<br>• Did the sponsors or funders play any role in the study design, data collection and analysis, decision to publish, or preparation of the manuscript?<br><br>Did you receive funding for this work?"	
**Ethics Statement**<br><br>Enter an ethics statement for this submission. This statement is required if the study involved:<br><br>• Human participants<br>• Human specimens or tissue<br>• Vertebrate animals or cephalopods<br>• Vertebrate embryos or tissues<br>• Field research<br><br>Write "N/A" if the submission does not require an ethics statement.<br><br>General guidance is provided below. Consult the submission guidelines for	N/A

Powered by Editorial Manager® and ProduXion Manager® from Aries Systems Corporation

detailed instructions. **Make sure that all information entered here is included in the Methods section of the manuscript.**<br><br>**Format for specific study types**<br><br>**Human Subject Research (involving human participants and/or tissue)**<br>• Give the name of the institutional review board or ethics committee that approved the study<br>• Include the approval number and/or a statement indicating approval of this research<br>• Indicate the form of consent obtained (written/oral) or the reason that consent was not obtained (e.g. the data were analyzed anonymously)<br><br>**Animal Research (involving vertebrate animals, embryos or tissues)**<br>• Provide the name of the Institutional Animal Care and Use Committee (IACUC) or other relevant ethics board that reviewed the study protocol, and indicate whether they approved this research or granted a formal waiver of ethical approval<br>• Include an approval number if one was obtained<br>• If the study involved *non-human primates*, add *additional details* about animal welfare and steps taken to ameliorate suffering<br>• If anesthesia, euthanasia, or any kind of animal sacrifice is part of the study, include briefly which substances and/or methods were applied<br><br>**Field Research**<br><br>Include the following details if this study involves the collection of plant, animal, or other materials from a natural setting:<br>• Field permit number<br>• Name of the institution or relevant body that granted permission	
**Data Availability**<br><br>Before publication, Authors are required to make fully available and without restriction all data underlying their	Dataset is available at Zenodo at https://doi.org/10.5281/zenodo.21505800

findings. Sharing the data underlying your findings increases the visibility, credibility, and impact of your work, while supporting transparency and future scientific discovery. Please see our PLOS Data Policy page for detailed information on this policy.<br><br>A **Data Availability Statement**, detailing where the data can be accessed, is required at first submission. Insert your Data Availability Statement in the box below.<br><br>Please see the data reporting section of our submission guidelines for instructions on what you need to include in your Data Availability Statement. Examples of acceptable Data Availability Statements are included here for reference:<br>1. The minimal data set [and accompanying code, where generated] is available at [repository name] via [DOI hyperlink]<br>2. No data was generated by this study. The following existing data sources were used: [dataset name] from [dataset location] available via [web address or DOI hyperlink].<br>3. For Study Protocols: No datasets were generated or analysed during the current study. All relevant data from this study will be made available upon study completion.<br><br>*PLOS allows rare exemptions to address legal and ethical concerns. If you have legal or ethical restrictions, please use the box below to detail these in full sentences for the Journal team to consider.*<br><br>This statement will be typeset if the manuscript is accepted for publication.	

Manuscript

Click here to access/download;Manuscript;Plos one_paper_revised-black.docx

Regenerating forests around Taï National Park sustain biodiversity despite constrained floristic recovery

Aya Diane Larissa Houphouët [1**], Yao Charles Sangne [1], Abdoulaye Diarrassouba [2], Evans Ehouman[3,4], Bruno Hérault [3,4*]

[1] Université Jean Lorougnon Guédé, Unité de Formation et de Recherche en Environnement, Daloa, Côte d'Ivoire

[2] Office Ivoirien des Parcs et Réserves, Soubré, Côte d'Ivoire

[3] CIRAD, Forêts et Sociétés, F-34398, Montpellier, France

[4] Forêts et Sociétés, Univ Montpellier, CIRAD, Montpellier, France

***Corresponding authors**

*bruno.herault@cirad.fr (BH)

**larissahouphouet@gmail.com (ADLH)

ABSTRACT

Extensive deforestation and disturbance have reshaped forest landscapes in West Africa, including areas surrounding Taï National Park, the largest remaining block of Upper Guinean rainforest. Although many degraded areas are currently undergoing regeneration, the capacity of these secondary forests to recover their biodiversity attributes remains insufficiently assessed within this park. This study quantifies the multidimensional restoration of biodiversity across 125 secondary forest plots and 15 primary forest plots representing 14,731 inventoried individuals in four sectors of the park. Using a Bayesian modelling framework, we estimate recovery trajectories of Shannon diversity, floristic composition, functional traits (wood density, leaf mass per area, seed mass), and conservation-relevant species. The different biodiversity attributes recovered at contrasting rates. Diversity was restored more rapidly (λ = 0.06) than floristic composition (λ = 0.03), and these recovery varied according to environmental variables. Among these, the presence of remnant trees showed the highest median on diversity (0.53 ± 0.61) and floristic composition (0.37 ± 0.17), promoting the rapid recovery of both attributes, followed by prior land use, particularly cocoa farming which also positively influenced the recovery of alpha diversity (λ = 0.03) and floristic composition (λ = 0.02). Regarding functional traits, they displayed contrasting dynamics: specific leaf area and seed mass recovered rapidly along successional gradients, whereas wood density followed a more gradual recovery trajectory. From a conservation perspective, although the proportion of IUCN Red List species remained stable along the successional gradient, old-growth indicator species were significantly more abundant in primary forests. To ensure the long-term conservation of biodiversity, protecting both primary and regenerating forests is essential to preserve the ecological resilience of this park.

Introduction

Tropical forests in West Africa harbour exceptionally high levels of biodiversity and form part of one of the world's 25 biodiversity hotspots [1,2]. Despite their ecological importance, these forests have experienced intense deforestation in recent decades, resulting in substantial biodiversity loss [3]. In Côte d'Ivoire, forest conversion has been primarily driven by agricultural expansion, especially cocoa, coffee, and oil palm alongside logging, charcoal production, infrastructure development, and mining [3–5]. These transformations have led not only to biodiversity decline but also to the degradation of ecosystem services and climate regulation.

Against this backdrop of widespread forest loss, restoring degraded lands has emerged as an essential complementary strategy to the conservation of remaining old-growth forests [6]. Secondary forests now represent a substantial proportion of tropical forest cover [7–9] and may contribute meaningfully to biodiversity conservation [10,11]. However, recovery is complex and does not progress equally across ecological features. Although species diversity often increases relatively rapidly following agricultural abandonment [12,13], floristic composition may take decades to converge toward old-growth conditions [14,15]. Moreover, recovery trajectories are strongly shaped by land-use intensity, soil degradation, remnant trees that enhance seed dispersal, and by the connectivity between neighboring landscapes [16–19].

Importantly, biodiversity recovery cannot be fully understood from taxonomic patterns alone. Beyond changes in species richness and composition, ecological succession entails shifts in species' life-history strategies and functional attributes [20]. Early successional stages are typically dominated by fast-growing pioneer species, whereas late-successional and shade-tolerant species establish progressively as forest structure develops [14,21]. These successional dynamics are reflected in changes in functional traits, which provide mechanistic insight into

ecosystem functioning and resilience [22]. Traits such as wood density, leaf mass per area (LMA), and seed mass are closely linked to growth–mortality trade-offs, resource-use strategies, and regeneration processes [23–26]. Seed mass, in particular, mediates the balance between colonization ability and establishment success [27–29]. Integrating functional composition with taxonomic and compositional analyses therefore provides a more comprehensive framework for evaluating forest recovery.

In West Africa, where remaining old-growth forests are extremely limited and deforestation rates remain among the highest globally, conservation increasingly depends on the capacity of secondary forests to sustain biodiversity. Yet it remains unclear whether regenerating forests can meaningfully substitute for primary forests in maintaining endangered species, functional diversity, and ecosystem processes. Addressing this uncertainty is critical for guiding restoration strategies, landscape planning, and protected-area buffering. These issues are especially critical in landscapes subjected to intense anthropogenic pressure, such as those surrounding Taï National Park in south-western Côte d'Ivoire. Taï National Park constitutes the largest remaining continuous block of Upper Guinean humid rainforest and one of the last extensive remnants of tropical rainforest in West Africa. As a major stronghold of regional biodiversity, it harbours numerous endemic and threatened species. However, episodes of degradation particularly during the 2002-2011 politico-military crises have affected both the park and its periphery. Since 2015, degraded areas have been undergoing natural regeneration [30], creating a mosaic of intact old-growth forest, disturbed old-growth forest, and secondary forests of varying ages. In such a globally significant forest landscape, understanding the extent and pace of biodiversity recovery is essential for informing regional conservation and restoration strategies.

To date, few studies in Upper Guinean forests have jointly quantified taxonomic, functional, compositional, and conservation-relevant recovery using reference-based models. Here, we provide one of the first multidimensional assessments of natural forest regeneration surrounding Taï National Park, integrating community composition, functional traits, biomass, and species conservation status within a unified Bayesian framework. To provide conceptual clarity, we define “recovery” as the degree of convergence of biodiversity attributes in secondary forests toward reference conditions observed in intact old-growth forests. Recovery therefore refers not only to increases in species diversity over time, but also to compositional similarity and functional convergence relative to old-growth benchmarks. This reference-based approach allows us to distinguish between rapid gains in taxonomic diversity and slower or incomplete recovery of compositional and functional attributes. Building on this framework, we analyse biodiversity recovery trajectories in secondary forests surrounding Taï National Park. Specifically, we address the following questions: (i) What are the recovery trajectories of Shannon diversity and floristic composition, and which factors influence them? (ii) How do key functional traits such as wood density, leaf mass per area, and seed mass change along secondary succession, and can they serve as indicators of forest recovery? and (iii) Do secondary forests contribute to the persistence and recovery of conservation-relevant species?

Methods

Study Area

The study was conducted in and around Taï National Park (TNP), located in south-western of Côte d'Ivoire between 5°15′ to 6°07′ N and 7°25′ to 7°54′ W (Fig 1). TNP constitutes the largest remaining continuous block of Upper Guinean humid rainforest and is recognised as a UNESCO World Heritage Site [31,32]. The park covers approximately 5,360 km² and represents one of the last extensive remnants of primary tropical rainforest in West Africa [33] . The region is characterised by a humid tropical climate with two rainy seasons (March to July and September to November) and two relatively drier periods. Mean annual rainfall ranges between 1,700 and 2,200 mm, and mean annual temperature is approximately 25–27 °C. Soils are predominantly ferralitic and vary locally depending on topography and drainage conditions. Vegetation is dominated by evergreen and semi-deciduous moist forest. The park harbours high levels of plant and vertebrate diversity, including numerous endemic and threatened species characteristic of the Upper Guinean forest block [34,35]. Surrounding landscapes have been extensively transformed by agricultural expansion, particularly cocoa cultivation, as well as logging and artisanal gold mining.

During the post-electoral crises of 2000 and 2010, reduced surveillance led to forest encroachment and degradation in parts of the park and its periphery. Since 2015, restoration and natural regeneration processes have been ongoing in previously degraded areas [36]. As a result, the current landscape forms a mosaic of intact old-growth forest, disturbed old-growth forest, and secondary forests of varying ages originating from former agricultural lands or mining sites.

Fig. 1. Spatial distribution of sampling plots across Taï National Park and surrounding landscapes in south-western Côte d'Ivoire. Sampling plots represent a chronosequence of secondary forests (9–26 years since abandonment; n = 125) and reference intact old-growth forests (n = 15). Plots are distributed within and around park boundaries. The map also shows Buyo Lake, major waterways, and nearby cities to provide geographic context. The inset highlights the northern sampling cluster near Buyo Lake, where forest fragmentation has created a mosaic of secondary and old-growth stands. Printed under a CC BY license, with permission from OIPR, original copyright [2026].

Vegetation Data

A total of 140 vegetation plots were established in and around Taï National Park. Of these, 125 plots were located in secondary forests and 15 plots in intact old-growth forests, which served as reference sites. Each plot measured 25 × 25 m (0.0625 ha). Secondary forests represented a chronosequence ranging from 9 to 26 years since abandonment. Forest age was determined based on information from local communities, park management records, and field evidence of past land use. Following the post-electoral crisis period, agricultural activities inside the park were discontinued and cocoa plantations were progressively removed as part of restoration and management interventions. Subsequent natural succession gave rise to stands of varying ages, forming the basis of the chronosequence analysed in this study. Within each 25 × 25 m plot, all woody stems with a diameter at breast height (DBH) ≥ 2.5 cm were inventoried. DBH was measured at 1.3 m above ground level. For multi-stemmed individuals, each stem meeting the diameter threshold was recorded separately. Lianas were excluded from the inventory. Species were identified in the field by experienced botanists. When identification was uncertain, specimens were examined using the regional floras of Aké-Assi [37] and Hutchinson & Dalziel [38] to ensure accurate determination. Although voucher specimens were not systematically deposited in a public herbarium, taxonomic verification procedures were applied to ensure consistency and reliability across plots. Nomenclature was standardised according to the

Angiosperm Phylogeny Group IV (APG IV) classification system, and species names were harmonised to avoid synonymy or spelling inconsistencies within the dataset.

All original data used in this study will be publicly available and can be accessed from Zenodo upon acceptance. Permissions for this study were granted by the Office Ivoirien des Parcs et Réserves (OIPR), the governmental body responsible for managing Taï National Park, Côte d'Ivoire.

Functional Trait Data

We analysed three functional traits : wood density (WD), leaf mass per area (LMA), and seed mass. These traits were selected because of their established links to growth–mortality trade-offs, resource-use strategies, regeneration processes, and successional dynamics in tropical forests [23,24] .

Wood density values were retrieved using the R package *BIOMASS* [39] through the function *getWoodDensity()*, which assigns species-level values when available and substitutes genus-level averages when species-specific data are missing. This procedure ensured complete coverage of wood density values across all species in the dataset. Seed mass and LMA values were obtained primarily from the TRY global plant trait database [40], including regional datasets integrated into TRY [41] . Seed mass data were additionally compiled from the SER-SID database (ser-sid.org). For species lacking values in these databases, seed mass measurements were supplemented using published data from [42] .

When multiple trait values were available for a given species across datasets, the median value was used to reduce the influence of extreme observations. Species-level trait values were then assigned to all corresponding individuals in the vegetation dataset. Trait coverage varied among

traits. Wood density values were available for 100% of species, seed mass for approximately 49% of individuals, and LMA for 56% of individuals.

Environmental variables

To account for ecological and anthropogenic drivers of recovery, we recorded a set of environmental and disturbance-related variables expected to influence regeneration processes (Table 1).

- Large and medium-sized mammals were included because they play a key role in seed dispersal and recruitment dynamics in tropical forests. Their presence can enhance colonization by late-successional species and influence community composition. Mammal occurrence was assessed through indirect field signs of species known to function as seed dispersers or large herbivores.
- Remnant trees were quantified as indicators of biological legacies from pre-disturbance forests. Such trees can provide local seed sources, structural complexity, and perching sites for frugivores, thereby potentially accelerating succession.
- Soil hydromorphy was recorded as a proxy for edaphic constraints on species establishment and growth, as waterlogged conditions can limit recruitment of certain functional groups.
- Island configuration was included to capture landscape isolation effects, which may reduce propagule influx and alter recovery trajectories.
- Signs of ongoing human activities were documented to account for continued disturbance pressure that may slow or redirect successional dynamics.

Together, these variables capture biotic, abiotic, and anthropogenic drivers of succession and were incorporated as covariates in subsequent models of biodiversity recovery.

Table 1. Plot-level environmental variables used to assess biotic legacies, edaphic constraints, landscape isolation, and ongoing anthropogenic activities potentially influencing biodiversity recovery in secondary forests surrounding Taï National Park, Côte d'Ivoire.

**Variables**	**Description**	**Values**
Mammal	Number of large/medium-sized mammal species detected through indirect signs	0-3
Hydromorphy	Presence of hydromorphic soil features in surface horizons	0/1
Island	Plot located in isolated island	0/1
Remnants	Density of isolated old-growth trees persisting after land conversion	trees.ha^{-1}
Activities	Number of disturbance types observed (poaching, agriculture,mining)	0-3

Conservation-relevant species

To evaluate the contribution of secondary forests to biodiversity conservation, we considered two categories of conservation-relevant species: (i) old-growth indicator species and (ii) species listed under threatened categories of the IUCN Red List.

Indicator species characteristic of old-growth forests were identified using the method of [43] , implemented in the R package *indicspecies*. We compared two groups of plots: intact old-growth forests and secondary forests younger than 20 years. The indicator value (IndVal) combines measures of specificity and fidelity to a given habitat type, allowing the identification of species strongly associated with old-growth conditions. Species were considered significant old-growth indicators when their indicator value was associated with a permutation test P-value < 0.002. A stringent significance threshold was applied to reduce the risk of false positives and

to ensure robust identification of species reliably associated with old-growth forest conditions. For each plot, we subsequently quantified the occurrence and abundance of these indicator species.

For all inventoried species, conservation status was assessed using the IUCN Red List of Threatened Species (https://www.iucnredlist.org/). Species classified as Vulnerable (VU) or Endangered (EN) were considered conservation-relevant for this analysis. The presence and abundance of these species were quantified across forest types to evaluate their persistence and potential recovery during secondary succession.

Biodiversity metrics

Diversity was quantified using Shannon diversity, expressed as the Hill number of order 1 ($q = 1$). Diversity of order 1 corresponds to the exponential of Shannon entropy and represents the effective number of species in a community. Estimates were computed using the R package *entropart* [44]. Sample coverage was first estimated for each plot using the function *coverage()*. As coverage was consistently above 0.90 across plots, diversity was estimated at a standardized coverage level of 0.90 to ensure comparability among plots. This coverage-based standardization minimizes bias due to incomplete sampling while avoiding distortions caused by unequal sampling effort.

Composition reflects both species identity and their relative abundances within each plot. To quantify compositional similarity between secondary forests (SF) and intact old-growth forests (OGF), we calculated the abundance-based Chao–Jaccard similarity index for all possible SF–OGF plot pairs. This index accounts for unseen shared species and corrects for undersampling, thereby providing a robust estimate of compositional similarity. To define the reference state, similarities among old-growth plots (OGF–OGF) were also calculated. These within-OGF

similarities represent the natural variability of intact forests and define the benchmark toward which secondary forests are expected to converge during succession [15]. Although the 15 reference plots are modest relative to the park's total area, they were distributed across four sectors of the park to capture part of its spatial heterogeneity, and their pairwise similarity was used to explicitly characterise — rather than assume — the natural variability of old-growth forest composition used as the recovery benchmark.

Modeling Strategies

All models were built within a Bayesian framework. A Bayesian approach was chosen because of its flexibility in handling complex hierarchical structures, its ability to accommodate different likelihood distributions across response variables, and its explicit quantification of uncertainty through posterior distributions. A log-normal likelihood was used because all response variables considered (diversity, composition, and functional traits) are strictly defined on the positive real line ($\mathbb{R}^+$) and exhibited right-skewed distributions [45]. Log-normal likelihoods were implemented in Stan using its standard parameterization, with mean parameters defined on the original scale and passed to the distribution through the log link. Models were implemented in Stan and fitted using Hamiltonian Monte Carlo (HMC) sampling for parameter estimation [46].

Q1- Biodiversity Recovery Trajectories

To estimate recovery trajectories and intrinsic recovery rates, we applied the exponential recovery model developed by [47]. This model assumes that the instantaneous recovery rate of an ecosystem attribute is proportional to the distance between its current state and its

asymptotic value [45]. Such a formulation captures saturating recovery dynamics commonly observed during secondary succession.

For secondary forests, the likelihood model for a biodiversity attribute X (diversity or composition) in plot *p* at time *t* since abandonment was specified as:

$$X_{p,t} \sim LN\left(\theta_0^X + (\ \theta_{+\infty}^X - \theta_0^X) \,.\left(1 - e^{-\lambda^X t_p}\right), \sigma_X^2\right) \qquad (1)$$

where:

- $X_{p,t}$ is the observed value of attribute *X* in plot *p* at time *t*,
- θ_0^X represents the initial value of *X* at the beginning of the recovery process,
- $\theta_{+\infty}^X$ is the asymptotic value of *X*, corresponding to the long-term equilibrium conditions,
- λ^X is the intrinsic recovery rate controlling the speed of convergence toward the asymptote,
- σ_X^2 represents the residual variance among secondary forest plots.

For intact old-growth forests, time since regeneration is not defined and attribute values are assumed to represent the asymptotic state. The likelihood was therefore specified as:

$$X_{p,t} \sim LN(\theta_{+\infty}^X, \sigma_X^2) \qquad (2)$$

This formulation allows old-growth forests to directly inform the asymptotic parameter $\theta_{+\infty}^X$. To account for anthropogenic disturbance effects in old-growth forests, asymptotic values were adjusted by including a disturbance effect term:

$$X_{p,t} \sim LN(\theta_{+\infty}^X + \theta_{dist}^X, \sigma_X^2) \qquad (3)$$

where θ_{dist}^X represents the effect of disturbance intensity on the asymptotic value of attribute X.

To evaluate how environmental and disturbance-related variables influenced recovery dynamics, covariates (Table 1) were incorporated into the recovery rate parameter λ_p^X. Because recovery rates are strictly positive, we modelled them using an exponential link function:

$$\lambda_p^X = \lambda_{land}^X \times e^{\left(\sum_{j=1}^{J} \beta_{COVj}^X . COV_{j,p}\right)} \quad (4)$$

where:

- λ_{land}^X is the baseline recovery rate associated with previous land use,
- β_{COVj}^X represents the effect of covariate *j* on recovery rate,
- $COV_{j,p}$ is the standardized value of covariate *j* for plot *p*.

All continuous covariates were standardized prior to analysis to facilitate parameter interpretation and comparison of effect sizes.

Q2 - Functional trait dynamics

To analyse how functional traits change during succession, we used an individual-level Bayesian model linking trait values to both stem diameter *DBH* and forest age *t*. This approach captures shifts in trait distributions along the tree size gradient during recovery, consistent with the idea that secondary forests progressively approach old-growth trait–size relationships as forests mature [47]. Because trait values were strictly positive and right-skewed, they were modelled using a log-normal likelihood. For each tree *n* in secondary forests, trait values *Y* (wood density, leaf mass per area, or seed mass) were modelled as:

$$Y_{n,p} \sim LN\left(\left(\alpha_Y + \beta_Y . DBH_n\right) . \left(1 - e^{-\lambda^Y t_p}\right), \sigma_y^2\right) \quad (5)$$

where DBH_n is the diameter of individual n, t_p is time since abandonment for the plot p where the individual occurs, α_Y is the baseline value of the trait when the diameter effect is null, β_Y quantifies the effect of DBH on the expected trait value, λ^Y is the convergence rate toward the asymptotic state, and σ_y^2 is the residual variance. Under this formulation, the expected trait–size relationship converges toward the old-growth reference function $(\alpha_Y + \beta_Y . DBH_n)$ as forest age increases, thereby allowing trait distributions to progressively align with old-growth conditions along the DBH gradient.

For intact old-growth forests, trait values were assumed to reflect the asymptotic (reference) trait–size relationship:

$$Y_n \sim LN\left((\alpha_Y + \beta_Y . DBH_n), \sigma_y^2\right) \quad (6)$$

To account for possible anthropogenic disturbance shifts away from intact reference conditions, we included an additive disturbance effect on the expected trait–size relationship:

$$Y_n \sim LN\left(\theta_Y . (\alpha_Y + \beta_Y . DBH_n), \sigma_y^2\right) \quad (7)$$

where θ_Y represents the effect of disturbance intensity on the expected value of trait Y (i.e., $\theta_Y > 1$ indicates higher expected trait values under disturbance, whereas $\theta_Y < 1$ indicates lower expected trait values).

Q3 - Conservation-relevant species

To assess whether conservation-relevant species are maintained or recovering during succession, we calculated, for each plot, the proportion of individuals belonging to (i) old-growth indicator species and (ii) IUCN-listed species (Vulnerable or Endangered). Secondary forests were grouped into two age classes (≤ 11 years and > 11 years since abandonment) and compared with intact old-growth forests. This comparison allowed us to evaluate whether the

relative representation of conservation-relevant species increases with forest age and approaches old-growth conditions.

Results

Across all forest types, we recorded 14,731 woody individuals (DBH ≥ 2.5 cm), corresponding to 174 species distributed among 138 genera and 48 families.

Biodiversity recovery trajectories

Shannon diversity and floristic composition did not recover at the same rate (Fig 2, Table 2). Shannon diversity increased rapidly during succession, reaching approximately 75% of its asymptotic value within the first 40 years. The intrinsic recovery rate was $\lambda = 0.06$ (90% CI: 0.02–0.17). Diversity started at an estimated 8.94 effective species (θ_0) and converged toward an asymptotic value of 20.39 in old-growth forests ($\theta_{+\infty}$). In contrast, floristic composition recovered more slowly ($\lambda = 0.03$; 90% CI: 0.00–0.16). Because λ is constrained to be strictly positive by the model's exponential link function, this interval does not literally include zero; however, its lower bound is very close to zero, indicating that a near-negligible, practically stagnant recovery rate cannot be excluded. On average, secondary forests required over 40 years to approach half of the asymptotic similarity observed in old-growth forests (Fig 2). Composition increased from an initial value of 0.19 to an asymptote of 0.72 (Table 2). Previous land use influenced recovery rates. Recovery was faster in former cocoa farms than in former mining sites for diversity, whereas land-use effects on compositional recovery were negligible (Table 2).

Fig. 2. Modelled recovery trajectories of Shannon diversity and floristic composition in secondary tropical forests of West Africa (Taï National Park, Côte d'Ivoire). Lines represent posterior mean recovery trajectories estimated from the Bayesian exponential recovery model; shaded areas indicate 95% credible intervals. Solid lines correspond to the temporal range robustly informed by the underlying 9–26 year chronosequence (up to ~40 years since disturbance); dashed lines

represent the model's extrapolation beyond this range, up to 200 years, and should be interpreted with caution given the increasing uncertainty. Recovery is expressed as the percentage of convergence toward old-growth reference conditions, with 100% corresponding to the asymptotic state observed in intact West African old-growth forests.

Table 2. Posterior summaries of the recovery model parameters for diversity and composition in secondary forests of West Africa.

**Parameters**	**Diversity**			**Composition**		
	Median	5th	95th	Median	5th	95th
θ_0	8.94	3.83	17.96	0.19	0.02	0.71
$\theta_{+\infty}$	20.39	16.15	24.90	0.72	0.52	0.97
λ	0.06	0.02	0.17	0.03	0.00	0.16
λ_{Cocoa}	0.03	0.01	0.08	0.01	0.00	0.03
λ_{Mining}	0.02	0.01	0.06	0.01	0.00	0.02
λ_{others}	0.08	0.02	0.18	0.02	0.00	0.06
θ_{dist}	0.51	-3.88	4.44	-0.27	-0.44	-0.17
σ	0.44	0.40	0.50	1.74	1.68	1.81

Posterior medians and 90% credible intervals (5th–95th percentiles) of the exponential recovery model parameters for Shannon diversity (Hill number of order 1) and floristic composition (abundance-based Chao–Jaccard similarity). θ_0 represents the initial value of the recovery trajectory; $\theta_{+\infty}$ the asymptotic value corresponding to old-growth reference conditions; λ the intrinsic recovery rates ; θ_{dist} the effect of past disturbance on the asymptotic value; and σ the residual variance of the log-normal likelihood. Posterior estimates of environmental covariate effects (β parameters) are not shown here and are presented in Fig. 3.

Environmental covariates had contrasting effects on recovery rates (Fig 3). Remnant trees showed positive effects on both diversity and composition. Human activities and island configuration were associated with slower recovery of both attributes. Mammal presence increased compositional recovery but was negatively associated with diversity recovery. Soil hydromorphy and disturbance exhibited variable effects, with wide credible intervals for diversity but negative effects on compositional recovery.

Fig.3. Environmental drivers of diversity and composition recovery rates. Points represent posterior mean effects of standardized covariates on the intrinsic recovery rate (λ) for diversity (x-axis) and composition (y-axis). Error bars indicate 95% credible intervals. Vertical and horizontal dashed lines represent zero effect. Positive values indicate an increase in recovery rate, whereas negative values indicate slower recovery. Covariates include mammal presence, remnant tree density, soil hydromorphy, island location, human activities, and disturbance intensity.

Functional Trait Dynamics

Trait values varied jointly with stem diameter and forest age, with distinct convergence rates among traits (Table 3).

Wood density showed a gradual increase with forest age and remained structured by stem diameter (Fig 4). Larger individuals exhibited slightly lower wood density values than smaller individuals, consistent with the negative size effect ($\beta = -0.17$). Convergence toward old-growth conditions was slow ($\lambda = 0.15$). The disturbance parameter ($\theta = 0.97$) indicated slightly lower expected values under disturbance relative to intact old-growth forests.

LMA was strongly influenced by stem diameter, with larger trees displaying higher values ($\beta = 4.63$). In contrast to wood density, LMA converged rapidly toward old-growth reference

conditions (λ = 1.40). Predicted values exceeded 60 $g.m^{-2}$ within the first decade of succession across most of the DBH gradient (Fig 4). The disturbance effect was minimal (θ = 0.98).

Seed mass increased with stem diameter (β = 1.26) and exhibited rapid convergence dynamics (λ = 1.21). Predicted seed mass values for larger individuals exceeded 25 mg within the first 10 years of succession and remained stable thereafter (Fig 4). The disturbance parameter was close to unity (θ = 0.98; 90% CI: 0.95–0.99), indicating limited deviation from intact old-growth reference values.

Table 3. Posterior summaries of the recovery model parameters for functional traits in secondary forests of West Africa.

**Parameters**	**Seed mass**			**Wood density**			**Leaf mass per area**		
	Median	5th	95th	Median	5th	95th	Median	5th	95th
α	20.43	19.89	21.01	0.61	0.61	0.62	60.12	59.63	60.60
β	1.26	0.41	2.11	-0.17	-0.18	-0.16	4.63	4.63	5.46
λ	1.21	0.72	2.25	0.15	0.15	0.16	1.4	0.9	2.47
θ	0.98	0.95	0.99	0.97	0.96	0.99	0.98	0.99	0.99
σ	2.85	2.81	2.89	0.22	0.22	0.23	3.62	3.5	3.69

Posterior medians and 90% credible intervals (5th–95th percentiles) of the individual-level exponential convergence model parameters for seed mass, wood density, and leaf mass per area. α represents the expected trait value when DBH = 0; β quantifies the effect of stem diameter on trait values; λ is the intrinsic convergence rate toward old-growth reference conditions; θ represents the multiplicative effect of disturbance on trait values in old-growth forests; and σ is the residual variance of the log-normal likelihood.

Fig. 4. Modelled trajectories of functional trait distributions along the DBH and successional age gradients in secondary forests of West Africa. Heatmaps represent posterior mean predicted values of wood density ($g.cm^{-3}$), leaf mass per area (LMA; $g.m^{-2}$), and seed mass (mg)

as functions of stem diameter (DBH, m; x-axis) and time since abandonment (years; y-axis). Color gradients indicate expected trait values derived from the individual-level convergence model. Predictions illustrate progressive alignment of trait–size relationships toward old-growth reference conditions as forest age increases.

Conservation-relevant species

Among the 174 recorded species, 16 (9.2%) were classified as either Endangered (EN) or Vulnerable (VU) according to the IUCN Red List (Appendix 1), and 44 species (25.3%) were identified as old-growth indicator species based on the IndVal analysis (Appendix 2).

IUCN-listed species were present across all forest stages. Their proportion remained relatively stable among young secondary forests (≤ 11 years), older secondary forests (> 11 years), and intact old-growth forests, with values ranging between 8% and 9% of individuals (Fig 5).

In contrast, the proportion of old-growth indicator species increased with forest age. Indicator species represented approximately 3–5% of individuals in young secondary forests, 4–6% in intermediate secondary forests, and 7–11% in old-growth forests (Fig 5).

Fig. 5. Proportion of conservation-relevant species across successional stages in West African tropical forests. Boxplots show the percentage of individuals belonging to old-growth indicator species (left panel) and IUCN-listed species categorized as Vulnerable or Endangered (right panel) across three forest categories: secondary forests ≤ 11 years since abandonment, secondary forests > 11 years, and intact old-growth forests. Boxes represent interquartile ranges, horizontal lines indicate medians, and whiskers show the range of observed values

Discussion

In the last extensive block of Upper Guinean rainforest in West Africa, our results show that secondary forests surrounding Taï National Park retain substantial ecological potential. Although biodiversity dimensions recover at different rates, species diversity rebounds relatively quickly, functional attributes re-establish within decades, and conservation-relevant species persist across successional stages. These findings provide encouraging evidence that, when protected from further disturbance, naturally regenerating forests in this region can contribute meaningfully to biodiversity conservation and landscape resilience.

Rapid diversity recovery masks slower and context-dependent compositional convergence

The rapid recovery of diversity likely reflects the resilience of secondary forests and the progressive accumulation of species following land abandonment [48]. Similar patterns have been reported in Neotropical forests, where up to 90% of species richness can recover within two decades [48–50]. In contrast, floristic composition depends on the return of late-successional species, many of which are dispersal-limited and characterized by larger seed mass [51,52]. As a result, compositional convergence may require several decades to centuries. Comparable trajectories have been observed in the Congo Basin [53], the Neotropics [54], and Southeast Asia [52], reinforcing the generality of this asynchronous recovery pattern.

Importantly, compositional recovery was not only slower but also highly variable, reflecting strong context dependence. It should be noted that the lower bound of the credible interval for the compositional recovery rate is very close to zero, meaning our data cannot exclude a near-negligible recovery rate that would be practically equivalent to stagnation over the sampled chronosequence (9–26 years); this uncertainty highlights the need for longer-term monitoring

to distinguish slow recovery from an effective stall. Previous land use played a central role. Abandoned cocoa plantations often retain partial soil fertility [55] and remnant trees [56,57], which facilitate regeneration and canopy re-establishment [58,59]. In contrast, artisanal gold mining removes topsoil, disrupts seed banks, alters soil structure, and may lead to heavy metal accumulation, reducing microbial activity and long-term fertility [16,60]. Such disturbances strongly constrain recolonization by forest specialists and slow compositional convergence [41].

Environmental factors further modulated recovery trajectories. Remnant trees emerged as a key driver accelerating both diversity and compositional recovery. These trees create favorable microhabitats, increase structural heterogeneity, and act as local seed sources [55,61]. They also attract seed dispersers such as birds and bats, facilitating the recruitment of late-successional species [62,63]. Similar patterns have been documented elsewhere, where species richness and compositional similarity to primary forests increase around remnant trees after two decades of succession [64,65]. Conversely, human activities and forest isolation slowed recovery. Hunting and logging associated with gold mining reduce populations of large seed dispersers, thereby limiting recruitment of large-seeded forest species [66,67]. Forests on islands created a fragmentation experience that reduced propagule influx and strong edge effects, favoring pioneer and generalist species [68–70]. In line with island biogeography theory [71], limited connectivity constrains long-term compositional recovery. Hydromorphic soils also exerted contrasting effects, promoting herbaceous dominance (e.g., Marantaceae) that can inhibit woody recruitment and slow structural and compositional reassembly [59,72–74].

Together, these results show that while diversity rebounds relatively quickly in regenerating forests of West Africa, floristic composition follows a slower and highly context-dependent

trajectory. Around Taï National Park, this asynchrony underscores the importance of maintaining landscape connectivity, remnant trees, and effective protection from continued disturbance to support long-term compositional recovery.

Rapid reassembly of leaf and regeneration traits contrasts with slow structural maturation

Functional traits recovered at markedly different rates, revealing distinct ecological dimensions of succession in the forests surrounding Taï National Park. In intact old-growth forests, trait distributions reflect conservative resource-use strategies adapted to closed-canopy conditions, where light is limiting and competition for water and nutrients is intense [75]. Dominant species tend to be shade tolerant and invest in durable tissues, including dense wood and structurally robust leaves.

In contrast, secondary forests initially host species that prioritize rapid growth over structural investment. The lower wood density observed in younger forests likely reflects the dominance of pioneer species that colonized soon after abandonment. These heliophilous species capitalize on high light availability created by disturbance, investing in fast height growth rather than dense, mechanically resistant wood. As succession proceeds and canopy closure increases, later-arriving species face reduced light and heightened resource competition [76]. These species typically allocate more biomass to dense wood, enhancing mechanical stability and resistance to disturbance [77]. Such ontogenetic and successional shifts in species composition likely explain the slow convergence of wood density, consistent with broader evidence that structural maturation in tropical forests requires several decades [21,78].

In contrast, leaf mass per area (LMA) reassembled rapidly. LMA increased with both forest age and stem diameter, reflecting adjustments to vertical light gradients and hydraulic constraints. Leaves positioned higher in the canopy experience greater irradiance and wind

exposure, promoting thicker and denser leaf construction [79,80]. Increased hydraulic path length in taller individuals further constrains water transport to the canopy, favoring structural reinforcement of leaves. Smaller trees and understory individuals, by contrast, operate under lower light intensity and shorter hydraulic distances, allowing thinner and lighter leaves. The rapid convergence of LMA suggests that canopy stratification and associated microclimatic gradients re-establish relatively quickly during succession. Because LMA is assigned as a fixed species-level value to every individual of a given species, rather than measured directly on individual trees, our data cannot capture in situ or ontogenetic adjustment of leaf traits within individuals. The observed convergence in LMA therefore necessarily reflects progressive species turnover toward taxa with characteristically higher LMA, not physiological or plastic adaptation of existing trees. In addition, LMA values were available for only 57% of individuals, and species lacking a trait record were excluded from this analysis; while we have no indication of systematic bias in which species were missing, this limited coverage should be kept in mind when assessing the generality of the LMA convergence pattern.

Seed mass dynamics reflect a distinct regeneration axis. Pioneer species typically produce numerous small and light seeds that disperse effectively into disturbed environments [29,81]. Although these seeds contain limited reserves, they benefit from high-light conditions and rapid germination. In contrast, shade-tolerant species produce fewer but larger seeds rich in reserves, enhancing seedling survival under low-light and competitive understory conditions [82–84]. Small-seeded species compensate for lower individual survival through high fecundity and persistence in soil seed banks [81,85]. The relatively rapid stabilization of seed mass distributions indicates that regeneration strategies diversify early in succession, even before full structural maturation of the forest.

Together, these patterns show that while leaf-level adjustments and regeneration strategies reassemble within decades, structural attributes associated with long-lived woody tissues recover more slowly. Around Taï National Park, this decoupling between rapid functional adjustment and delayed structural maturation highlights that forest recovery involves multiple ecological dimensions operating on different temporal scales.

Naturally regenerating forests contribute meaningfully to conservation

The presence of IUCN-listed species across all forest stages indicates that naturally regenerating forests around Taï National Park maintain conservation value throughout succession. The proportion of Endangered and Vulnerable species remained relatively stable across young secondary, older secondary, and old-growth forests, suggesting that early successional habitats contribute to sustaining threatened taxa. Similar complementary roles of secondary forests have been reported in other tropical landscapes [86–88].

In contrast, old-growth indicator species were markedly more abundant in intact forests than in secondary stands (Fig. 5). Although indicator species were present in both young and older secondary forests likely reflecting the persistence of remnant trees [89], their proportion remained substantially lower than in old-growth forests. This pattern suggests that while secondary forests support elements of primary forest composition, full recovery of old-growth identity requires longer time spans and sustained protection. Comparable trends have been documented in West Africa, where indicator species progressively increase but remain less abundant in secondary stands than in primary forests [73].

Together, these findings highlight the complementary but not substitutive role of secondary forests in the Upper Guinean region. While they do not yet replicate the full compositional integrity of old-growth forests, their capacity to maintain threatened species and retain primary-forest elements underscores their importance for biodiversity conservation in West Africa.

Conclusion

Following episodes of intense degradation during the post-electoral crises, the forests surrounding Taï National Park have undergone a decade of natural regeneration. Our results show that this regeneration is not merely structural [59,90] but ecologically meaningful. Secondary forests recover species diversity rapidly, reassemble key functional attributes within decades, and retain threatened taxa across successional stages. Although full compositional convergence toward old-growth forests remains slower and strongly context-dependent, the progressive return of primary-forest elements demonstrates substantial ecological resilience in the last major block of Upper Guinean rainforest. In a region where deforestation has drastically reduced forest cover, these findings provide concrete evidence that protecting regenerating forests around Taï is not a secondary objective but a central pillar of long-term conservation. Safeguarding both intact old-growth stands and naturally recovering forests is essential to maintain biodiversity, ecological functions, and landscape connectivity in West Africa.

Acknowledgments

The authors wish to thank the management of Taï National Park for their logistical and financial support, as well as the forest rangers whose assistance greatly facilitated the data collection phase

References

1. Myers N, Mittermeier RA, Mittermeier CG, da Fonseca GAB, Kent J. Biodiversity hotspots for conservation priorities. Nature. févr 2000;403(6772):853-8. doi:10.1038/35002501

2. Sechrest W, Brooks TM, da Fonseca GAB, Konstant WR, Mittermeier RA, Purvis A, et al. Hotspots and the conservation of evolutionary history. Proc Natl Acad Sci U S A. 19 févr 2002;99(4):2067-71. doi:10.1073/pnas.251680798 PubMed PMID: 11854502; PubMed Central PMCID: PMC122320.

3. Barima YSS, Kouakou ATM, Bamba I, Sangne YC, Godron M, Andrieu J, et al. Cocoa crops are destroying the forest reserves of the classified forest of Haut-Sassandra (Ivory Coast). Glob Ecol Conserv. 1 oct 2016;8:85-98. doi:10.1016/j.gecco.2016.08.009

4. Ruf F, Koné D. Climate change, cocoa migrations and deforestation in West Africa: What does the past tell us about the future. Sustain Sci. 2015;10:101-11. doi:10.1007/s11625-014-0282-4

5. Sanial E, Ruf F, Louppe D, Mietton M, Hérault B. Local farmers shape ecosystem service provisioning in West African cocoa agroforests. Agrofor Syst. mars 2023;97(3):401-14. doi:10.1007/s10457-021-00723-6

6. Depecker J, Asimonyio JA, Miteho R, Hatangi Y, Kambale JL, Verleysen L, et al. The association between rainforest disturbance and recovery, tree community composition, and community traits in the Yangambi area in the Democratic Republic of the Congo. J Trop Ecol. nov 2022;38(6):426-36. doi:10.1017/S0266467422000347

7. Chazdon RL. Tropical forest recovery: legacies of human impact and natural disturbances. Perspect Plant Ecol Evol Syst. 1 janv 2003;6(1):51-71. doi:10.1078/1433-8319-00042

8. Chazdon RL. Second Growth: The Promise of Tropical Forest Regeneration in an Age of Deforestation. In: Second Growth [Internet]. University of Chicago Press; 2014 [cité 3 mai 2025]. Disponible sur: https://www.degruyterbrill.com/document/doi/10.7208/9780226118109/html

9. FAO. Évaluation des ressources forestières mondiales [Rapport final]. Rome; 2020. p. 16 p.

10. Brancalion PHS, Niamir A, Broadbent E, Crouzeilles R, Barros FSM, Almeyda Zambrano AM, et al. Global restoration opportunities in tropical rainforest landscapes. Sci Adv. 3 juill 2019;5(7):eaav3223. doi:10.1126/sciadv.aav3223

11. Teixeira HM, Cardoso IM, Bianchi FJJA, da Cruz Silva A, Jamme D, Peña-Claros M. Linking vegetation and soil functions during secondary forest succession in the Atlantic forest. For Ecol Manag. 1 févr 2020;457:117696. doi:10.1016/j.foreco.2019.117696

12. Aide TM, Clark ML, Grau HR, López-Carr D, Levy MA, Redo D, et al. Deforestation and Reforestation of Latin America and the Caribbean (2001–2010). Biotropica. 2013;45(2):262-71. doi:10.1111/j.1744-7429.2012.00908.x

13. Arroyo-Rodríguez V, Melo FPL, Martínez-Ramos M, Bongers F, Chazdon RL, Meave JA, et al. Multiple successional pathways in human-modified tropical landscapes: new insights from forest succession, forest fragmentation and landscape ecology research: Multiple successional pathways. Biol Rev. févr 2017;92(1):326-40. doi:10.1111/brv.12231

14. Chazdon RL, Finegan B, Capers RS, Salgado-Negret B, Casanoves F, Boukili V, et al. Composition and Dynamics of Functional Groups of Trees During Tropical Forest Succession in Northeastern Costa Rica. Biotropica. 2010;42(1):31-40. doi:10.1111/j.1744-7429.2009.00566.x

15. Poorter L, Craven D, Jakovac CC, van der Sande MT, Amissah L, Bongers F, et al. Multidimensional tropical forest recovery. Science. déc 2021;374(6573):1370-6. doi:10.1126/science.abh3629

16. Peterson GD, Heemskerk M. Deforestation and forest regeneration following small-scale gold mining in the Amazon: the case of Suriname. Environ Conserv. juin 2001;28(2):117-26. doi:10.1017/S0376892901000121

17. Derroire G, Tigabu M, Odén PC, Healey JR. The Effects of Established Trees on Woody Regeneration during Secondary Succession in Tropical Dry Forests. Biotropica. mai 2016;48(3):290-300. doi:10.1111/btp.12287

18. Arroyo-Rodríguez V, PL Melo F, Martínez-Ramos M, Bongers F, L. Chazdon R, A. Meave J, et al. Multiple successional pathways in human-modified tropical landscapes: new insights from forest succession, forest fragmentation and landscape ecology research. Biol Rev [Internet]. 5 nov 2015 [cité 29 avr 2025];92(1):326-40. Disponible sur: https://onlinelibrary.wiley.com/doi/10.1111/brv.12231

19. Amani BHK, N’Guessan AE, Derroire G, N’dja JK, Elogne AGM, Traoré K, et al. The potential of secondary forests to restore biodiversity of the lost forests in semi-deciduous West Africa. Biol Conserv. juill 2021;259. doi:10.1016/j.biocon.2021.109154

20. Wright SJ, Kitajima K, Kraft NJB, Reich PB, Wright IJ, Bunker DE, et al. Functional traits and the growth–mortality trade-off in tropical trees. Ecology. 2010;91(12):3664-74. doi:10.1890/09-2335.1

21. Poorter L, Rozendaal DMA, Bongers F, de Almeida-Cortez JS, Almeyda Zambrano AM, Álvarez FS, et al. Wet and dry tropical forests show opposite successional pathways in wood density but converge over time. Nat Ecol Evol. juin 2019;3(6):928-34. doi:10.1038/s41559-019-0882-6

22. Lavorel S, Garnier E. Predicting changes in community composition and ecosystem functioning from plant traits: revisiting the Holy Grail. Funct Ecol. 2002;16(5):545-56. doi:10.1046/j.1365-2435.2002.00664.x

23. Chave J, Coomes D, Jansen S, Lewis SL, Swenson NG, Zanne AE. Towards a worldwide wood economics spectrum. Ecol Lett. 1 avr 2009;12(4):351-66. doi:10.1111/j.1461-0248.2009.01285.x

24. Poorter L. The Relationships of Wood-, Gas- and Water Fractions of Tree Stems to Performance and Life History Variation in Tropical Trees. Ann Bot. sept 2008;102(3):367-75. doi:10.1093/aob/mcn103

25. Schmitt S, Maréchaux I, Chave J, Fischer FJ, Piponiot C, Traissac S, et al. Functional diversity improves tropical forest resilience: Insights from a long-term virtual experiment. Gomez-Aparicio L, éditeur. J Ecol. mai 2020;108(3):831-43. doi:10.1111/1365-2745.13320

26. Herault B, Ouallet J, Blanc L, Wagner F, Baraloto C. Growth responses of neotropical trees to logging gaps. J Appl Ecol. 2010;47(4):821-31. doi:10.1111/j.1365-2664.2010.01826.x

27. Baraloto C, Forget PM, Goldberg DE. Seed mass, seedling size and neotropical tree seedling establishment. J Ecol. 2005;93(6):1156-66. doi:10.1111/j.1365-2745.2005.01041.x

28. Kitajima K. Do shade-tolerant tropical tree seedlings depend longer on seed reserves? Functional growth analysis of three Bignoniaceae species. Funct Ecol. 2002;16(4):433-44. doi:10.1046/j.1365-2435.2002.00641.x

29. Moles AT, Falster DS, Leishman MR, Westoby M. Small-seeded species produce more seeds per square metre of canopy per year, but not per individual per lifetime. J Ecol. 2004;92(3):384-96. doi:10.1111/j.0022-0477.2004.00880.x

30. Doua-Bi GYA, Zo-Bi IC, Amani BHK, Elogne AGM, N'dja JK, N'Guessan AE, et al. Taking advantage of natural regeneration potential in secondary forests to recover commercial tree resources in Côte d'Ivoire. For Ecol Manag. août 2021;493(May):119240-119240. doi:10.1016/j.foreco.2021.119240

31. Châtelain C, Aké-Assi L, Spichiger R, Gautier L. Cartes de distribution des plantesde Côte d'Ivoire. Conservatoire et Jardin botanique de la Ville de Genève. Vol. 64. Suisse; 2011. 327 p.

32. OIPR. Plan d'Aménagement et de Gestion (PAG) du Parc national de Taï 2014-2018. [Rapport d'aménagement]. 2014. p. 131 p.

33. OIPR. Plan d'Aménagement et de Gestion du Parc national de Taï (PAG) 2020-2029 [Rapport stratégique de planification environnementale]. Abidjan, Côte d'Ivoire; 2020. p. 208.

34. Kouamélan E.P. Biodiversité des poissons du Parc national de Taï : Une ressource naturelle à sauvegarder. Éditionns Universitaires Européennes. In. Abidjan (Côted'Ivoire); 2018. p. 165 p.

35. Van Rompaey R, Adou Yao C, Déngueadhé KBE, Aké-Assi L, Wittebolle G, N'Guessan K. Biodiversité végétale et écologie du paysage dans le Sud du Parc national

deTaï (Côte d'Ivoire) [Rapport de synthèse de projet 04/97-1111a du Programme Tropenbos Côte d'Ivoire]. Wageningen, Pays-Bas; 2001. p. 42 p.

36. Bakayoko A, Neuba DFR, Kouadio YL, Nehoun B. Suivi de la restauration végétale naturelle des zones aménagées dans les secteurs de Djapadji, Soubré et ADK/V6 du Parc national de Taï. [Rapport de suivi des zones récupérées]. Abidjan (Côte d'Ivoire); 2018. p. 24 p.

37. Aké-Assi L. Flore de la Côte-d'Ivoire: Catalogue Systématique, Biogéographie et Écologie. 2001. (Boissiera 57–58. Conservatoire et Jardin botanique de la Ville de Genève).

38. Hutchinson J, Dalziel JM. Flora of West Tropical Africa. 2nd ed. Keay RWJ, Hepper FN, editors. Vol I–III. London: Crown Agents for Overseas Governments and Administrations. 1954–1972 p.

39. Réjou-Méchain M, Tanguy A, Piponiot C, Chave J, Hérault B. biomass: an r package for estimating above-ground biomass and its uncertainty in tropical forests. Methods Ecol Evol. sept 2017;8(9):1163-7. doi:10.1111/2041-210X.12753

40. De La Mensbruge G. La germination et les plantules des essences arborées de la forêt dense humide de la Côte D'Ivoire. CTFT. 1966. 389 p.

41. Dufrêne M, Legendre P. Species Assemblages and Indicator Species:the Need for a Flexible Asymmetrical Approach. Ecol Monogr. 1997;67(3):345-66. doi:10.1890/0012-9615(1997)067[0345:SAAIST]2.0.CO;2

42. Marcon E, Hérault B. entropart: An R Package to Measure and Partition Diversity. J Stat Softw. 7 oct 2015;67:1-26. doi:10.18637/jss.v067.i08

43. Maurent E, Hérault B, Piponiot C, Derroire G, Delgado D, Finegan B, et al. A common framework to model recovery in disturbed tropical forests. Ecol Model. sept 2023;483:110418-110418. doi:10.1016/J.ECOLMODEL.2023.110418

44. Carpenter B, Gelman A, Hoffman MD, Lee D, Goodrich B, Betancourt M, et al. Stan: A Probabilistic Programming Language. J Stat Softw. 11 janv 2017;76:1-32. doi:10.18637/jss.v076.i01

45. Hérault B, Piponiot C. Key drivers of ecosystem recovery after disturbance in a neotropical forest: Long-term lessons from the Paracou experiment, French Guiana. For Ecosyst. déc 2018;5(1):2. doi:10.1186/s40663-017-0126-7

46. Barros TC, Elias F, Romano LL, Ferreira J. Natural recovery of plant species diversity in secondary forests in Eastern Amazonia: contributions to passive forest restoration. Braz J Bot. 1 mars 2020;43(1):165-75. doi:10.1007/s40415-020-00585-9

47. Chazdon RL, Letcher SG, Van Breugel M, Martínez-Ramos M, Bongers F, Finegan B. Rates of change in tree communities of secondary Neotropical forests following major disturbances. Philos Trans R Soc B Biol Sci. févr 2007;362(1478):273-89. doi:10.1098/rstb.2006.1990

48. Lennox GD, Gardner TA, Thomson JR, Ferreira J, Berenguer E, Lees AC, et al. Second rate or a second chance? Assessing biomass and biodiversity recovery in regenerating Amazonian forests. Glob Change Biol. 2018;24(12):5680-94. doi:10.1111/gcb.14443

49. Chazdon RL, Norden N, Colwell RK, Chao A. Monitoring recovery of tree diversity during tropical forest restoration: lessons from long-term trajectories of natural regeneration. Philos Trans R Soc B Biol Sci. 14 nov 2022;378(1867):20210069. doi:10.1098/rstb.2021.0069

50. Deng F, He Y, Zang R, Deng F, He Y, Zang R. Recovery of Functional Diversity Following Shifting Cultivation in Tropical Monsoon Forests. Forests. 2018;9(9):1-20. doi:10.3390/f9090506

51. Makelele IA, Verheyen K, Boeckx P, Cizungu Ntaboba L, Mujinya Bazirake B, Ewango C, et al. Afrotropical secondary forests exhibit fast diversity and functional recovery, but slow compositional and carbon recovery after shifting cultivation. Carlucci M, éditeur. J Veg Sci. sept 2021;32(5):e13071. doi:10.1111/jvs.13071

52. Rozendaal DMA, Bongers F, Aide TM, Alvarez-Dávila E, Ascarrunz N, Balvanera P, et al. Biodiversity recovery of Neotropical secondary forests. Sci Adv. mars 2019;5(3):eaau3114. doi:10.1126/sciadv.aau3114

53. Koffi A.B., Tondoh E.J., Ehouman E., Freycon V., Piponiot C., Amani B.H.K, et al. Drivers of soil organic carbon recovery during secondary succession along a West African climate gradient. Ecol Indic. 2025;178:1-11. doi:10.1016/j.ecolind.2025.114073

54. Konan IK, N'Guessan AE, Dago MR, Kouassi A, N'dja JK, Aussenac R, et al. Maximizing tree diversity in cocoa agroforestry: taking advantage of planted, spontaneous, and remnant trees. Agrofor Syst. 2025;99(5):1-14. doi:10.1007/s10457-025-01180-1

55. Kouassi AK, Zo-Bi IC, Aussenac R, Kouamé IK, Dago MR, N'guessan AE, et al. The great mistake of plantation programs in cocoa agroforests – Let's bet on natural regeneration to sustainably provide timber wood. Trees For People. 1 juin 2023;12:100386. doi:10.1016/j.tfp.2023.100386

56. N'Guessan AE, Hérault B, Zo-Bi IC, Aussenac R, Dago MR, Konan IK, et al. Rethinking cocoa agroforestry: towards sustainable coexistence of remnant, spontaneous and planted trees. Perspective. 20 janv 2026;(70):1-4. Located at: Côte d'Ivoire, West Africa. doi:10.19182/perspective/38076

57. Houphouët ADL, Sangne YC, Diarrassouba A, Adou Yao YC, Betbeder J, Hérault B. Forest structure recovery around West Africa's last great rainforest: Modelling complex dynamics in Taï national park. Trees For People. déc 2025;22:1-12. doi:10.1016/j.tfp.2025.101005

58. Albert KM. Role of revegetation in restoring fertility of degraded mined soils in Ghana: A review. Int J Biodivers Conserv. 28 févr 2015;7(2):57-80. doi:10.5897/IJBC2014.0775

59. Pérez-Cárdenas N, Mora F, Arreola-Villa F, Arroyo-Rodríguez V, Balvanera P, Flores-Casas R, et al. Effects of landscape composition and site land-use intensity on secondary

succession in a tropical dry forest. For Ecol Manag. 15 févr 2021;482:118818. doi:10.1016/j.foreco.2020.118818

60. Tucker MM, Kashian DM. Pre-fire forest remnants affect post-fire plant community structure and composition. For Ecol Manag. janv 2018;408:103-11. doi:10.1016/J.FORECO.2017.10.038

61. Wu MM, Liang Y, He HS, Liu B, Ma T. Remnant trees location and abundance play different roles in forest landscape recovery. For Ecol Manag. 1 mai 2022;511:120154. doi:10.1016/j.foreco.2022.120154

62. Sandor ME, Chazdon RL. Remnant Trees Affect Species Composition but Not Structure of Tropical Second-Growth Forest. Farwig N, éditeur. PLoS ONE. 13 janv 2014;9(1):e83284. doi:10.1371/journal.pone.0083284

63. Sterck FJ, Poorter L, Schieving F. Leaf traits determine the growth-survival trade-off across rain forest tree species. Am Nat. mai 2006;167(5):758-65. doi:10.1086/503056

64. Brodie JF, Helmy OE, Brockelman WY, Maron JL. Bushmeat poaching reduces the seed dispersal and population growth rate of a mammal-dispersed tree. Ecol Appl. 2009;19(4):854-63. doi:10.1890/08-0955.1

65. Markl JS, Schleuning M, Forget PM, Jordano P, Lambert JE, Traveset A, et al. Meta-Analysis of the Effects of Human Disturbance on Seed Dispersal by Animals. Conserv Biol. 2012;26(6):1072-81. doi:10.1111/j.1523-1739.2012.01927.x

66. Laurance WF. Theory meets reality: How habitat fragmentation research has transcended island biogeographic theory. Biol Conserv. 1 juill 2008;141(7):1731-44. doi:10.1016/j.biocon.2008.05.011

67. Murcia C. Edge effects in fragmented forests: implications for conservation. Trends Ecol Evol. 1 févr 1995;10(2):58-62. doi:10.1016/S0169-5347(00)88977-6

68. Traoré S, Zo-Bi IC, Piponiot C, Aussenac R, Hérault B. Fragmentation is the main driver of residual forest aboveground biomass in West African low forest-high deforestation landscapes. Trees For People. 1 mars 2024;15:100477. doi:10.1016/j.tfp.2023.100477

69. Cuni-Sanchez A, White LJT, Calders K, Jeffery KJ, Abernethy K, Burt A, et al. African Savanna-Forest Boundary Dynamics: A 20-Year Study. Gomory D, éditeur. PLOS ONE. 2016;11(6):156-934. doi:10.1371/journal.pone.0156934

70. Amani BHK, N'Guessan AE, Van der Meersch V, Derroire G, Piponiot C, Elogne AGM, et al. Lessons from a regional analysis of forest recovery trajectories in West Africa. Environ Res Lett. nov 2022;17(11). doi:10.1088/1748-9326/ac9b4f

71. Pouteau R, Picard J, Doumenge C, Brncic T, Gillet JF, Doucet JL, et al. The puzzling ecology of African Marantaceae forests. Am J Bot. 2024;111(4):e16320. doi:10.1002/ajb2.16320

72. Poorter L, Rozendaal DMA, Bongers F, Almeida DJS, Álvarez FS, Andrade JL, et al. Functional recovery of secondary tropical forests. Proc Natl Acad Sci. déc 2021;118(49):e2003405118-e2003405118. doi:10.1073/pnas.2003405118

73. Liang J, Crowther TW, Picard N, Wiser S, Zhou M, Alberti G, et al. Positive biodiversity-productivity relationship predominant in global forests. Science. oct 2016;354(6309). doi:10.1126/science.aaf8957

74. Hérault B, Bachelot B, Poorter L, Rossi V, Bongers F, Chave J, et al. Functional traits shape ontogenetic growth trajectories of rain forest tree species. J Ecol. nov 2011;99(6):1431-40. doi:10.1111/j.1365-2745.2011.01883.x

75. Norden N, Chazdon RL, Chao A, Jiang YH, Vílchez-Alvarado B. Resilience of tropical rain forests: tree community reassembly in secondary forests. Ecol Lett. mai 2009;12(5):385-94. doi:10.1111/j.1461-0248.2009.01292.x

76. Cavaleri MA, Oberbauer SF, Clark DB, Clark DA, Ryan MG. Height is more important than light in determining leaf morphology in a tropical forest. Ecology. juin 2010;91(6):1730-9. doi:10.1890/09-1326.1 PubMed PMID: 20583714.

77. Coble AP, Cavaleri MA. Light acclimation optimizes leaf functional traits despite height-related constraints in a canopy shading experiment. Oecologia. 1 avr 2015;177(4):1131-43. doi:10.1007/s00442-015-3219-4

78. Dalling JW, Hubbell SP, Silvera K. Seed dispersal, seedling establishment and gap partitioning among tropical pioneer trees. J Ecol. 1998;86(4):674-89. doi:10.1046/j.1365-2745.1998.00298.x

79. Peña-Claros M, Boo HD. The effect of forest successional stage on seed removal of tropical rain forest tree species. J Trop Ecol. mars 2002;18(2):261-74. doi:10.1017/S0266467402002171

80. Poorter L, Rose SA. Light-dependent changes in the relationship between seed mass and seedling traits: a meta-analysis for rain forest tree species. Oecologia. 1 janv 2005;142(3):378-87. doi:10.1007/s00442-004-1732-y

81. Konaté I, Ehouman E, Wourro F, Doua-Bi Y, Tiéoulé F, Coulibaly B, et al. The anatomy of survival — What determines seedling fate after planting in West Africa? For Ecol Manag. 15 déc 2025;598:123266. doi:10.1016/j.foreco.2025.123266

82. Muller-Landau HC. The tolerance–fecundity trade-off and the maintenance of diversity in seed size. Proc Natl Acad Sci U S A. 2 mars 2010;107(9):4242-7. doi:10.1073/pnas.0911637107 PubMed PMID: 20160078; PubMed Central PMCID: PMC2840174.

83. Banks-Leite C, Ewers RM, Kapos V, Martensen AC, Metzger JP. Comparing species and measures of landscape structure as indicators of conservation importance. J Appl Ecol. 2011;48(3):706-14. doi:10.1111/j.1365-2664.2011.01966.x

84. Barlow J, Gardner T, Araujo I, Ávila-Pires TC, Bonaldo AB, Costa JE, et al. Quantifying the biodiversity value of tropical primary, secondary and plantation forests. Proc Natl Acad Sci. 20 nov 2007;104(47):18555-60. doi:10.1073/pnas.0703333104

85. Swanson ME, Franklin JF, Beschta RL, Crisafulli CM, DellaSala DA, Hutto RL, et al. The forgotten stage of forest succession: early-successional ecosystems on forest sites. Front Ecol Environ. 2011;9(2):117-25. doi:10.1890/090157

86. Seidl R, Rammer W, Spies TA. Disturbance legacies increase the resilience of forest ecosystem structure, composition, and functioning. Ecol Appl. 2014;24(8):2063-77. doi:10.1890/14-0255.1

87. Anny Estelle N'Guessan, N'dja JK, Yao ON, Amani BHK, Gouli RGZ, Piponiot C, et al. Drivers of biomass recovery in a secondary forested landscape of West Africa. For Ecol Manag. févr 2019;433:325-31. doi:10.1016/j.foreco.2018.11.021

Supporting information

Appendix S1. IUCN-listed tree species recorded in Taï National Park (Côte d'Ivoire). Species classified as Endangered (EN) or Vulnerable (VU) according to the IUCN Red List. Values of 1 indicate the assigned conservation category

Appendix S2. Old-growth indicator species identified using the Dufrêne–Legendre indicator value method. Indicator species associated with intact old-growth forests, identified using the Dufrêne–Legendre indicator value (IndVal) method. Only species with significant association ($P < 0.002$) are reported. IndVal values range from 0 (no association) to 1 (perfect specificity and fidelity to old-growth forests).

852

Figure 1

Click here to access/download;Figure;Fig 1.tif

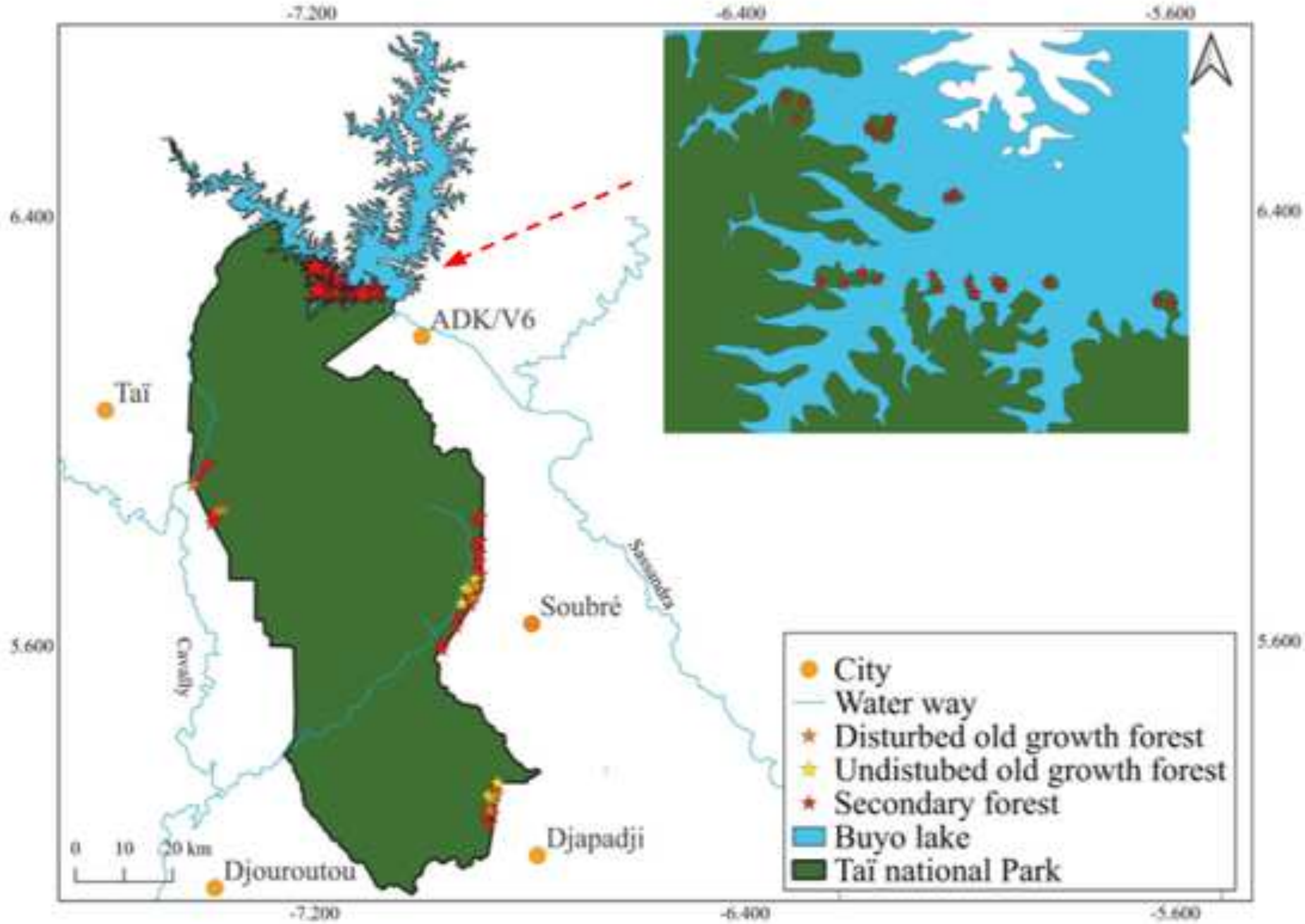

Figure 3

Click here to access/download;Figure;fig3.png

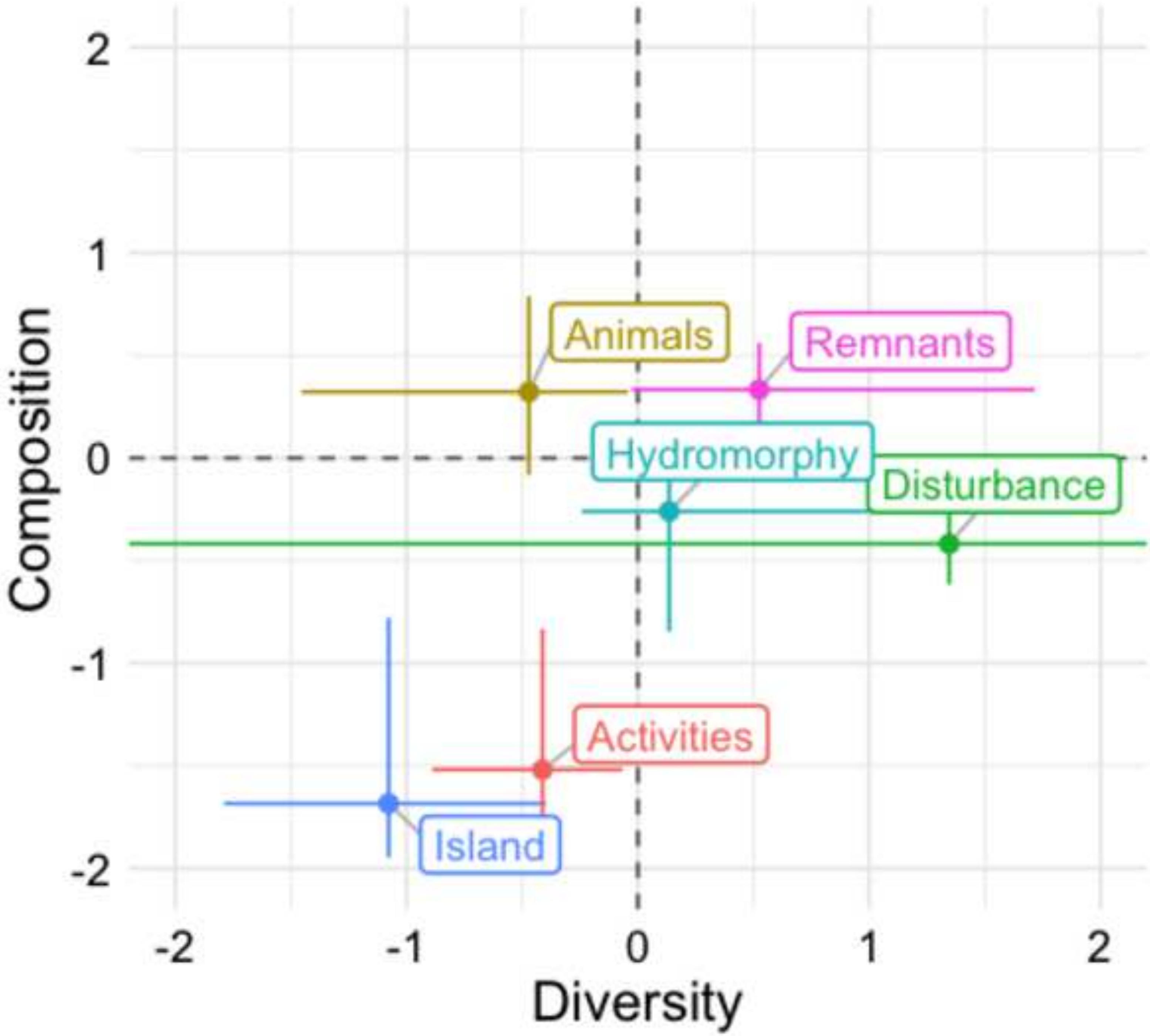

Figure 4

Click here to access/download;Figure;fig4.png

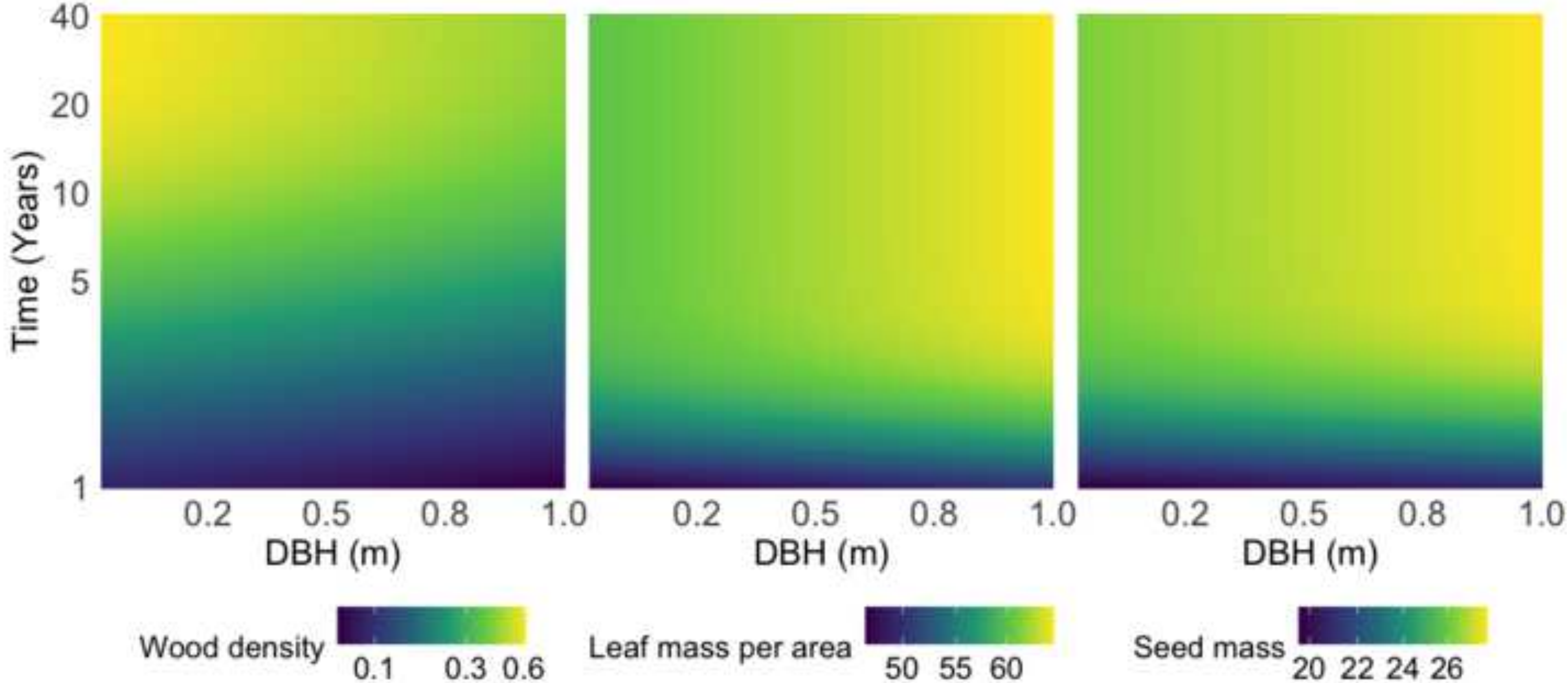

Figure 5

Click here to access/download;Figure;fig5.png

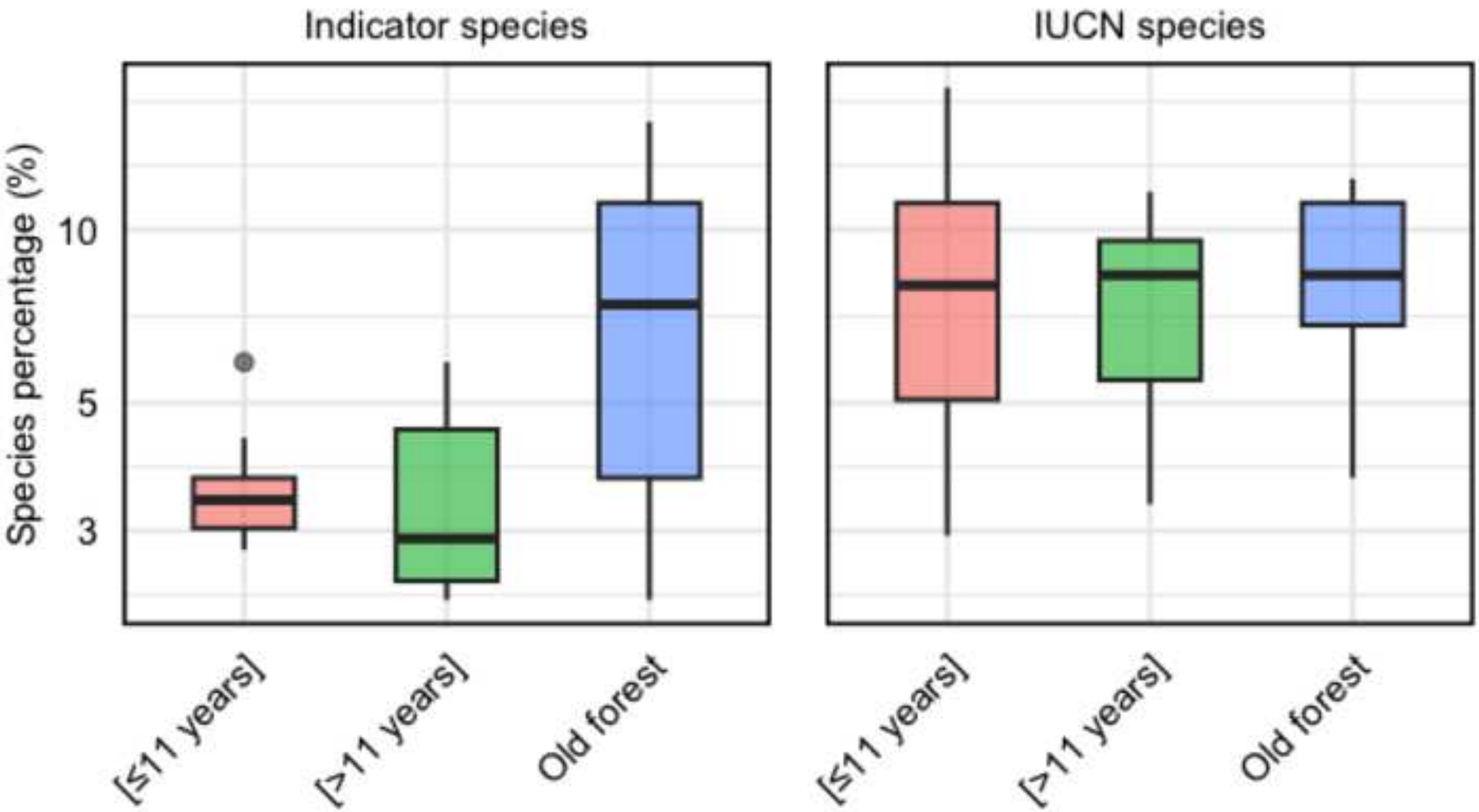

Figure 2

Click here to access/download;Figure;fig2.tif

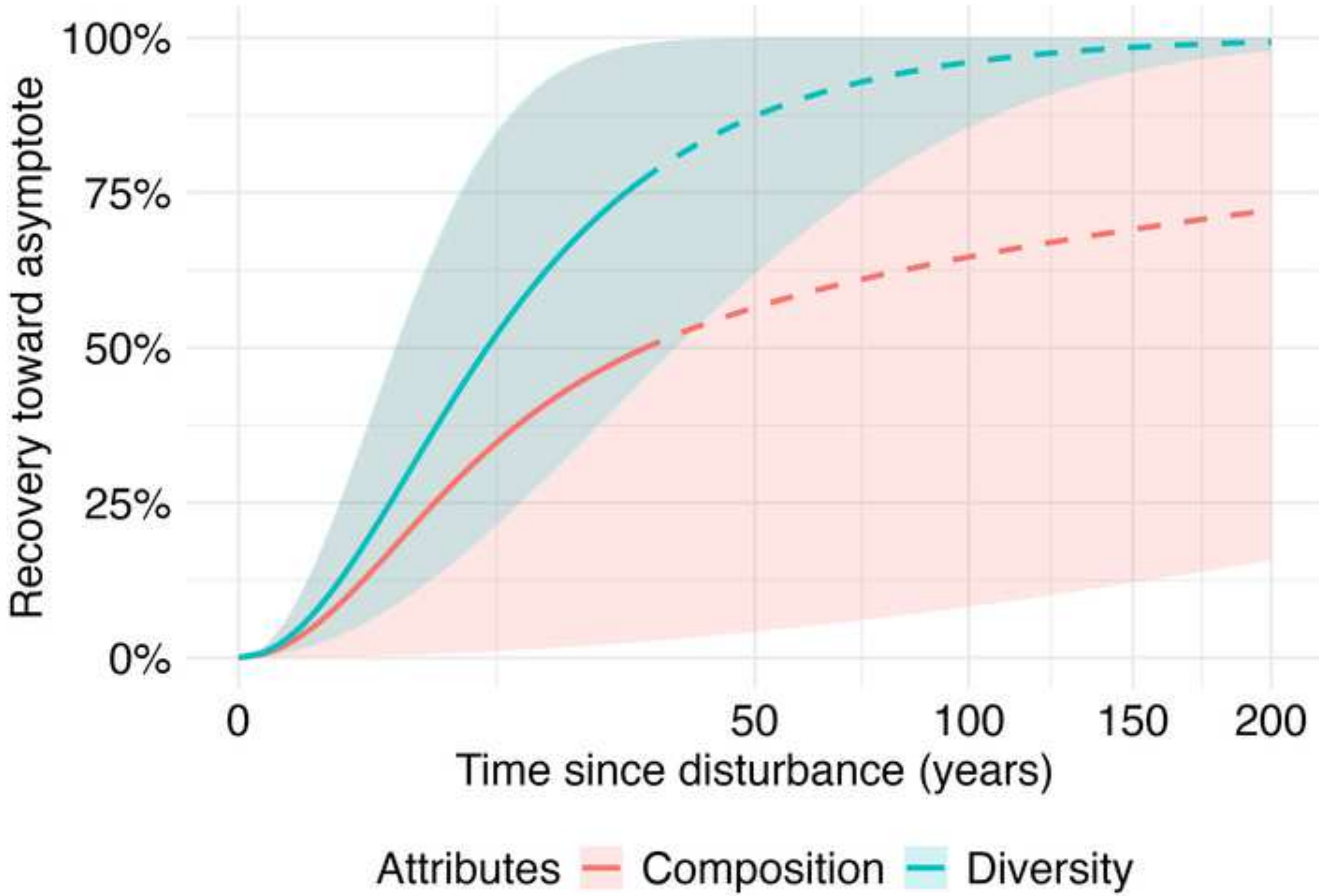

Click here to access/download
Supporting Information
S1 Appendix. IUCN listed tree species.docx

Click here to access/download
Supporting Information
S2 Appendix. Old-growth indicator species.docx

Click here to access/download
Supporting Information
Inclusivity-in-global-research-questionnaire_FILLED.docx

Original Copyright holder of Figure 1

Click here to access/download
Supporting Information
Doc Houphouet.pdf

Regenerating forests around Taï National Park sustain biodiversity despite constrained floristic recovery

Aya Diane Larissa Houphouët [1**], Yao Charles Sangne [1], Abdoulaye Diarrassouba [2], Evans Ehouman[3,4], Bruno Hérault [3,4*]

[1] Université Jean Lorougnon Guédé, Unité de Formation et de Recherche en Environnement, Daloa, Côte d'Ivoire

[2] Office Ivoirien des Parcs et Réserves, Soubré, Côte d'Ivoire

[3] CIRAD, Forêts et Sociétés, F-34398, Montpellier, France

[4] Forêts et Sociétés, Univ Montpellier, CIRAD, Montpellier, France

***Corresponding authors**

*bruno.herault@cirad.fr (BH)

**larissahouphouet@gmail.com (ADLH)

ABSTRACT

Extensive deforestation and disturbance have reshaped forest landscapes in West Africa, including areas surrounding Taï National Park, the largest remaining block of Upper Guinean rainforest. Although many degraded areas are currently undergoing regeneration, the capacity of these secondary forests to recover their biodiversity attributes remains insufficiently assessed within this park. This study quantifies the multidimensional restoration of biodiversity across 125 secondary forest plots and 15 primary forest plots representing 14,731 inventoried individuals in four sectors of the park. Using a Bayesian modelling framework, we estimate recovery trajectories of Shannon diversity, floristic composition, functional traits (wood density, leaf mass per area, seed mass), and conservation-relevant species. The different biodiversity attributes recovered at contrasting rates. Diversity was restored more rapidly (λ = 0.06) than floristic composition (λ = 0.03), and these recovery varied according to environmental variables. Among these, the presence of remnant trees showed the highest median on diversity (0.53 ± 0.61) and floristic composition (0.37 ± 0.17), promoting the rapid recovery of both attributes, followed by prior land use, particularly cocoa farming which also positively influenced the recovery of alpha diversity (λ = 0.03) and floristic composition (λ = 0.02). Regarding functional traits, they displayed contrasting dynamics: specific leaf area and seed mass recovered rapidly along successional gradients, whereas wood density followed a more gradual recovery trajectory. From a conservation perspective, although the proportion of IUCN Red List species remained stable along the successional gradient, old-growth indicator species were significantly more abundant in primary forests. To ensure the long-term conservation of biodiversity, protecting both primary and regenerating forests is essential to preserve the ecological resilience of this park.

Introduction

Tropical forests in West Africa harbour exceptionally high levels of biodiversity and form part of one of the world’s 25 biodiversity hotspots [1,2]. Despite their ecological importance, these forests have experienced intense deforestation in recent decades, resulting in substantial biodiversity loss [3]. In Côte d’Ivoire, forest conversion has been primarily driven by agricultural expansion, especially cocoa, coffee, and oil palm alongside logging, charcoal production, infrastructure development, and mining [3–5]. These transformations have led not only to biodiversity decline but also to the degradation of ecosystem services and climate regulation.

Against this backdrop of widespread forest loss, restoring degraded lands has emerged as an essential complementary strategy to the conservation of remaining old-growth forests [6]. Secondary forests now represent a substantial proportion of tropical forest cover [7–9] and may contribute meaningfully to biodiversity conservation [10,11]. However, recovery is complex and does not progress equally across ecological features. Although species diversity often increases relatively rapidly following agricultural abandonment [12,13], floristic composition may take decades to converge toward old-growth conditions [14,15]. Moreover, recovery trajectories are strongly shaped by land-use intensity, soil degradation, remnant trees that enhance seed dispersal, and by the connectivity between neighboring landscapes [16–19].

Importantly, biodiversity recovery cannot be fully understood from taxonomic patterns alone. Beyond changes in species richness and composition, ecological succession entails shifts in species’ life-history strategies and functional attributes [20]. Early successional stages are typically dominated by fast-growing pioneer species, whereas late-successional and shade-tolerant species establish progressively as forest structure develops [14,21]. These successional dynamics are reflected in changes in functional traits, which provide mechanistic insight into

ecosystem functioning and resilience [22]. Traits such as wood density, leaf mass per area (LMA), and seed mass are closely linked to growth–mortality trade-offs, resource-use strategies, and regeneration processes [23–26]. Seed mass, in particular, mediates the balance between colonization ability and establishment success [27–29]. Integrating functional composition with taxonomic and compositional analyses therefore provides a more comprehensive framework for evaluating forest recovery.

In West Africa, where remaining old-growth forests are extremely limited and deforestation rates remain among the highest globally, conservation increasingly depends on the capacity of secondary forests to sustain biodiversity. Yet it remains unclear whether regenerating forests can meaningfully substitute for primary forests in maintaining endangered species, functional diversity, and ecosystem processes. Addressing this uncertainty is critical for guiding restoration strategies, landscape planning, and protected-area buffering. These issues are especially critical in landscapes subjected to intense anthropogenic pressure, such as those surrounding Taï National Park in south-western Côte d'Ivoire. Taï National Park constitutes the largest remaining continuous block of Upper Guinean humid rainforest and one of the last extensive remnants of tropical rainforest in West Africa. As a major stronghold of regional biodiversity, it harbours numerous endemic and threatened species. However, episodes of degradation particularly during the 2002-2011 politico-military crises have affected both the park and its periphery. Since 2015, degraded areas have been undergoing natural regeneration [30], creating a mosaic of intact old-growth forest, disturbed old-growth forest, and secondary forests of varying ages. In such a globally significant forest landscape, understanding the extent and pace of biodiversity recovery is essential for informing regional conservation and restoration strategies.

To date, few studies in Upper Guinean forests have jointly quantified taxonomic, functional, compositional, and conservation-relevant recovery using reference-based models. Here, we provide one of the first multidimensional assessments of natural forest regeneration surrounding Taï National Park, integrating community composition, functional traits, biomass, and species conservation status within a unified Bayesian framework. To provide conceptual clarity, we define "recovery" as the degree of convergence of biodiversity attributes in secondary forests toward reference conditions observed in intact old-growth forests. Recovery therefore refers not only to increases in species diversity over time, but also to compositional similarity and functional convergence relative to old-growth benchmarks. This reference-based approach allows us to distinguish between rapid gains in taxonomic diversity and slower or incomplete recovery of compositional and functional attributes. Building on this framework, we analyse biodiversity recovery trajectories in secondary forests surrounding Taï National Park. Specifically, we address the following questions: (i) What are the recovery trajectories of Shannon diversity and floristic composition, and which factors influence them? (ii) How do key functional traits such as wood density, leaf mass per area, and seed mass change along secondary succession, and can they serve as indicators of forest recovery? and (iii) Do secondary forests contribute to the persistence and recovery of conservation-relevant species?

Methods

Study Area

The study was conducted in and around Taï National Park (TNP), located in south-western of Côte d'Ivoire between 5°15′ to 6°07′ N and 7°25′ to 7°54′ W (Fig 1). TNP constitutes the largest remaining continuous block of Upper Guinean humid rainforest and is recognised as a UNESCO World Heritage Site [31,32]. The park covers approximately 5,360 km² and represents one of the last extensive remnants of primary tropical rainforest in West Africa [33] . The region is characterised by a humid tropical climate with two rainy seasons (March to July and September to November) and two relatively drier periods. Mean annual rainfall ranges between 1,700 and 2,200 mm, and mean annual temperature is approximately 25–27 °C. Soils are predominantly ferralitic and vary locally depending on topography and drainage conditions. Vegetation is dominated by evergreen and semi-deciduous moist forest. The park harbours high levels of plant and vertebrate diversity, including numerous endemic and threatened species characteristic of the Upper Guinean forest block [34,35]. Surrounding landscapes have been extensively transformed by agricultural expansion, particularly cocoa cultivation, as well as logging and artisanal gold mining.

During the post-electoral crises of 2000 and 2010, reduced surveillance led to forest encroachment and degradation in parts of the park and its periphery. Since 2015, restoration and natural regeneration processes have been ongoing in previously degraded areas [36]. As a result, the current landscape forms a mosaic of intact old-growth forest, disturbed old-growth forest, and secondary forests of varying ages originating from former agricultural lands or mining sites.

Fig. 1. Spatial distribution of sampling plots across Taï National Park and surrounding landscapes in south-western Côte d'Ivoire. Sampling plots represent a chronosequence of secondary forests (9–26 years since abandonment; n = 125) and reference intact old-growth forests (n = 15). Plots are distributed within and around park boundaries. The map also shows Buyo Lake, major waterways, and nearby cities to provide geographic context. The inset highlights the northern sampling cluster near Buyo Lake, where forest fragmentation has created a mosaic of secondary and old-growth stands. Printed under a CC BY license, with permission from OIPR, original copyright [2026].

Vegetation Data

A total of 140 vegetation plots were established in and around Taï National Park. Of these, 125 plots were located in secondary forests and 15 plots in intact old-growth forests, which served as reference sites. Each plot measured 25 × 25 m (0.0625 ha). Secondary forests represented a chronosequence ranging from 9 to 26 years since abandonment. Forest age was determined based on information from local communities, park management records, and field evidence of past land use. Following the post-electoral crisis period, agricultural activities inside the park were discontinued and cocoa plantations were progressively removed as part of restoration and management interventions. Subsequent natural succession gave rise to stands of varying ages, forming the basis of the chronosequence analysed in this study. Within each 25 × 25 m plot, all woody stems with a diameter at breast height (DBH) ≥ 2.5 cm were inventoried. DBH was measured at 1.3 m above ground level. For multi-stemmed individuals, each stem meeting the diameter threshold was recorded separately. Lianas were excluded from the inventory. Species were identified in the field by experienced botanists. When identification was uncertain, specimens were examined using the regional floras of Aké-Assi [37] and Hutchinson & Dalziel [38] to ensure accurate determination. Although voucher specimens were not systematically deposited in a public herbarium, taxonomic verification procedures were applied to ensure consistency and reliability across plots. Nomenclature was standardised according to the

Angiosperm Phylogeny Group IV (APG IV) classification system, and species names were harmonised to avoid synonymy or spelling inconsistencies within the dataset.

All original data used in this study will be publicly available and can be accessed from Zenodo upon acceptance. Permissions for this study were granted by the Office Ivoirien des Parcs et Réserves (OIPR), the governmental body responsible for managing Taï National Park, Côte d'Ivoire.

Functional Trait Data

We analysed three functional traits : wood density (WD), leaf mass per area (LMA), and seed mass. These traits were selected because of their established links to growth–mortality trade-offs, resource-use strategies, regeneration processes, and successional dynamics in tropical forests [23,24] .

Wood density values were retrieved using the R package *BIOMASS* [39] through the function *getWoodDensity()*, which assigns species-level values when available and substitutes genus-level averages when species-specific data are missing. This procedure ensured complete coverage of wood density values across all species in the dataset. Seed mass and LMA values were obtained primarily from the TRY global plant trait database [40], including regional datasets integrated into TRY [41] . Seed mass data were additionally compiled from the SER-SID database (ser-sid.org). For species lacking values in these databases, seed mass measurements were supplemented using published data from [42] .

When multiple trait values were available for a given species across datasets, the median value was used to reduce the influence of extreme observations. Species-level trait values were then assigned to all corresponding individuals in the vegetation dataset. Trait coverage varied among

traits. Wood density values were available for 100% of species, seed mass for approximately 49% of individuals, and LMA for 56% of individuals.

Environmental variables

To account for ecological and anthropogenic drivers of recovery, we recorded a set of environmental and disturbance-related variables expected to influence regeneration processes (Table 1).

- Large and medium-sized mammals were included because they play a key role in seed dispersal and recruitment dynamics in tropical forests. Their presence can enhance colonization by late-successional species and influence community composition. Mammal occurrence was assessed through indirect field signs of species known to function as seed dispersers or large herbivores.
- Remnant trees were quantified as indicators of biological legacies from pre-disturbance forests. Such trees can provide local seed sources, structural complexity, and perching sites for frugivores, thereby potentially accelerating succession.
- Soil hydromorphy was recorded as a proxy for edaphic constraints on species establishment and growth, as waterlogged conditions can limit recruitment of certain functional groups.
- Island configuration was included to capture landscape isolation effects, which may reduce propagule influx and alter recovery trajectories.
- Signs of ongoing human activities were documented to account for continued disturbance pressure that may slow or redirect successional dynamics.

Together, these variables capture biotic, abiotic, and anthropogenic drivers of succession and were incorporated as covariates in subsequent models of biodiversity recovery.

Table 1. Plot-level environmental variables used to assess biotic legacies, edaphic constraints, landscape isolation, and ongoing anthropogenic activities potentially influencing biodiversity recovery in secondary forests surrounding Taï National Park, Côte d'Ivoire.

Variables	Description	Values
Mammal	Number of large/medium-sized mammal species detected through indirect signs	0-3
Hydromorphy	Presence of hydromorphic soil features in surface horizons	0/1
Island	Plot located in isolated island	0/1
Remnants	Density of isolated old-growth trees persisting after land conversion	trees.ha^{-1}
Activities	Number of disturbance types observed (poaching, agriculture,mining)	0-3

Conservation-relevant species

To evaluate the contribution of secondary forests to biodiversity conservation, we considered two categories of conservation-relevant species: (i) old-growth indicator species and (ii) species listed under threatened categories of the IUCN Red List.

Indicator species characteristic of old-growth forests were identified using the method of [43] , implemented in the R package *indicspecies*. We compared two groups of plots: intact old-growth forests and secondary forests younger than 20 years. The indicator value (IndVal) combines measures of specificity and fidelity to a given habitat type, allowing the identification of species strongly associated with old-growth conditions. Species were considered significant old-growth indicators when their indicator value was associated with a permutation test P-value < 0.002. A stringent significance threshold was applied to reduce the risk of false positives and

to ensure robust identification of species reliably associated with old-growth forest conditions. For each plot, we subsequently quantified the occurrence and abundance of these indicator species.

For all inventoried species, conservation status was assessed using the IUCN Red List of Threatened Species (https://www.iucnredlist.org/). Species classified as Vulnerable (VU) or Endangered (EN) were considered conservation-relevant for this analysis. The presence and abundance of these species were quantified across forest types to evaluate their persistence and potential recovery during secondary succession.

Biodiversity metrics

Diversity was quantified using Shannon diversity, expressed as the Hill number of order 1 ($q = 1$). Diversity of order 1 corresponds to the exponential of Shannon entropy and represents the effective number of species in a community. Estimates were computed using the R package *entropart* [44]. Sample coverage was first estimated for each plot using the function *coverage()*. As coverage was consistently above 0.90 across plots, diversity was estimated at a standardized coverage level of 0.90 to ensure comparability among plots. This coverage-based standardization minimizes bias due to incomplete sampling while avoiding distortions caused by unequal sampling effort.

Composition reflects both species identity and their relative abundances within each plot. To quantify compositional similarity between secondary forests (SF) and intact old-growth forests (OGF), we calculated the abundance-based Chao–Jaccard similarity index for all possible SF–OGF plot pairs. This index accounts for unseen shared species and corrects for undersampling, thereby providing a robust estimate of compositional similarity. To define the reference state, similarities among old-growth plots (OGF–OGF) were also calculated. These within-OGF

similarities represent the natural variability of intact forests and define the benchmark toward which secondary forests are expected to converge during succession [15]. Although the 15 reference plots are modest relative to the park's total area, they were distributed across four sectors of the park to capture part of its spatial heterogeneity, and their pairwise similarity was used to explicitly characterise — rather than assume — the natural variability of old-growth forest composition used as the recovery benchmark.

Modeling Strategies

All models were built within a Bayesian framework. A Bayesian approach was chosen because of its flexibility in handling complex hierarchical structures, its ability to accommodate different likelihood distributions across response variables, and its explicit quantification of uncertainty through posterior distributions. A log-normal likelihood was used because all response variables considered (diversity, composition, and functional traits) are strictly defined on the positive real line ($\mathbb{R}^+$) and exhibited right-skewed distributions [45]. Log-normal likelihoods were implemented in Stan using its standard parameterization, with mean parameters defined on the original scale and passed to the distribution through the log link. Models were implemented in Stan and fitted using Hamiltonian Monte Carlo (HMC) sampling for parameter estimation [46].

Q1- Biodiversity Recovery Trajectories

To estimate recovery trajectories and intrinsic recovery rates, we applied the exponential recovery model developed by [47]. This model assumes that the instantaneous recovery rate of an ecosystem attribute is proportional to the distance between its current state and its

asymptotic value [45]. Such a formulation captures saturating recovery dynamics commonly observed during secondary succession.

For secondary forests, the likelihood model for a biodiversity attribute X (diversity or composition) in plot *p* at time *t* since abandonment was specified as:

$$X_{p,t} \sim LN\left(\theta_0^X + (\ \theta_{+\infty}^X - \theta_0^X)\,.\left(1 - e^{-\lambda^X t_p}\right), \sigma\,_X^2\right) \qquad (1)$$

where:

- $X_{p,t}$ is the observed value of attribute *X* in plot *p* at time *t*,
- θ_0^X represents the initial value of *X* at the beginning of the recovery process,
- $\theta_{+\infty}^X$ is the asymptotic value of *X*, corresponding to the long-term equilibrium conditions,
- λ^X is the intrinsic recovery rate controlling the speed of convergence toward the asymptote,
- $\sigma\,_X^2$ represents the residual variance among secondary forest plots.

For intact old-growth forests, time since regeneration is not defined and attribute values are assumed to represent the asymptotic state. The likelihood was therefore specified as:

$$X_{p,t} \sim LN(\theta_{+\infty}^X, \sigma\,_X^2) \qquad (2)$$

This formulation allows old-growth forests to directly inform the asymptotic parameter $\theta_{+\infty}^X$. To account for anthropogenic disturbance effects in old-growth forests, asymptotic values were adjusted by including a disturbance effect term:

$$X_{p,t} \sim LN(\theta_{+\infty}^X + \theta_{dist}^X, \sigma\,_X^2) \qquad (3)$$

where θ_{dist}^X represents the effect of disturbance intensity on the asymptotic value of attribute *X*.

To evaluate how environmental and disturbance-related variables influenced recovery dynamics, covariates (Table 1) were incorporated into the recovery rate parameter λ_p^X. Because recovery rates are strictly positive, we modelled them using an exponential link function:

$$\lambda_p^X = \lambda_{land}^X \times e^{\left(\sum_{j=1}^{J} \beta_{COV\,j}^X . COV_{j,p}\right)} \quad (4)$$

where:

- λ_{land}^X is the baseline recovery rate associated with previous land use,
- $\beta_{COV\,j}^X$ represents the effect of covariate *j* on recovery rate,
- $COV_{j,p}$ is the standardized value of covariate *j* for plot *p*.

All continuous covariates were standardized prior to analysis to facilitate parameter interpretation and comparison of effect sizes.

Q2 - Functional trait dynamics

To analyse how functional traits change during succession, we used an individual-level Bayesian model linking trait values to both stem diameter *DBH* and forest age *t*. This approach captures shifts in trait distributions along the tree size gradient during recovery, consistent with the idea that secondary forests progressively approach old-growth trait–size relationships as forests mature [47]. Because trait values were strictly positive and right-skewed, they were modelled using a log-normal likelihood. For each tree *n* in secondary forests, trait values *Y* (wood density, leaf mass per area, or seed mass) were modelled as:

$$Y_{n,p} \sim LN\left(\left(\alpha_Y + \beta_Y . DBH_n\right) . \left(1 - e^{-\lambda^Y t_p}\right), \sigma_y^2\right) \quad (5)$$

where DBH_n is the diameter of individual n, t_p is time since abandonment for the plot p where the individual occurs, α_Y is the baseline value of the trait when the diameter effect is null, β_Y quantifies the effect of DBH on the expected trait value, λ^Y is the convergence rate toward the asymptotic state, and σ_y^2 is the residual variance. Under this formulation, the expected trait–size relationship converges toward the old-growth reference function $(\alpha_Y + \beta_Y . DBH_n)$ as forest age increases, thereby allowing trait distributions to progressively align with old-growth conditions along the DBH gradient.

For intact old-growth forests, trait values were assumed to reflect the asymptotic (reference) trait–size relationship:

$$Y_n \sim LN\left((\alpha_Y + \beta_Y . DBH_n), \sigma_y^2\right) \qquad (6)$$

To account for possible anthropogenic disturbance shifts away from intact reference conditions, we included an additive disturbance effect on the expected trait–size relationship:

$$Y_n \sim LN\left(\theta_Y . (\alpha_Y + \beta_Y . DBH_n), \sigma_y^2\right) \qquad (7)$$

where θ_Y represents the effect of disturbance intensity on the expected value of trait Y (i.e., $\theta_Y > 1$ indicates higher expected trait values under disturbance, whereas $\theta_Y < 1$ indicates lower expected trait values).

Q3 - Conservation-relevant species

To assess whether conservation-relevant species are maintained or recovering during succession, we calculated, for each plot, the proportion of individuals belonging to (i) old-growth indicator species and (ii) IUCN-listed species (Vulnerable or Endangered). Secondary forests were grouped into two age classes (≤ 11 years and > 11 years since abandonment) and compared with intact old-growth forests. This comparison allowed us to evaluate whether the

relative representation of conservation-relevant species increases with forest age and approaches old-growth conditions.

Results

Across all forest types, we recorded 14,731 woody individuals (DBH $\geq$ 2.5 cm), corresponding to 174 species distributed among 138 genera and 48 families.

Biodiversity recovery trajectories

Shannon diversity and floristic composition did not recover at the same rate (Fig 2, Table 2). Shannon diversity increased rapidly during succession, reaching approximately 75% of its asymptotic value within the first 40 years. The intrinsic recovery rate was $\lambda = 0.06$ (90% CI: 0.02–0.17). Diversity started at an estimated 8.94 effective species (θ_0) and converged toward an asymptotic value of 20.39 in old-growth forests ($\theta_{+\infty}$). In contrast, floristic composition recovered more slowly ($\lambda = 0.03$; 90% CI: 0.00–0.16). Because λ is constrained to be strictly positive by the model's exponential link function, this interval does not literally include zero; however, its lower bound is very close to zero, indicating that a near-negligible, practically stagnant recovery rate cannot be excluded. On average, secondary forests required over 40 years to approach half of the asymptotic similarity observed in old-growth forests (Fig 2). Composition increased from an initial value of 0.19 to an asymptote of 0.72 (Table 2). Previous land use influenced recovery rates. Recovery was faster in former cocoa farms than in former mining sites for diversity, whereas land-use effects on compositional recovery were negligible (Table 2).

Fig. 2. Modelled recovery trajectories of Shannon diversity and floristic composition in secondary tropical forests of West Africa (Taï National Park, Côte d'Ivoire). Lines represent posterior mean recovery trajectories estimated from the Bayesian exponential recovery model; shaded areas indicate 95% credible intervals. Solid lines correspond to the temporal range robustly informed by the underlying 9–26 year chronosequence (up to ~40 years since disturbance); dashed lines

represent the model's extrapolation beyond this range, up to 200 years, and should be interpreted with caution given the increasing uncertainty. Recovery is expressed as the percentage of convergence toward old-growth reference conditions, with 100% corresponding to the asymptotic state observed in intact West African old-growth forests.

Table 2. Posterior summaries of the recovery model parameters for diversity and composition in secondary forests of West Africa.

Parameters	Diversity			Composition		
	Median	5th	95th	Median	5th	95th
θ_0	8.94	3.83	17.96	0.19	0.02	0.71
$\theta_{+\infty}$	20.39	16.15	24.90	0.72	0.52	0.97
λ	0.06	0.02	0.17	0.03	0.00	0.16
λ_{Cocoa}	0.03	0.01	0.08	0.01	0.00	0.03
λ_{Mining}	0.02	0.01	0.06	0.01	0.00	0.02
λ_{others}	0.08	0.02	0.18	0.02	0.00	0.06
θ_{dist}	0.51	-3.88	4.44	-0.27	-0.44	-0.17
σ	0.44	0.40	0.50	1.74	1.68	1.81

Posterior medians and 90% credible intervals (5th–95th percentiles) of the exponential recovery model parameters for Shannon diversity (Hill number of order 1) and floristic composition (abundance-based Chao–Jaccard similarity). θ_0 represents the initial value of the recovery trajectory; $\theta_{+\infty}$ the asymptotic value corresponding to old-growth reference conditions; λ the intrinsic recovery rates ; θ_{dist} the effect of past disturbance on the asymptotic value; and σ the residual variance of the log-normal likelihood. Posterior estimates of environmental covariate effects (β parameters) are not shown here and are presented in Fig. 3.

Environmental covariates had contrasting effects on recovery rates (Fig 3). Remnant trees showed positive effects on both diversity and composition. Human activities and island configuration were associated with slower recovery of both attributes. Mammal presence increased compositional recovery but was negatively associated with diversity recovery. Soil hydromorphy and disturbance exhibited variable effects, with wide credible intervals for diversity but negative effects on compositional recovery.

Fig.3. Environmental drivers of diversity and composition recovery rates. Points represent posterior mean effects of standardized covariates on the intrinsic recovery rate (λ) for diversity (x-axis) and composition (y-axis). Error bars indicate 95% credible intervals. Vertical and horizontal dashed lines represent zero effect. Positive values indicate an increase in recovery rate, whereas negative values indicate slower recovery. Covariates include mammal presence, remnant tree density, soil hydromorphy, island location, human activities, and disturbance intensity.

Functional Trait Dynamics

Trait values varied jointly with stem diameter and forest age, with distinct convergence rates among traits (Table 3).

Wood density showed a gradual increase with forest age and remained structured by stem diameter (Fig 4). Larger individuals exhibited slightly lower wood density values than smaller individuals, consistent with the negative size effect ($\beta = -0.17$). Convergence toward old-growth conditions was slow ($\lambda = 0.15$). The disturbance parameter ($\theta = 0.97$) indicated slightly lower expected values under disturbance relative to intact old-growth forests.

LMA was strongly influenced by stem diameter, with larger trees displaying higher values ($\beta = 4.63$). In contrast to wood density, LMA converged rapidly toward old-growth reference

conditions (λ = 1.40). Predicted values exceeded 60 $g.m^{-2}$ within the first decade of succession across most of the DBH gradient (Fig 4). The disturbance effect was minimal (θ = 0.98).

Seed mass increased with stem diameter (β = 1.26) and exhibited rapid convergence dynamics (λ = 1.21). Predicted seed mass values for larger individuals exceeded 25 mg within the first 10 years of succession and remained stable thereafter (Fig 4). The disturbance parameter was close to unity (θ = 0.98; 90% CI: 0.95–0.99), indicating limited deviation from intact old-growth reference values.

Table 3. Posterior summaries of the recovery model parameters for functional traits in secondary forests of West Africa.

**Parameters**	**Seed mass**			**Wood density**			**Leaf mass per area**		
	Median	5th	95th	Median	5th	95th	Median	5th	95th
α	20.43	19.89	21.01	0.61	0.61	0.62	60.12	59.63	60.60
β	1.26	0.41	2.11	-0.17	-0.18	-0.16	4.63	4.63	5.46
λ	1.21	0.72	2.25	0.15	0.15	0.16	1.4	0.9	2.47
θ	0.98	0.95	0.99	0.97	0.96	0.99	0.98	0.99	0.99
σ	2.85	2.81	2.89	0.22	0.22	0.23	3.62	3.5	3.69

Posterior medians and 90% credible intervals (5th–95th percentiles) of the individual-level exponential convergence model parameters for seed mass, wood density, and leaf mass per area. α represents the expected trait value when DBH = 0; β quantifies the effect of stem diameter on trait values; λ is the intrinsic convergence rate toward old-growth reference conditions; θ represents the multiplicative effect of disturbance on trait values in old-growth forests; and σ is the residual variance of the log-normal likelihood.

Fig. 4. Modelled trajectories of functional trait distributions along the DBH and successional age gradients in secondary forests of West Africa. Heatmaps represent posterior mean predicted values of wood density ($g.cm^{-3}$), leaf mass per area (LMA; $g.m^{-2}$), and seed mass (mg)

as functions of stem diameter (DBH, m; x-axis) and time since abandonment (years; y-axis). Color gradients indicate expected trait values derived from the individual-level convergence model. Predictions illustrate progressive alignment of trait–size relationships toward old-growth reference conditions as forest age increases.

Conservation-relevant species

Among the 174 recorded species, 16 (9.2%) were classified as either Endangered (EN) or Vulnerable (VU) according to the IUCN Red List (Appendix 1), and 44 species (25.3%) were identified as old-growth indicator species based on the IndVal analysis (Appendix 2).

IUCN-listed species were present across all forest stages. Their proportion remained relatively stable among young secondary forests (≤ 11 years), older secondary forests (> 11 years), and intact old-growth forests, with values ranging between 8% and 9% of individuals (Fig 5).

In contrast, the proportion of old-growth indicator species increased with forest age. Indicator species represented approximately 3–5% of individuals in young secondary forests, 4–6% in intermediate secondary forests, and 7–11% in old-growth forests (Fig 5).

Fig. 5. Proportion of conservation-relevant species across successional stages in West African tropical forests. Boxplots show the percentage of individuals belonging to old-growth indicator species (left panel) and IUCN-listed species categorized as Vulnerable or Endangered (right panel) across three forest categories: secondary forests ≤ 11 years since abandonment, secondary forests > 11 years, and intact old-growth forests. Boxes represent interquartile ranges, horizontal lines indicate medians, and whiskers show the range of observed values

Discussion

In the last extensive block of Upper Guinean rainforest in West Africa, our results show that secondary forests surrounding Taï National Park retain substantial ecological potential. Although biodiversity dimensions recover at different rates, species diversity rebounds relatively quickly, functional attributes re-establish within decades, and conservation-relevant species persist across successional stages. These findings provide encouraging evidence that, when protected from further disturbance, naturally regenerating forests in this region can contribute meaningfully to biodiversity conservation and landscape resilience.

Rapid diversity recovery masks slower and context-dependent compositional convergence

The rapid recovery of diversity likely reflects the resilience of secondary forests and the progressive accumulation of species following land abandonment [48]. Similar patterns have been reported in Neotropical forests, where up to 90% of species richness can recover within two decades [48–50]. In contrast, floristic composition depends on the return of late-successional species, many of which are dispersal-limited and characterized by larger seed mass [51,52]. As a result, compositional convergence may require several decades to centuries. Comparable trajectories have been observed in the Congo Basin [53], the Neotropics [54], and Southeast Asia [52], reinforcing the generality of this asynchronous recovery pattern.

Importantly, compositional recovery was not only slower but also highly variable, reflecting strong context dependence. It should be noted that the lower bound of the credible interval for the compositional recovery rate is very close to zero, meaning our data cannot exclude a near-negligible recovery rate that would be practically equivalent to stagnation over the sampled chronosequence (9–26 years); this uncertainty highlights the need for longer-term monitoring

to distinguish slow recovery from an effective stall. Previous land use played a central role. Abandoned cocoa plantations often retain partial soil fertility [55] and remnant trees [56,57], which facilitate regeneration and canopy re-establishment [58,59]. In contrast, artisanal gold mining removes topsoil, disrupts seed banks, alters soil structure, and may lead to heavy metal accumulation, reducing microbial activity and long-term fertility [16,60]. Such disturbances strongly constrain recolonization by forest specialists and slow compositional convergence [41].

Environmental factors further modulated recovery trajectories. Remnant trees emerged as a key driver accelerating both diversity and compositional recovery. These trees create favorable microhabitats, increase structural heterogeneity, and act as local seed sources [55,61]. They also attract seed dispersers such as birds and bats, facilitating the recruitment of late-successional species [62,63]. Similar patterns have been documented elsewhere, where species richness and compositional similarity to primary forests increase around remnant trees after two decades of succession [64,65]. Conversely, human activities and forest isolation slowed recovery. Hunting and logging associated with gold mining reduce populations of large seed dispersers, thereby limiting recruitment of large-seeded forest species [66,67]. Forests on islands created a fragmentation experience that reduced propagule influx and strong edge effects, favoring pioneer and generalist species [68–70]. In line with island biogeography theory [71], limited connectivity constrains long-term compositional recovery. Hydromorphic soils also exerted contrasting effects, promoting herbaceous dominance (e.g., Marantaceae) that can inhibit woody recruitment and slow structural and compositional reassembly [59,72–74].

Together, these results show that while diversity rebounds relatively quickly in regenerating forests of West Africa, floristic composition follows a slower and highly context-dependent

trajectory. Around Taï National Park, this asynchrony underscores the importance of maintaining landscape connectivity, remnant trees, and effective protection from continued disturbance to support long-term compositional recovery.

Rapid reassembly of leaf and regeneration traits contrasts with slow structural maturation

Functional traits recovered at markedly different rates, revealing distinct ecological dimensions of succession in the forests surrounding Taï National Park. In intact old-growth forests, trait distributions reflect conservative resource-use strategies adapted to closed-canopy conditions, where light is limiting and competition for water and nutrients is intense [75]. Dominant species tend to be shade tolerant and invest in durable tissues, including dense wood and structurally robust leaves.

In contrast, secondary forests initially host species that prioritize rapid growth over structural investment. The lower wood density observed in younger forests likely reflects the dominance of pioneer species that colonized soon after abandonment. These heliophilous species capitalize on high light availability created by disturbance, investing in fast height growth rather than dense, mechanically resistant wood. As succession proceeds and canopy closure increases, later-arriving species face reduced light and heightened resource competition [76]. These species typically allocate more biomass to dense wood, enhancing mechanical stability and resistance to disturbance [77]. Such ontogenetic and successional shifts in species composition likely explain the slow convergence of wood density, consistent with broader evidence that structural maturation in tropical forests requires several decades [21,78].

In contrast, leaf mass per area (LMA) reassembled rapidly. LMA increased with both forest age and stem diameter, reflecting adjustments to vertical light gradients and hydraulic constraints. Leaves positioned higher in the canopy experience greater irradiance and wind

exposure, promoting thicker and denser leaf construction [79,80]. Increased hydraulic path length in taller individuals further constrains water transport to the canopy, favoring structural reinforcement of leaves. Smaller trees and understory individuals, by contrast, operate under lower light intensity and shorter hydraulic distances, allowing thinner and lighter leaves. The rapid convergence of LMA suggests that canopy stratification and associated microclimatic gradients re-establish relatively quickly during succession. Because LMA is assigned as a fixed species-level value to every individual of a given species, rather than measured directly on individual trees, our data cannot capture in situ or ontogenetic adjustment of leaf traits within individuals. The observed convergence in LMA therefore necessarily reflects progressive species turnover toward taxa with characteristically higher LMA, not physiological or plastic adaptation of existing trees. In addition, LMA values were available for only 57% of individuals, and species lacking a trait record were excluded from this analysis; while we have no indication of systematic bias in which species were missing, this limited coverage should be kept in mind when assessing the generality of the LMA convergence pattern.

Seed mass dynamics reflect a distinct regeneration axis. Pioneer species typically produce numerous small and light seeds that disperse effectively into disturbed environments [29,81]. Although these seeds contain limited reserves, they benefit from high-light conditions and rapid germination. In contrast, shade-tolerant species produce fewer but larger seeds rich in reserves, enhancing seedling survival under low-light and competitive understory conditions [82–84]. Small-seeded species compensate for lower individual survival through high fecundity and persistence in soil seed banks [81,85]. The relatively rapid stabilization of seed mass distributions indicates that regeneration strategies diversify early in succession, even before full structural maturation of the forest.

Together, these patterns show that while leaf-level adjustments and regeneration strategies reassemble within decades, structural attributes associated with long-lived woody tissues recover more slowly. Around Taï National Park, this decoupling between rapid functional adjustment and delayed structural maturation highlights that forest recovery involves multiple ecological dimensions operating on different temporal scales.

Naturally regenerating forests contribute meaningfully to conservation

The presence of IUCN-listed species across all forest stages indicates that naturally regenerating forests around Taï National Park maintain conservation value throughout succession. The proportion of Endangered and Vulnerable species remained relatively stable across young secondary, older secondary, and old-growth forests, suggesting that early successional habitats contribute to sustaining threatened taxa. Similar complementary roles of secondary forests have been reported in other tropical landscapes [86–88].

In contrast, old-growth indicator species were markedly more abundant in intact forests than in secondary stands (Fig. 5). Although indicator species were present in both young and older secondary forests likely reflecting the persistence of remnant trees [89], their proportion remained substantially lower than in old-growth forests. This pattern suggests that while secondary forests support elements of primary forest composition, full recovery of old-growth identity requires longer time spans and sustained protection. Comparable trends have been documented in West Africa, where indicator species progressively increase but remain less abundant in secondary stands than in primary forests [73].

Together, these findings highlight the complementary but not substitutive role of secondary forests in the Upper Guinean region. While they do not yet replicate the full compositional integrity of old-growth forests, their capacity to maintain threatened species and retain primary-forest elements underscores their importance for biodiversity conservation in West Africa.

Conclusion

Following episodes of intense degradation during the post-electoral crises, the forests surrounding Taï National Park have undergone a decade of natural regeneration. Our results show that this regeneration is not merely structural [59,90] but ecologically meaningful. Secondary forests recover species diversity rapidly, reassemble key functional attributes within decades, and retain threatened taxa across successional stages. Although full compositional convergence toward old-growth forests remains slower and strongly context-dependent, the progressive return of primary-forest elements demonstrates substantial ecological resilience in the last major block of Upper Guinean rainforest. In a region where deforestation has drastically reduced forest cover, these findings provide concrete evidence that protecting regenerating forests around Taï is not a secondary objective but a central pillar of long-term conservation. Safeguarding both intact old-growth stands and naturally recovering forests is essential to maintain biodiversity, ecological functions, and landscape connectivity in West Africa.

Acknowledgments

The authors wish to thank the management of Taï National Park for their logistical and financial support, as well as the forest rangers whose assistance greatly facilitated the data collection phase

References

1. Myers N, Mittermeier RA, Mittermeier CG, da Fonseca GAB, Kent J. Biodiversity hotspots for conservation priorities. Nature. févr 2000;403(6772):853-8. doi:10.1038/35002501

2. Sechrest W, Brooks TM, da Fonseca GAB, Konstant WR, Mittermeier RA, Purvis A, et al. Hotspots and the conservation of evolutionary history. Proc Natl Acad Sci U S A. 19 févr 2002;99(4):2067-71. doi:10.1073/pnas.251680798 PubMed PMID: 11854502; PubMed Central PMCID: PMC122320.

3. Barima YSS, Kouakou ATM, Bamba I, Sangne YC, Godron M, Andrieu J, et al. Cocoa crops are destroying the forest reserves of the classified forest of Haut-Sassandra (Ivory Coast). Glob Ecol Conserv. 1 oct 2016;8:85-98. doi:10.1016/j.gecco.2016.08.009

4. Ruf F, Koné D. Climate change, cocoa migrations and deforestation in West Africa: What does the past tell us about the future. Sustain Sci. 2015;10:101-11. doi:10.1007/s11625-014-0282-4

5. Sanial E, Ruf F, Louppe D, Mietton M, Hérault B. Local farmers shape ecosystem service provisioning in West African cocoa agroforests. Agrofor Syst. mars 2023;97(3):401-14. doi:10.1007/s10457-021-00723-6

6. Depecker J, Asimonyio JA, Miteho R, Hatangi Y, Kambale JL, Verleysen L, et al. The association between rainforest disturbance and recovery, tree community composition, and community traits in the Yangambi area in the Democratic Republic of the Congo. J Trop Ecol. nov 2022;38(6):426-36. doi:10.1017/S0266467422000347

7. Chazdon RL. Tropical forest recovery: legacies of human impact and natural disturbances. Perspect Plant Ecol Evol Syst. 1 janv 2003;6(1):51-71. doi:10.1078/1433-8319-00042

8. Chazdon RL. Second Growth: The Promise of Tropical Forest Regeneration in an Age of Deforestation. In: Second Growth [Internet]. University of Chicago Press; 2014 [cité 3 mai 2025]. Disponible sur: https://www.degruyterbrill.com/document/doi/10.7208/9780226118109/html

9. FAO. Évaluation des ressources forestières mondiales [Rapport final]. Rome; 2020. p. 16 p.

10. Brancalion PHS, Niamir A, Broadbent E, Crouzeilles R, Barros FSM, Almeyda Zambrano AM, et al. Global restoration opportunities in tropical rainforest landscapes. Sci Adv. 3 juill 2019;5(7):eaav3223. doi:10.1126/sciadv.aav3223

11. Teixeira HM, Cardoso IM, Bianchi FJJA, da Cruz Silva A, Jamme D, Peña-Claros M. Linking vegetation and soil functions during secondary forest succession in the Atlantic forest. For Ecol Manag. 1 févr 2020;457:117696. doi:10.1016/j.foreco.2019.117696

12. Aide TM, Clark ML, Grau HR, López-Carr D, Levy MA, Redo D, et al. Deforestation and Reforestation of Latin America and the Caribbean (2001–2010). Biotropica. 2013;45(2):262-71. doi:10.1111/j.1744-7429.2012.00908.x

13. Arroyo-Rodríguez V, Melo FPL, Martínez-Ramos M, Bongers F, Chazdon RL, Meave JA, et al. Multiple successional pathways in human-modified tropical landscapes: new insights from forest succession, forest fragmentation and landscape ecology research: Multiple successional pathways. Biol Rev. févr 2017;92(1):326-40. doi:10.1111/brv.12231

14. Chazdon RL, Finegan B, Capers RS, Salgado-Negret B, Casanoves F, Boukili V, et al. Composition and Dynamics of Functional Groups of Trees During Tropical Forest Succession in Northeastern Costa Rica. Biotropica. 2010;42(1):31-40. doi:10.1111/j.1744-7429.2009.00566.x

15. Poorter L, Craven D, Jakovac CC, van der Sande MT, Amissah L, Bongers F, et al. Multidimensional tropical forest recovery. Science. déc 2021;374(6573):1370-6. doi:10.1126/science.abh3629

16. Peterson GD, Heemskerk M. Deforestation and forest regeneration following small-scale gold mining in the Amazon: the case of Suriname. Environ Conserv. juin 2001;28(2):117-26. doi:10.1017/S0376892901000121

17. Derroire G, Tigabu M, Odén PC, Healey JR. The Effects of Established Trees on Woody Regeneration during Secondary Succession in Tropical Dry Forests. Biotropica. mai 2016;48(3):290-300. doi:10.1111/btp.12287

18. Arroyo-Rodríguez V, PL Melo F, Martínez-Ramos M, Bongers F, L. Chazdon R, A. Meave J, et al. Multiple successional pathways in human-modified tropical landscapes: new insights from forest succession, forest fragmentation and landscape ecology research. Biol Rev [Internet]. 5 nov 2015 [cité 29 avr 2025];92(1):326-40. Disponible sur: https://onlinelibrary.wiley.com/doi/10.1111/brv.12231

19. Amani BHK, N'Guessan AE, Derroire G, N'dja JK, Elogne AGM, Traoré K, et al. The potential of secondary forests to restore biodiversity of the lost forests in semi-deciduous West Africa. Biol Conserv. juill 2021;259. doi:10.1016/j.biocon.2021.109154

20. Wright SJ, Kitajima K, Kraft NJB, Reich PB, Wright IJ, Bunker DE, et al. Functional traits and the growth–mortality trade-off in tropical trees. Ecology. 2010;91(12):3664-74. doi:10.1890/09-2335.1

21. Poorter L, Rozendaal DMA, Bongers F, de Almeida-Cortez JS, Almeyda Zambrano AM, Álvarez FS, et al. Wet and dry tropical forests show opposite successional pathways in wood density but converge over time. Nat Ecol Evol. juin 2019;3(6):928-34. doi:10.1038/s41559-019-0882-6

22. Lavorel S, Garnier E. Predicting changes in community composition and ecosystem functioning from plant traits: revisiting the Holy Grail. Funct Ecol. 2002;16(5):545-56. doi:10.1046/j.1365-2435.2002.00664.x

23. Chave J, Coomes D, Jansen S, Lewis SL, Swenson NG, Zanne AE. Towards a worldwide wood economics spectrum. Ecol Lett. 1 avr 2009;12(4):351-66. doi:10.1111/j.1461-0248.2009.01285.x

24. Poorter L. The Relationships of Wood-, Gas- and Water Fractions of Tree Stems to Performance and Life History Variation in Tropical Trees. Ann Bot. sept 2008;102(3):367-75. doi:10.1093/aob/mcn103

25. Schmitt S, Maréchaux I, Chave J, Fischer FJ, Piponiot C, Traissac S, et al. Functional diversity improves tropical forest resilience: Insights from a long-term virtual experiment. Gomez-Aparicio L, éditeur. J Ecol. mai 2020;108(3):831-43. doi:10.1111/1365-2745.13320

26. Herault B, Ouallet J, Blanc L, Wagner F, Baraloto C. Growth responses of neotropical trees to logging gaps. J Appl Ecol. 2010;47(4):821-31. doi:10.1111/j.1365-2664.2010.01826.x

27. Baraloto C, Forget PM, Goldberg DE. Seed mass, seedling size and neotropical tree seedling establishment. J Ecol. 2005;93(6):1156-66. doi:10.1111/j.1365-2745.2005.01041.x

28. Kitajima K. Do shade-tolerant tropical tree seedlings depend longer on seed reserves? Functional growth analysis of three Bignoniaceae species. Funct Ecol. 2002;16(4):433-44. doi:10.1046/j.1365-2435.2002.00641.x

29. Moles AT, Falster DS, Leishman MR, Westoby M. Small-seeded species produce more seeds per square metre of canopy per year, but not per individual per lifetime. J Ecol. 2004;92(3):384-96. doi:10.1111/j.0022-0477.2004.00880.x

30. Doua-Bi GYA, Zo-Bi IC, Amani BHK, Elogne AGM, N'dja JK, N'Guessan AE, et al. Taking advantage of natural regeneration potential in secondary forests to recover commercial tree resources in Côte d'Ivoire. For Ecol Manag. août 2021;493(May):119240-119240. doi:10.1016/j.foreco.2021.119240

31. Châtelain C, Aké-Assi L, Spichiger R, Gautier L. Cartes de distribution des plantesde Côte d'Ivoire. Conservatoire et Jardin botanique de la Ville de Genève. Vol. 64. Suisse; 2011. 327 p.

32. OIPR. Plan d'Aménagement et de Gestion (PAG) du Parc national de Taï 2014-2018. [Rapport d'aménagement]. 2014. p. 131 p.

33. OIPR. Plan d'Aménagement et de Gestion du Parc national de Taï (PAG) 2020-2029 [Rapport stratégique de planification environnementale]. Abidjan, Côte d'Ivoire; 2020. p. 208.

34. Kouamélan E.P. Biodiversité des poissons du Parc national de Taï : Une ressource naturelle à sauvegarder. Éditionns Universitaires Européennes. In. Abidjan (Côted'Ivoire); 2018. p. 165 p.

35. Van Rompaey R, Adou Yao C, Déngueadhé KBE, Aké-Assi L, Wittebolle G, N'Guessan K. Biodiversité végétale et écologie du paysage dans le Sud du Parc national

deTaï (Côte d'Ivoire) [Rapport de synthèse de projet 04/97-1111a du Programme Tropenbos Côte d'Ivoire]. Wageningen, Pays-Bas; 2001. p. 42 p.

36. Bakayoko A, Neuba DFR, Kouadio YL, Nehoun B. Suivi de la restauration végétale naturelle des zones aménagées dans les secteurs de Djapadji, Soubré et ADK/V6 du Parc national de Taï. [Rapport de suivi des zones récupérées]. Abidjan (Côte d'Ivoire); 2018. p. 24 p.

37. Aké-Assi L. Flore de la Côte-d'Ivoire: Catalogue Systématique, Biogéographie et Écologie. 2001. (Boissiera 57–58. Conservatoire et Jardin botanique de la Ville de Genève).

38. Hutchinson J, Dalziel JM. Flora of West Tropical Africa. 2nd ed. Keay RWJ, Hepper FN, editors. Vol I–III. London: Crown Agents for Overseas Governments and Administrations. 1954–1972 p.

39. Réjou-Méchain M, Tanguy A, Piponiot C, Chave J, Hérault B. biomass: an r package for estimating above-ground biomass and its uncertainty in tropical forests. Methods Ecol Evol. sept 2017;8(9):1163-7. doi:10.1111/2041-210X.12753

40. De La Mensbruge G. La germination et les plantules des essences arborées de la forêt dense humide de la Côte D'Ivoire. CTFT. 1966. 389 p.

41. Dufrêne M, Legendre P. Species Assemblages and Indicator Species:the Need for a Flexible Asymmetrical Approach. Ecol Monogr. 1997;67(3):345-66. doi:10.1890/0012-9615(1997)067[0345:SAAIST]2.0.CO;2

42. Marcon E, Hérault B. entropart: An R Package to Measure and Partition Diversity. J Stat Softw. 7 oct 2015;67:1-26. doi:10.18637/jss.v067.i08

43. Maurent E, Hérault B, Piponiot C, Derroire G, Delgado D, Finegan B, et al. A common framework to model recovery in disturbed tropical forests. Ecol Model. sept 2023;483:110418-110418. doi:10.1016/J.ECOLMODEL.2023.110418

44. Carpenter B, Gelman A, Hoffman MD, Lee D, Goodrich B, Betancourt M, et al. Stan: A Probabilistic Programming Language. J Stat Softw. 11 janv 2017;76:1-32. doi:10.18637/jss.v076.i01

45. Hérault B, Piponiot C. Key drivers of ecosystem recovery after disturbance in a neotropical forest: Long-term lessons from the Paracou experiment, French Guiana. For Ecosyst. déc 2018;5(1):2. doi:10.1186/s40663-017-0126-7

46. Barros TC, Elias F, Romano LL, Ferreira J. Natural recovery of plant species diversity in secondary forests in Eastern Amazonia: contributions to passive forest restoration. Braz J Bot. 1 mars 2020;43(1):165-75. doi:10.1007/s40415-020-00585-9

47. Chazdon RL, Letcher SG, Van Breugel M, Martínez-Ramos M, Bongers F, Finegan B. Rates of change in tree communities of secondary Neotropical forests following major disturbances. Philos Trans R Soc B Biol Sci. févr 2007;362(1478):273-89. doi:10.1098/rstb.2006.1990

48. Lennox GD, Gardner TA, Thomson JR, Ferreira J, Berenguer E, Lees AC, et al. Second rate or a second chance? Assessing biomass and biodiversity recovery in regenerating Amazonian forests. Glob Change Biol. 2018;24(12):5680-94. doi:10.1111/gcb.14443

49. Chazdon RL, Norden N, Colwell RK, Chao A. Monitoring recovery of tree diversity during tropical forest restoration: lessons from long-term trajectories of natural regeneration. Philos Trans R Soc B Biol Sci. 14 nov 2022;378(1867):20210069. doi:10.1098/rstb.2021.0069

50. Deng F, He Y, Zang R, Deng F, He Y, Zang R. Recovery of Functional Diversity Following Shifting Cultivation in Tropical Monsoon Forests. Forests. 2018;9(9):1-20. doi:10.3390/f9090506

51. Makelele IA, Verheyen K, Boeckx P, Cizungu Ntaboba L, Mujinya Bazirake B, Ewango C, et al. Afrotropical secondary forests exhibit fast diversity and functional recovery, but slow compositional and carbon recovery after shifting cultivation. Carlucci M, éditeur. J Veg Sci. sept 2021;32(5):e13071. doi:10.1111/jvs.13071

52. Rozendaal DMA, Bongers F, Aide TM, Alvarez-Dávila E, Ascarrunz N, Balvanera P, et al. Biodiversity recovery of Neotropical secondary forests. Sci Adv. mars 2019;5(3):eaau3114. doi:10.1126/sciadv.aau3114

53. Koffi A.B., Tondoh E.J., Ehouman E., Freycon V., Piponiot C., Amani B.H.K, et al. Drivers of soil organic carbon recovery during secondary succession along a West African climate gradient. Ecol Indic. 2025;178:1-11. doi:10.1016/j.ecolind.2025.114073

54. Konan IK, N'Guessan AE, Dago MR, Kouassi A, N'dja JK, Aussenac R, et al. Maximizing tree diversity in cocoa agroforestry: taking advantage of planted, spontaneous, and remnant trees. Agrofor Syst. 2025;99(5):1-14. doi:10.1007/s10457-025-01180-1

55. Kouassi AK, Zo-Bi IC, Aussenac R, Kouamé IK, Dago MR, N'guessan AE, et al. The great mistake of plantation programs in cocoa agroforests – Let's bet on natural regeneration to sustainably provide timber wood. Trees For People. 1 juin 2023;12:100386. doi:10.1016/j.tfp.2023.100386

56. N'Guessan AE, Hérault B, Zo-Bi IC, Aussenac R, Dago MR, Konan IK, et al. Rethinking cocoa agroforestry: towards sustainable coexistence of remnant, spontaneous and planted trees. Perspective. 20 janv 2026;(70):1-4. Located at: Côte d'Ivoire, West Africa. doi:10.19182/perspective/38076

57. Houphouët ADL, Sangne YC, Diarrassouba A, Adou Yao YC, Betbeder J, Hérault B. Forest structure recovery around West Africa's last great rainforest: Modelling complex dynamics in Taï national park. Trees For People. déc 2025;22:1-12. doi:10.1016/j.tfp.2025.101005

58. Albert KM. Role of revegetation in restoring fertility of degraded mined soils in Ghana: A review. Int J Biodivers Conserv. 28 févr 2015;7(2):57-80. doi:10.5897/IJBC2014.0775

59. Pérez-Cárdenas N, Mora F, Arreola-Villa F, Arroyo-Rodríguez V, Balvanera P, Flores-Casas R, et al. Effects of landscape composition and site land-use intensity on secondary

succession in a tropical dry forest. For Ecol Manag. 15 févr 2021;482:118818. doi:10.1016/j.foreco.2020.118818

60. Tucker MM, Kashian DM. Pre-fire forest remnants affect post-fire plant community structure and composition. For Ecol Manag. janv 2018;408:103-11. doi:10.1016/J.FORECO.2017.10.038

61. Wu MM, Liang Y, He HS, Liu B, Ma T. Remnant trees location and abundance play different roles in forest landscape recovery. For Ecol Manag. 1 mai 2022;511:120154. doi:10.1016/j.foreco.2022.120154

62. Sandor ME, Chazdon RL. Remnant Trees Affect Species Composition but Not Structure of Tropical Second-Growth Forest. Farwig N, éditeur. PLoS ONE. 13 janv 2014;9(1):e83284. doi:10.1371/journal.pone.0083284

63. Sterck FJ, Poorter L, Schieving F. Leaf traits determine the growth-survival trade-off across rain forest tree species. Am Nat. mai 2006;167(5):758-65. doi:10.1086/503056

64. Brodie JF, Helmy OE, Brockelman WY, Maron JL. Bushmeat poaching reduces the seed dispersal and population growth rate of a mammal-dispersed tree. Ecol Appl. 2009;19(4):854-63. doi:10.1890/08-0955.1

65. Markl JS, Schleuning M, Forget PM, Jordano P, Lambert JE, Traveset A, et al. Meta-Analysis of the Effects of Human Disturbance on Seed Dispersal by Animals. Conserv Biol. 2012;26(6):1072-81. doi:10.1111/j.1523-1739.2012.01927.x

66. Laurance WF. Theory meets reality: How habitat fragmentation research has transcended island biogeographic theory. Biol Conserv. 1 juill 2008;141(7):1731-44. doi:10.1016/j.biocon.2008.05.011

67. Murcia C. Edge effects in fragmented forests: implications for conservation. Trends Ecol Evol. 1 févr 1995;10(2):58-62. doi:10.1016/S0169-5347(00)88977-6

68. Traoré S, Zo-Bi IC, Piponiot C, Aussenac R, Hérault B. Fragmentation is the main driver of residual forest aboveground biomass in West African low forest-high deforestation landscapes. Trees For People. 1 mars 2024;15:100477. doi:10.1016/j.tfp.2023.100477

69. Cuni-Sanchez A, White LJT, Calders K, Jeffery KJ, Abernethy K, Burt A, et al. African Savanna-Forest Boundary Dynamics: A 20-Year Study. Gomory D, éditeur. PLOS ONE. 2016;11(6):156-934. doi:10.1371/journal.pone.0156934

70. Amani BHK, N'Guessan AE, Van der Meersch V, Derroire G, Piponiot C, Elogne AGM, et al. Lessons from a regional analysis of forest recovery trajectories in West Africa. Environ Res Lett. nov 2022;17(11). doi:10.1088/1748-9326/ac9b4f

71. Pouteau R, Picard J, Doumenge C, Brncic T, Gillet JF, Doucet JL, et al. The puzzling ecology of African Marantaceae forests. Am J Bot. 2024;111(4):e16320. doi:10.1002/ajb2.16320

72. Poorter L, Rozendaal DMA, Bongers F, Almeida DJS, Álvarez FS, Andrade JL, et al. Functional recovery of secondary tropical forests. Proc Natl Acad Sci. déc 2021;118(49):e2003405118-e2003405118. doi:10.1073/pnas.2003405118

73. Liang J, Crowther TW, Picard N, Wiser S, Zhou M, Alberti G, et al. Positive biodiversity-productivity relationship predominant in global forests. Science. oct 2016;354(6309). doi:10.1126/science.aaf8957

74. Hérault B, Bachelot B, Poorter L, Rossi V, Bongers F, Chave J, et al. Functional traits shape ontogenetic growth trajectories of rain forest tree species. J Ecol. nov 2011;99(6):1431-40. doi:10.1111/j.1365-2745.2011.01883.x

75. Norden N, Chazdon RL, Chao A, Jiang YH, Vílchez-Alvarado B. Resilience of tropical rain forests: tree community reassembly in secondary forests. Ecol Lett. mai 2009;12(5):385-94. doi:10.1111/j.1461-0248.2009.01292.x

76. Cavaleri MA, Oberbauer SF, Clark DB, Clark DA, Ryan MG. Height is more important than light in determining leaf morphology in a tropical forest. Ecology. juin 2010;91(6):1730-9. doi:10.1890/09-1326.1 PubMed PMID: 20583714.

77. Coble AP, Cavaleri MA. Light acclimation optimizes leaf functional traits despite height-related constraints in a canopy shading experiment. Oecologia. 1 avr 2015;177(4):1131-43. doi:10.1007/s00442-015-3219-4

78. Dalling JW, Hubbell SP, Silvera K. Seed dispersal, seedling establishment and gap partitioning among tropical pioneer trees. J Ecol. 1998;86(4):674-89. doi:10.1046/j.1365-2745.1998.00298.x

79. Peña-Claros M, Boo HD. The effect of forest successional stage on seed removal of tropical rain forest tree species. J Trop Ecol. mars 2002;18(2):261-74. doi:10.1017/S0266467402002171

80. Poorter L, Rose SA. Light-dependent changes in the relationship between seed mass and seedling traits: a meta-analysis for rain forest tree species. Oecologia. 1 janv 2005;142(3):378-87. doi:10.1007/s00442-004-1732-y

81. Konaté I, Ehouman E, Wourro F, Doua-Bi Y, Tiéoulé F, Coulibaly B, et al. The anatomy of survival — What determines seedling fate after planting in West Africa? For Ecol Manag. 15 déc 2025;598:123266. doi:10.1016/j.foreco.2025.123266

82. Muller-Landau HC. The tolerance–fecundity trade-off and the maintenance of diversity in seed size. Proc Natl Acad Sci U S A. 2 mars 2010;107(9):4242-7. doi:10.1073/pnas.0911637107 PubMed PMID: 20160078; PubMed Central PMCID: PMC2840174.

83. Banks-Leite C, Ewers RM, Kapos V, Martensen AC, Metzger JP. Comparing species and measures of landscape structure as indicators of conservation importance. J Appl Ecol. 2011;48(3):706-14. doi:10.1111/j.1365-2664.2011.01966.x

84. Barlow J, Gardner T, Araujo I, Ávila-Pires TC, Bonaldo AB, Costa JE, et al. Quantifying the biodiversity value of tropical primary, secondary and plantation forests. Proc Natl Acad Sci. 20 nov 2007;104(47):18555-60. doi:10.1073/pnas.0703333104

85. Swanson ME, Franklin JF, Beschta RL, Crisafulli CM, DellaSala DA, Hutto RL, et al. The forgotten stage of forest succession: early-successional ecosystems on forest sites. Front Ecol Environ. 2011;9(2):117-25. doi:10.1890/090157

86. Seidl R, Rammer W, Spies TA. Disturbance legacies increase the resilience of forest ecosystem structure, composition, and functioning. Ecol Appl. 2014;24(8):2063-77. doi:10.1890/14-0255.1

87. Anny Estelle N'Guessan, N'dja JK, Yao ON, Amani BHK, Gouli RGZ, Piponiot C, et al. Drivers of biomass recovery in a secondary forested landscape of West Africa. For Ecol Manag. févr 2019;433:325-31. doi:10.1016/j.foreco.2018.11.021

Supporting information

Appendix S1. IUCN-listed tree species recorded in Taï National Park (Côte d'Ivoire). Species classified as Endangered (EN) or Vulnerable (VU) according to the IUCN Red List. Values of 1 indicate the assigned conservation category

Appendix S2. Old-growth indicator species identified using the Dufrêne–Legendre indicator value method. Indicator species associated with intact old-growth forests, identified using the Dufrêne–Legendre indicator value (IndVal) method. Only species with significant association ($P < 0.002$) are reported. IndVal values range from 0 (no association) to 1 (perfect specificity and fidelity to old-growth forests).

Response to Reviewers
PLOS ONE

PONE-D-26-18781
July 27th 2026

Contents

1 Comment from the Editor 1

2 Comments from Reviewer 1 2
2.1 L128-134: Temporal Scale Adjustment . . . 2
2.2 L328-329: Uncertainty in Compositional Recovery . . . 3
2.3 L165-167: Functional Traits and Imputation . . . 3
2.4 L147-148: Taxonomic Documentation . . . 4
2.5 L111-113: Representativeness of Reference Sites . . . 4

---

1 Comment from the Editor

> *Dear authors, We have received the review of your manuscript. The reviewer commends the immense effort dedicated to your field data collection and acknowledges that your dataset represents a valuable contribution to West African forest ecology, particularly given the integration of taxonomic, functional, and compositional dimensions via a Bayesian framework. Based on the reviewer's comments, I have reached a decision of Major Revision. While the manuscript shows significant promise, there are several methodological and interpretive concerns that must be addressed to ensure the rigor of your conclusions. Please pay close attention to the following points when preparing your revised submission*

We thank the Editor for the careful assessment of our manuscript and for the opportunity to revise it. In the following our responses to comments are in grey and changes in the text are reported in red.

> *Temporal Extrapolation: Your models currently project trajectories up to 200 years based on a 9–26 year chronosequence. This creates a risk of speculative inference. Please restrict the figures and discussion to the effectively observed temporal horizon (approximately 30 years) to maintain the robustness of your findings.*

As detailed in our response to Reviewer 1 (comment 2.1 below), rather than removing the long-term projection we revised Fig. 2 to visually distinguish — via solid vs. dashed lines — the temporal range robustly informed by our chronosequence (40 years) from the extrapolated portion beyond it (up to 200 years), and revised the corresponding Results text accordingly.

> *Statistical Uncertainty: The 90% credible interval for the recovery rate (lambda) of floristic composition includes 0.00. Please provide a more transparent and cautious discussion regarding the possibility of stagnation in recovery, rather than presenting a categorical claim of convergence.*

As detailed in our response to Reviewer 1 (comment 2.2 below), we clarify that the lower bound of the 90% credible interval for the compositional recovery rate is very close to zero — though not literally zero, given the model's strictly positive exponential link for lambda — and have revised the Results and Discussion to explicitly acknowledge that a near-negligible, practically stagnant recovery rate cannot be excluded.

> *Methodological Transparency & Limitations: Trait Imputation: Given that 43% of the Leaf Mass per Area (LMA) data was imputed using global averages, please include a more robust discussion/caution regarding the interpretation of "rapid convergence." Clarify whether this reflects in situ adaptation or an artifact of the imputation.*

As detailed in our response to Reviewer 1 (comment 2.3 below), we clarify that LMA (like wood density and seed mass) is assigned as a fixed species-level value rather than measured on individual trees or imputed from global averages for missing individuals; the rapid convergence observed therefore necessarily reflects species turnover rather than in situ adaptation. We have also flagged the 57% trait coverage as an explicit limitation on the generality of this result.

> *Voucher Specimens: As vouchers were not deposited, please provide detailed information on the specific regional floras or keys utilized for species identification to ensure the reproducibility of your inventory.*

As detailed in our response to Reviewer 1 (comment 2.4 below), we have added explicit reference to the regional floras used for species identification — Aké-Assi's Flore de la Côte-d'Ivoire and Hutchinson & Dalziel's Flora of West Tropical Africa — to the Methods section, to improve the reproducibility of our inventory.

2 Comments from Reviewer 1

> *Dear Authors, I would like to congratulate you on your work. The immense effort dedicated to field data collection in the Taï National Park—a region of inestimable ecological importance is clearly evident. Using a Bayesian framework to integrate taxonomic, functional, and compositional dimensions is a valuable and welcome contribution to West African forest ecology. I am a strong believer in collaborative science and see great potential in this manuscript. To ensure the work meets the technical rigor required, I suggest a few adjustments that will strengthen the foundation of your conclusions:*

We sincerely thank Reviewer 1 for their positive assessment of our manuscript and for their constructive and detailed suggestions. We address each point below and have revised the manuscript accordingly to strengthen the rigor of our conclusions.

2.1 L128-134: Temporal Scale Adjustment

> *The study presents a solid dataset from a 9 to 26-year chronosequence. However, the models project trajectories up to 200 years. I kindly suggest that the figures and discussions be restricted to the effectively observed temporal horizon (approximately*

30 years). Such long-term projections can introduce uncertainties that may obscure the real, robust results you have already obtained.

We thank the reviewer for raising this important point. Rather than removing the long-term projection entirely, we chose to retain it for context while visually distinguishing the robust, data-informed portion of the trajectory from the extrapolated one. Fig. 2 has been revised so that recovery trajectories are shown as solid lines up to 40 years since disturbance — the horizon within which the model is most tightly constrained by the underlying 9–26 year chronosequence — and as dashed lines beyond 40 years, up to 200 years, to explicitly flag the increasingly speculative nature of the projection. The 95% credible interval is shown throughout and widens accordingly in the extrapolated segment. We have also revised the Results and Discussion text to base our interpretive claims only on the solid-line (robust) portion of the trajectories, treating the dashed segment as illustrative only.

In the text: Lines represent posterior mean recovery trajectories estimated from the Bayesian exponential recovery model; shaded areas indicate 95% credible intervals. Solid lines correspond to the temporal range robustly informed by the underlying 9–26 year chronosequence (up to 40 years since disturbance); dashed lines represent the model's extrapolation beyond this range, up to 200 years, and should be interpreted with caution given the increasing uncertainty. Recovery is expressed as the percentage of convergence toward old-growth reference conditions, with 100% corresponding to the asymptotic state observed in intact West African old-growth forests.

2.2 L328-329: Uncertainty in Compositional Recovery

I noted that the recovery rate () for floristic composition has a 90% credible interval starting at 0.00. This suggests that, statistically, recovery might be in a state of stagnation during the sampled period. A more transparent discussion of this uncertainty would help the reader grasp the complexity of local forest succession.

We agree with the reviewer that the lower bound of the 90% credible interval for the compositional recovery rate is very close to zero (rounding to 0.00). Because lambda is modelled through a strictly positive exponential link function (Eq. 4), the interval does not literally include zero, but its lower bound indicates that a near-negligible recovery rate — practically indistinguishable from stagnation — cannot be excluded. We have revised the Results and Discussion to state this more precisely and to temper our interpretive claims accordingly.

In the Results: Because λ is constrained to be strictly positive by the model's exponential link function, this interval does not literally include zero; however, its lower bound is very close to zero, indicating that a near-negligible, practically stagnant recovery rate cannot be excluded.

In the Discussion: It should be noted that the lower bound of the credible interval for the compositional recovery rate is very close to zero, meaning our data cannot exclude a near-negligible recovery rate that would be practically equivalent to stagnation over the sampled chronosequence (9–26 years); this uncertainty highlights the need for longer-term monitoring to distinguish slow recovery from an effective stall.

2.3 L165-167: Functional Traits and Imputation

The 57% coverage for the LMA (leaf mass per area) trait warrants some caution. Since TRY database values and taxonomic averages were used to fill gaps, the rapid convergence observed in LMA might reflect species turnover rather than in situ functional adaptation.

We would like to clarify a point that may have led to some ambiguity in the reviewer's comment. LMA (like wood density and seed mass) is assigned as a fixed species-level value to every individual of a given species — it is not a trait measured directly on individual trees in situ, and no repeated measurements were taken on the same individuals across time. Because of this, our data structurally cannot capture any within-species or ontogenetic plasticity in leaf traits: the observed convergence in community-average LMA along the successional gradient cannot reflect in situ functional adaptation, by construction. It necessarily reflects a shift in species composition (species turnover) toward taxa with characteristically higher LMA. We also acknowledge, as an additional limitation, that LMA values were available for only 57% of individuals, with species lacking trait records excluded from the analysis; although we have no indication of systematic bias in this respect, this limited coverage constrains the generality of the LMA convergence pattern. We have rephrased the Discussion to state the turnover interpretation unambiguously and to explicitly flag this coverage limitation.
In the text: Because LMA is assigned as a fixed species-level value to every individual of a given species, rather than measured directly on individual trees, our data cannot capture in situ or ontogenetic adjustment of leaf traits within individuals. The observed convergence in LMA therefore necessarily reflects progressive species turnover toward taxa with characteristically higher LMA, not physiological or plastic adaptation of existing trees. In addition, LMA values were available for only 57% of individuals, and species lacking a trait record were excluded from this analysis; while we have no indication of systematic bias in which species were missing, this limited coverage should be kept in mind when assessing the generality of the LMA convergence pattern.

2.4 L147-148: Taxonomic Documentation

> *Identifying 174 species is an impressive feat. As voucher specimens were not deposited in herbaria, it would be valuable to provide more detail on the specific regional floras or keys used to ensure the reproducibility of the inventory.*

We thank the reviewer for this suggestion. Species identification in the field was based on the regional floras of Aké-Assi (Flore de la Côte-d'Ivoire: catalogue systématique, biogéographie et écologie) and Hutchinson & Dalziel (Flora of West Tropical Africa), which were used both for direct field identification and for verifying uncertain specimens. We have added these specific references to the Methods to improve the reproducibility of our inventory.
In the text: Species were identified in the field by experienced botanists. When identification was uncertain, specimens were examined using the regional floras of Aké-Assi [2002] and Hutchinson & Dalziel [1972] to ensure accurate determination.

2.5 L111-113: Representativeness of Reference Sites

> *The forest's "target" state is defined by 15 primary forest plots. Given that the park covers over 5,000 km^2, it would be insightful to briefly comment on whether these plots adequately capture the natural variability of such a vast ecosystem.*

We thank the reviewer for this thoughtful comment. We agree that 15 primary forest plots represent a modest sample relative to the park's total area (5,360 km2). To partly address this, plots were deliberately distributed across four sectors of the park rather than concentrated in a single location (Fig. 1), capturing some of its spatial heterogeneity. Importantly, our framework does not treat old-growth forest as a single fixed reference value: we explicitly quantified

the pairwise compositional similarity among the 15 old-growth plots themselves (OGF–OGF Chao–Jaccard similarity), which characterises the natural variability of intact-forest composition and is propagated into the model as the benchmark (and its associated uncertainty) against which secondary forests are assessed, rather than assuming a single homogeneous target state. We have added a sentence to the Methods clarifying this point and acknowledging the sampling limitation explicitly.

In the text: Although the 15 reference plots are modest relative to the park's total area, they were distributed across four sectors of the park to capture part of its spatial heterogeneity, and their pairwise similarity was used to explicitly characterise — rather than assume — the natural variability of old-growth forest composition used as the recovery benchmark.
