# Supplementary material for: Regenerating forests around Taï National Park sustain biodiversity despite constrained floristic recovery
Source: PLoS One. 2026 Oct 21;21(10):e0356900. doi: 10.1371/journal.pone.0356900 (PMC13686277; doi:10.1371/journal.pone.0356900)
Supplement: Supplementary file 2 [file pone.0356900.s005.pdf]

Response to Reviewers **PONE-D-26-18781**
PLOS ONE *July 27th 2026*

Contents

1 Comment from the Editor **1**

2 Comments from Reviewer 1 **2**
- 2.1 L128-134: Temporal Scale Adjustment 2
- 2.2 L328-329: Uncertainty in Compositional Recovery 3
- 2.3 L165-167: Functional Traits and Imputation 3
- 2.4 L147-148: Taxonomic Documentation 4
- 2.5 L111-113: Representativeness of Reference Sites 4

---

1 Comment from the Editor

> *Dear authors, We have received the review of your manuscript. The reviewer commends the immense effort dedicated to your field data collection and acknowledges that your dataset represents a valuable contribution to West African forest ecology, particularly given the integration of taxonomic, functional, and compositional dimensions via a Bayesian framework. Based on the reviewer's comments, I have reached a decision of Major Revision. While the manuscript shows significant promise, there are several methodological and interpretive concerns that must be addressed to ensure the rigor of your conclusions. Please pay close attention to the following points when preparing your revised submission*

We thank the Editor for the careful assessment of our manuscript and for the opportunity to revise it. In the following our responses to comments are in grey and changes in the text are reported in red.

> *Temporal Extrapolation: Your models currently project trajectories up to 200 years based on a 9–26 year chronosequence. This creates a risk of speculative inference. Please restrict the figures and discussion to the effectively observed temporal horizon (approximately 30 years) to maintain the robustness of your findings.*

As detailed in our response to Reviewer 1 (comment 2.1 below), rather than removing the long-term projection we revised Fig. 2 to visually distinguish — via solid vs. dashed lines — the temporal range robustly informed by our chronosequence (40 years) from the extrapolated portion beyond it (up to 200 years), and revised the corresponding Results text accordingly.

> *Statistical Uncertainty: The 90% credible interval for the recovery rate (lambda) of floristic composition includes 0.00. Please provide a more transparent and cautious discussion regarding the possibility of stagnation in recovery, rather than presenting a categorical claim of convergence.*

As detailed in our response to Reviewer 1 (comment 2.2 below), we clarify that the lower bound of the 90% credible interval for the compositional recovery rate is very close to zero — though not literally zero, given the model's strictly positive exponential link for lambda — and have revised the Results and Discussion to explicitly acknowledge that a near-negligible, practically stagnant recovery rate cannot be excluded.

> *Methodological Transparency & Limitations: Trait Imputation: Given that 43% of the Leaf Mass per Area (LMA) data was imputed using global averages, please include a more robust discussion/caution regarding the interpretation of "rapid convergence." Clarify whether this reflects in situ adaptation or an artifact of the imputation.*

As detailed in our response to Reviewer 1 (comment 2.3 below), we clarify that LMA (like wood density and seed mass) is assigned as a fixed species-level value rather than measured on individual trees or imputed from global averages for missing individuals; the rapid convergence observed therefore necessarily reflects species turnover rather than in situ adaptation. We have also flagged the 57% trait coverage as an explicit limitation on the generality of this result.

> *Voucher Specimens: As vouchers were not deposited, please provide detailed information on the specific regional floras or keys utilized for species identification to ensure the reproducibility of your inventory.*

As detailed in our response to Reviewer 1 (comment 2.4 below), we have added explicit reference to the regional floras used for species identification — Aké-Assi's Flore de la Côte-d'Ivoire and Hutchinson & Dalziel's Flora of West Tropical Africa — to the Methods section, to improve the reproducibility of our inventory.

2 Comments from Reviewer 1

> *Dear Authors, I would like to congratulate you on your work. The immense effort dedicated to field data collection in the Taï National Park—a region of inestimable ecological importance is clearly evident. Using a Bayesian framework to integrate taxonomic, functional, and compositional dimensions is a valuable and welcome contribution to West African forest ecology. I am a strong believer in collaborative science and see great potential in this manuscript. To ensure the work meets the technical rigor required, I suggest a few adjustments that will strengthen the foundation of your conclusions:*

We sincerely thank Reviewer 1 for their positive assessment of our manuscript and for their constructive and detailed suggestions. We address each point below and have revised the manuscript accordingly to strengthen the rigor of our conclusions.

2.1 L128-134: Temporal Scale Adjustment

> *The study presents a solid dataset from a 9 to 26-year chronosequence. However, the models project trajectories up to 200 years. I kindly suggest that the figures and discussions be restricted to the effectively observed temporal horizon (approximately*

30 years). Such long-term projections can introduce uncertainties that may obscure the real, robust results you have already obtained.

We thank the reviewer for raising this important point. Rather than removing the long-term projection entirely, we chose to retain it for context while visually distinguishing the robust, data-informed portion of the trajectory from the extrapolated one. Fig. 2 has been revised so that recovery trajectories are shown as solid lines up to 40 years since disturbance — the horizon within which the model is most tightly constrained by the underlying 9–26 year chronosequence — and as dashed lines beyond 40 years, up to 200 years, to explicitly flag the increasingly speculative nature of the projection. The 95% credible interval is shown throughout and widens accordingly in the extrapolated segment. We have also revised the Results and Discussion text to base our interpretive claims only on the solid-line (robust) portion of the trajectories, treating the dashed segment as illustrative only.
In the text: Lines represent posterior mean recovery trajectories estimated from the Bayesian exponential recovery model; shaded areas indicate 95% credible intervals. Solid lines correspond to the temporal range robustly informed by the underlying 9–26 year chronosequence (up to 40 years since disturbance); dashed lines represent the model's extrapolation beyond this range, up to 200 years, and should be interpreted with caution given the increasing uncertainty. Recovery is expressed as the percentage of convergence toward old-growth reference conditions, with 100% corresponding to the asymptotic state observed in intact West African old-growth forests.

2.2 L328-329: Uncertainty in Compositional Recovery

I noted that the recovery rate () for floristic composition has a 90% credible interval starting at 0.00. This suggests that, statistically, recovery might be in a state of stagnation during the sampled period. A more transparent discussion of this uncertainty would help the reader grasp the complexity of local forest succession.

We agree with the reviewer that the lower bound of the 90% credible interval for the compositional recovery rate is very close to zero (rounding to 0.00). Because lambda is modelled through a strictly positive exponential link function (Eq. 4), the interval does not literally include zero, but its lower bound indicates that a near-negligible recovery rate — practically indistinguishable from stagnation — cannot be excluded. We have revised the Results and Discussion to state this more precisely and to temper our interpretive claims accordingly.
In the Results: Because λ is constrained to be strictly positive by the model's exponential link function, this interval does not literally include zero; however, its lower bound is very close to zero, indicating that a near-negligible, practically stagnant recovery rate cannot be excluded.
In the Discussion: It should be noted that the lower bound of the credible interval for the compositional recovery rate is very close to zero, meaning our data cannot exclude a near-negligible recovery rate that would be practically equivalent to stagnation over the sampled chronosequence (9–26 years); this uncertainty highlights the need for longer-term monitoring to distinguish slow recovery from an effective stall.

2.3 L165-167: Functional Traits and Imputation

The 57% coverage for the LMA (leaf mass per area) trait warrants some caution. Since TRY database values and taxonomic averages were used to fill gaps, the rapid convergence observed in LMA might reflect species turnover rather than in situ functional adaptation.

We would like to clarify a point that may have led to some ambiguity in the reviewer's comment. LMA (like wood density and seed mass) is assigned as a fixed species-level value to every individual of a given species — it is not a trait measured directly on individual trees in situ, and no repeated measurements were taken on the same individuals across time. Because of this, our data structurally cannot capture any within-species or ontogenetic plasticity in leaf traits: the observed convergence in community-average LMA along the successional gradient cannot reflect in situ functional adaptation, by construction. It necessarily reflects a shift in species composition (species turnover) toward taxa with characteristically higher LMA. We also acknowledge, as an additional limitation, that LMA values were available for only 57% of individuals, with species lacking trait records excluded from the analysis; although we have no indication of systematic bias in this respect, this limited coverage constrains the generality of the LMA convergence pattern. We have rephrased the Discussion to state the turnover interpretation unambiguously and to explicitly flag this coverage limitation.
In the text: Because LMA is assigned as a fixed species-level value to every individual of a given species, rather than measured directly on individual trees, our data cannot capture in situ or ontogenetic adjustment of leaf traits within individuals. The observed convergence in LMA therefore necessarily reflects progressive species turnover toward taxa with characteristically higher LMA, not physiological or plastic adaptation of existing trees. In addition, LMA values were available for only 57% of individuals, and species lacking a trait record were excluded from this analysis; while we have no indication of systematic bias in which species were missing, this limited coverage should be kept in mind when assessing the generality of the LMA convergence pattern.

2.4 L147-148: Taxonomic Documentation

Identifying 174 species is an impressive feat. As voucher specimens were not deposited in herbaria, it would be valuable to provide more detail on the specific regional floras or keys used to ensure the reproducibility of the inventory.

We thank the reviewer for this suggestion. Species identification in the field was based on the regional floras of Aké-Assi (Flore de la Côte-d'Ivoire: catalogue systématique, biogéographie et écologie) and Hutchinson & Dalziel (Flora of West Tropical Africa), which were used both for direct field identification and for verifying uncertain specimens. We have added these specific references to the Methods to improve the reproducibility of our inventory.
In the text: Species were identified in the field by experienced botanists. When identification was uncertain, specimens were examined using the regional floras of Aké-Assi [2002] and Hutchinson & Dalziel [1972] to ensure accurate determination.

2.5 L111-113: Representativeness of Reference Sites

The forest's "target" state is defined by 15 primary forest plots. Given that the park covers over 5,000 km^2, it would be insightful to briefly comment on whether these plots adequately capture the natural variability of such a vast ecosystem.

We thank the reviewer for this thoughtful comment. We agree that 15 primary forest plots represent a modest sample relative to the park's total area (5,360 km2). To partly address this, plots were deliberately distributed across four sectors of the park rather than concentrated in a single location (Fig. 1), capturing some of its spatial heterogeneity. Importantly, our framework does not treat old-growth forest as a single fixed reference value: we explicitly quantified

the pairwise compositional similarity among the 15 old-growth plots themselves (OGF–OGF Chao–Jaccard similarity), which characterises the natural variability of intact-forest composition and is propagated into the model as the benchmark (and its associated uncertainty) against which secondary forests are assessed, rather than assuming a single homogeneous target state. We have added a sentence to the Methods clarifying this point and acknowledging the sampling limitation explicitly.

In the text: Although the 15 reference plots are modest relative to the park's total area, they were distributed across four sectors of the park to capture part of its spatial heterogeneity, and their pairwise similarity was used to explicitly characterise — rather than assume — the natural variability of old-growth forest composition used as the recovery benchmark.
