# Supplementary material for: Regenerating forests around Taï National Park sustain biodiversity despite constrained floristic recovery
Source: PLoS One. 2026 Oct 21;21(10):e0356900. doi: 10.1371/journal.pone.0356900 (PMC13686277; doi:10.1371/journal.pone.0356900)
Supplement: Supplementary file 3 [file pone.0356900.s006.pdf]

Response to Reviewers **PONE-D-26-18781**
PLOS ONE *September 2nd 2026*

Contents

1 Comment from the Editor **1**

2 Comments from Reviewer 1 **2**
2.1 Trait Terminology Inconsistency (Abstract, L34) 2
2.2 Trait Coverage Discrepancy (Methods L176 vs Discussion L506) 2
2.3 Phrasing and Typographical Corrections 2
2.4 Ecological Discussion Note (L365-367 / Fig. 3) 3

3 Comments from Reviewer 2 **3**
3.1 Detailed responses to annotated comments 4
3.1.1 L54-57: "This sentence is hard to read, rephrase please." 4
3.1.2 L72-74: "Recast this sentence." 4
3.1.3 L81-82: "Tell us more about the endemic and threatened species, list some of them." 5
3.1.4 L89-90: "Where are the few studies?" 5
3.1.5 L113-114: "The drier periods are in which months?" 5
3.1.6 L448: "A central role in what? This phrase is hanging." 5

---

1 Comment from the Editor

> *Dear Dr. Hérault, Thank you for submitting your manuscript to PLOS One. After careful consideration, we feel that it has merit but does not fully meet PLOS One's publication criteria as it currently stands. Therefore, we invite you to submit a revised version of the manuscript that addresses the points raised during the review process. [...] If the reviewer comments include a recommendation to cite specific previously published works, please review and evaluate these publications to determine whether they are relevant and should be cited. There is no requirement to cite these works unless the editor has indicated otherwise. Please review your reference list to ensure that it is complete and correct. If you have cited papers that have been retracted, please include the rationale for doing so in the manuscript text, or remove these references and replace them with relevant current references.*

We thank the Editor for the opportunity to submit a further revision. We are glad that Reviewer 1 considers that all previous comments have been adequately addressed. In the following, our responses to comments are in grey and changes in the text are reported in red. Regarding the Journal Requirements: no citation to a specific previously published work was recommended by the reviewers in this round, so no additional citation was required on that basis (two existing references are, however, added below in direct response to Reviewer 2's comment 2.4). We have reviewed our reference list and confirm that it does not include any retracted articles.

2 Comments from Reviewer 1

This revised manuscript provides a valuable and multidimensional assessment of forest regeneration in and around Taï National Park (Côte d'Ivoire), the largest remaining continuous block of Upper Guinean rainforest. The dataset is substantial and addresses an ecologically critical, underrepresented region. The findings convey a clear and balanced conservation message: while alpha diversity and leaf/regeneration traits rebound relatively quickly, floristic composition and structural attributes (wood density) lag behind, emphasizing that secondary forests complement but cannot substitute for intact old-growth forests.

We are delighted that Reviewer 1 confirms that all comments raised in the previous round have been adequately addressed, and we sincerely thank the reviewer for this positive assessment and for the additional minor points raised below, which we address in turn.

2.1 Trait Terminology Inconsistency (Abstract, L34)

In the Abstract (L-34): the authors mention that "specific leaf area and seed mass recovered rapidly...". However, throughout the rest of the text, methods, tables, and figures, the trait investigated is "Leaf Mass per Area (LMA)". Since SLA is the mathematical inverse of LMA, please replace "specific leaf area" with "leaf mass per area (LMA)" in the Abstract for consistency.

We thank the reviewer for catching this inconsistency, which was indeed a terminology slip in the Abstract only (the trait analysed throughout the manuscript is LMA, never its inverse SLA). We have corrected the Abstract accordingly.
In the Abstract: Regarding functional traits, they displayed contrasting dynamics: leaf mass per area (LMA) and seed mass recovered rapidly along successional gradients [...]

2.2 Trait Coverage Discrepancy (Methods L176 vs Discussion L506)

In Methods (L-176): LMA coverage is stated as "56% of individuals", whereas in the Discussion (Line 506) and the Response to Reviewers, it is reported as "57%". Please verify and harmonize this percentage across the text.

We thank the reviewer for flagging this discrepancy. We have verified the figure against our dataset: the correct value is **56%** of individuals, as already stated in the Methods. The Discussion contained a transcription error (57%) that has now been corrected to match the Methods.
In the Discussion: In addition, LMA values were available for only 56% of individuals, and species lacking a trait record were excluded from this analysis [...]

2.3 Phrasing and Typographical Corrections

Abstract (L-29): Add the missing opening parenthesis for the composition recovery rate: "than floristic composition (\lambda = 0.03)". Abstract (L-30): Correct the subject-verb agreement: change "and these recovery varied" to "and this recovery varied" or "and these recovery rates varied". Figure 3 vs. Table 1: In Fig. 3, the covariate label is displayed as "Animals", while Table 1 and the main text use "Mammals". Harmonizing these labels will improve clarity.

We have tidied the parenthesis spacing around both λ values in the Abstract, and corrected the subject-verb agreement as suggested (we opted for "these recovery rates varied", the reviewer's second proposed wording). Regarding Fig. 3 vs. Table 1: we keep "Animals", and have renamed the "Mammal" covariate to "Animal" in Table 1, in the Results text, and in the Fig. 3 caption, so that the manuscript now consistently uses "Animal" throughout.
In the Abstract: Diversity was restored more rapidly ($\lambda = 0.06$) than floristic composition ($\lambda = 0.03$), and these recovery rates varied according to environmental variables.
In Table 1: Animal – Number of large/medium-sized mammal species detected through indirect signs – 0-3.
In the Results: Animal presence increased compositional recovery but was negatively associated with diversity recovery.
In the Fig. 3 caption: Covariates include animal presence, remnant tree density, soil hydromorphy, island location, human activities, and disturbance intensity.

2.4 Ecological Discussion Note (L365-367 / Fig. 3)

> *Mammal presence had a positive effect on compositional convergence toward old-growth conditions but was negatively associated with alpha diversity recovery. A brief explanatory sentence in the Discussion would be helpful (e.g., efficient zoocoric dispersal accelerating dominance of heavy-seeded mature-forest specialists at the expense of high numbers of generalist pioneer stems).*

We thank the reviewer for this constructive suggestion, and have added an explanatory sentence to the Discussion along the lines proposed, immediately after the paragraph on remnant trees and seed dispersers.
In the Discussion: Animal presence showed contrasting effects on the two attributes, increasing compositional recovery while being negatively associated with diversity recovery (Fig 3); this is consistent with zoochorous dispersal selectively favouring a narrower set of heavy-seeded, shade-tolerant, mature-forest specialists, driving compositional convergence at the expense of the many wind- and self-dispersed generalist pioneers that inflate species diversity in less animal-frequented secondary forests.

3 Comments from Reviewer 2

> *The manuscript titled "Regenerating forests around Taï National Park sustain biodiversity despite constrained floristic recovery" was well designed and the manuscript structure was well structed. However, the introduction would still need some improvement, most especially as it relates to sentence structures and construction. Most of your arguments were hard to comprehend (please see the attached document for my comments). Then, I noticed the earlier reviewers showed their concerns about the model project of 200 years while the datasets used was from a period of 30 years, and from the revised version it appears the authors have not addressed those concerns, please revisit them and reconcile before your next submission. Then, the methodology would also need some improvement, as earlier raised. Also try to give the manuscript to an English editor to help in some of the grammars. Otherwise, the study is good and very well need in West Africa a largely understudied region as far as forest conservation is concern. Well done.*

We thank Reviewer 2 for the positive overall assessment and for taking the time to annotate the manuscript in detail. We address each annotated comment individually in section 3.1 below, most of which concern the clarity of specific sentences in the Introduction; the other general points raised are addressed here.

- **200-year projection.** This exact concern was already raised by Reviewer 1 in the previous (Major Revision) round, and was addressed at that time – this earlier response is unchanged in the current version of the manuscript. Specifically, Fig. 2 already distinguishes, via solid vs. dashed lines, the temporal range robustly informed by our 9–26 year chronosequence (up to ∼40 years since disturbance, shown as solid lines) from the extrapolated portion beyond it (up to 200 years, shown as dashed lines with widening 95% credible intervals). The figure caption states explicitly that the dashed segment "represent[s] the model's extrapolation beyond this range, up to 200 years, and should be interpreted with caution given the increasing uncertainty." Correspondingly, all interpretive claims in the Results (L331–332: "reaching approximately 75% of its asymptotic value within the first 40 years") and Discussion (L444–448, on the possibility of near-stagnant compositional recovery) are based only on the robust, data-informed portion of the trajectories, not on the 200-year extrapolation. We have therefore not altered this part of the manuscript further, since it already implements exactly what was requested, but remain glad to make any additional specific change the reviewer or editor consider useful.
- **Methodology and statistical analysis.** No specific additional methodological concern was identified in the annotations we received (detailed in section 3.1). We note that Reviewer 2 also answered "No" to the closed question on whether the statistical analysis was performed appropriately, but this was not accompanied by any annotation or comment identifying the concern (confirmed by an exhaustive check of all annotations left on the manuscript). Should particular aspects of the statistical framework remain unclear, we would welcome the reviewer's precise pointers so we can address them directly.
- **Data availability.** We also note that Reviewer 2 answered "No" to the closed question on whether the data underlying the findings were fully available, again without an accompanying comment. On checking, we found the likely cause: our Data Availability Statement stated that the data "will be publicly available [...] upon acceptance", i.e. described as available only in the future rather than at the time of review, which does not meet PLOS ONE's policy of immediate public availability. This was an error in the statement's wording, not a reflection of the data's actual status: the full dataset has in fact already been public on Zenodo since submission. We have corrected the Data Availability Statement accordingly.
  - In the text: All original data used in this study are publicly available and can be accessed from Zenodo (https://doi.org/10.5281/zenodo.21505800).
- **Language editing.** Following this and the reviewer's specific comments, we have carried out an additional careful proofreading and rephrasing pass over the Introduction and other flagged passages to improve readability and grammar.

3.1 Detailed responses to annotated comments

3.1.1 L54-57: "This sentence is hard to read, rephrase please."

We have split and simplified this sentence, replacing the vague "does not progress equally across ecological features" with a clearer statement that different biodiversity attributes recover at dif-

ferent paces, and added "whereas" to explicitly contrast the diversity and composition patterns.
In the text: [...] and may contribute meaningfully to biodiversity conservation [10,11]. Recovery is nonetheless complex: different biodiversity attributes do not recover at the same pace. Species diversity, for example, often increases relatively rapidly following agricultural abandonment [12,13], whereas floristic composition may take decades to converge toward old-growth conditions [14,15].

3.1.2 L72-74: "Recast this sentence."

We have split this long sentence into two shorter ones for clarity.
In the text: Old-growth forests are now extremely scarce in West Africa, a region where deforestation rates remain among the highest globally. As a result, conservation increasingly depends on the capacity of secondary forests to sustain biodiversity.

3.1.3 L81-82: "Tell us more about the endemic and threatened species, list some of them."

We have added three examples of flagship endangered species known from Taï National Park, all listed as Endangered on the IUCN Red List, to illustrate this statement concretely.
In the text: As a major stronghold of regional biodiversity, it harbours numerous endemic and threatened species, including the pygmy hippopotamus (*Choeropsis liberiensis*), the western chimpanzee (*Pan troglodytes verus*), and Diana's monkey (*Cercopithecus diana*), all classified as Endangered on the IUCN Red List.

3.1.4 L89-90: "Where are the few studies?"

We have added a citation to two studies that, to our knowledge, are among the few to have jointly examined multiple dimensions of forest recovery (taxonomic and compositional/functional) in West African secondary forests.
In the text: To date, few studies in Upper Guinean forests have jointly quantified taxonomic, functional, compositional, and conservation-relevant recovery using reference-based models [19,70].

3.1.5 L113-114: "The drier periods are in which months?"

We have specified the two drier periods characteristic of the regional bimodal climate.
In the text: [...] two rainy seasons (March to July and September to November) and two relatively drier periods (a short dry season in August and a longer one from December to February).

3.1.6 L448: "A central role in what? This phrase is hanging."

We have completed this sentence, which was indeed left hanging.
In the text: Previous land use played a central role in shaping these recovery trajectories.
