# Supplementary material for: Regenerating forests around Taï National Park sustain biodiversity despite constrained floristic recovery
Source: PLoS One. 2026 Oct 21;21(10):e0356900. doi: 10.1371/journal.pone.0356900 (PMC13686277; doi:10.1371/journal.pone.0356900)
Supplement: Supplementary file 4 [file pone.0356900.s001.docx]

**S1 Appendix. IUCN-listed tree species recorded in Taï National Park (Côte d’Ivoire).**

Species classified as Endangered (EN) or Vulnerable (VU) according to the IUCN Red List. Values of 1 indicate the assigned conservation category.

| **Species** | **Family** | **EN** | **VU** |
| --- | --- | --- | --- |
| *Albizia ferruginea* | Fabaceae | 0 | 1 |
| *Berlinia occidentalis* | Fabaceae | 0 | 1 |
| *Cassia fikifiki* | Fabaceae | 1 | 0 |
| *Entandrophragma candollei* | Meliaceae | 0 | 1 |
| *Entandrophragma cylindricum* | Meliaceae | 0 | 1 |
| *Entandrophragma utile* | Meliaceae | 0 | 1 |
| *Garcinia afzelii* | Clusiaceae | 0 | 1 |
| *Gilbertiodendron splendidum* | Fabaceae | 0 | 1 |
| *Heritiera utilis* | Malvaceae | 0 | 1 |
| *Lophira alata* | Ochnaceae | 0 | 1 |
| *Nauclea diderrichii* | Rubiaceae | 0 | 1 |
| *Placodiscus bancoensis* | Sapindaceae | 0 | 1 |
| *Placodiscus boya* | Sapindaceae | 0 | 1 |
| *Pterygota bequaertii* | Malvaceae | 0 | 1 |
| *Ricinodendron heudelotii* | Euphorbiaceae | 0 | 1 |
| *Terminalia ivorensis* | Combretaceae | 0 | 1 |
