# Supplementary material for: Regenerating forests around Taï National Park sustain biodiversity despite constrained floristic recovery
Source: PLoS One. 2026 Oct 21;21(10):e0356900. doi: 10.1371/journal.pone.0356900 (PMC13686277; doi:10.1371/journal.pone.0356900)
Supplement: Supplementary file 5 [file pone.0356900.s002.docx]

## **Appendix 2: Old-growth indicator species identified using the Dufrêne–Legendre indicator value method.** Indicator species associated with intact old-growth forests, identified using the Dufrêne–Legendre indicator value (IndVal) method. Only species with significant association (P < 0.002) are reported. IndVal values range from 0 (no association) to 1 (perfect specificity and fidelity to old-growth forests).

| **Indicators species** | **Indval** |
| --- | --- |
| *Maesobotrya barteri* | 0.9 |
| *Calpocalyx brevibracteatus* | 0.9 |
| *Berlinia occidentalis* | 0.9 |
| *Drypetes chevalieri* | 0.9 |
| *Diospyros sanza-minika* | 0.8 |
| *Napoleonaea vogelii* | 0.8 |
| *Diospyros mannii* | 0.8 |
| *Leptaulus daphnoides* | 0.8 |
| *Anthonotha sassandraensis* | 0.8 |
| *Massularia acuminata* | 0.8 |
| *Uapaca guineensis* | 0.8 |
| *Discoglypremna caloneura* | 0.8 |
| *Bridelia grandis* | 0.8 |
| *Xylopia aethiopica* | 0.8 |
| *Enantia polycarpa* | 0.8 |
| *Entandrophragma utile* | 0.8 |
| *Funtumia africana* | 0.8 |
| *Hunteria eburnea* | 0.7 |
| *Theobroma grandiflorum* | 0.7 |
| *Calpocalyx aubrevillei* | 0.7 |
| *Microdesmis keayana* | 0.7 |
| *Blighia welwitschii* | 0.7 |
| *Anisophyllea meniaudi* | 0.7 |
| *Anthonotha fragrans* | 0.7 |
| *Trichilia monadelpha* | 0.7 |
| *Tarrietia utilis* | 0.6 |
| *Coula edulis* | 0.6 |
| *Craterispermum caudatum* | 0.6 |
| *Dialium aubrevillei* | 0.6 |
| *Vitex rivularis* | 0.6 |
| *Cola lateritia* | 0.6 |
| *Garcinia afzelii* | 0.6 |
| *Palisota hirsuta* | 0.6 |
| *Desplatsia chrysochlamys* | 0.5 |
| *Cola buntingii* | 0.5 |
| *Synsepalum brevipes* | 0.5 |
| *Heritiera utilis* | 0.5 |
| *Buchholzia coriacea* | 0.4 |
| *Tabernaemontana crassa* | 0.4 |
| *Alchornea floribunda* | 0.4 |
| *Protomegabaria stapfiana* | 0.4 |
| *Placodiscus bancoensis* | 0.3 |
| *Ouratea parviflora* | 0.3 |
