# Supplementary material for: Regenerating forests around Taï National Park sustain biodiversity despite constrained floristic recovery
Source: PLoS One. 2026 Oct 21;21(10):e0356900. doi: 10.1371/journal.pone.0356900 (PMC13686277; doi:10.1371/journal.pone.0356900)
Supplement: Peer review history

# Peer review: Decision Letter 0

**DOI:** 10.1371/journal.pone.0356900.r001

**Authors:** Luisa Diele-Viegas

28 Jun 2026

Dear Dr. Hérault,

Thank you for submitting your manuscript to PLOS ONE. After careful consideration, we feel that it has merit but does not fully meet PLOS ONE’s publication criteria as it currently stands. Therefore, we invite you to submit a revised version of the manuscript that addresses the points raised during the review process.

==============================

Dear authors,

We have received the review of your manuscript. The reviewer commends the immense effort dedicated to your field data collection and acknowledges that your dataset represents a valuable contribution to West African forest ecology, particularly given the integration of taxonomic, functional, and compositional dimensions via a Bayesian framework.

Based on the reviewer’s comments, I have reached a decision of **Major Revision** .

While the manuscript shows significant promise, there are several methodological and interpretive concerns that must be addressed to ensure the rigor of your conclusions. Please pay close attention to the following points when preparing your revised submission:

- **Temporal Extrapolation:**  Your models currently project trajectories up to 200 years based on a 9–26 year chronosequence. This creates a risk of speculative inference. Please restrict the figures and discussion to the effectively observed temporal horizon (approximately 30 years) to maintain the robustness of your findings.
- **Statistical Uncertainty:**  The 90% credible interval for the recovery rate (lambda) of floristic composition includes 0.00. Please provide a more transparent and cautious discussion regarding the possibility of stagnation in recovery, rather than presenting a categorical claim of convergence.
- **Methodological Transparency & Limitations:**
  **Trait Imputation:**  Given that 43% of the Leaf Mass per Area (LMA) data was imputed using global averages, please include a more robust discussion/caution regarding the interpretation of "rapid convergence." Clarify whether this reflects in situ adaptation or an artifact of the imputation.
- **Voucher Specimens:**  As vouchers were not deposited, please provide detailed information on the specific regional floras or keys utilized for species identification to ensure the reproducibility of your inventory.

==============================

Please submit your revised manuscript by Aug 12 2026 11:59PM. If you will need more time than this to complete your revisions, please reply to this message or contact the journal office at plosone@plos.org. When you're ready to submit your revision, log on to https://www.editorialmanager.com/pone/ and select the 'Submissions Needing Revision' folder to locate your manuscript file.

- A letter that responds to each point raised by the academic editor and reviewer(s). You should upload this letter as a separate file labeled 'Response to Reviewers'.
- A marked-up copy of your manuscript that highlights changes made to the original version. You should upload this as a separate file labeled 'Revised Manuscript with Track Changes'.
- An unmarked version of your revised paper without tracked changes. You should upload this as a separate file labeled 'Manuscript'.

If you would like to make changes to your financial disclosure, please include your updated statement in your cover letter. Guidelines for resubmitting your figure files are available below the reviewer comments at the end of this letter.

If applicable, we recommend that you deposit your laboratory protocols in protocols.io to enhance the reproducibility of your results. Protocols.io assigns your protocol its own identifier (DOI) so that it can be cited independently in the future. For instructions see: https://journals.plos.org/plosone/s/submission-guidelines#loc-laboratory-protocols. Additionally, PLOS ONE offers an option for publishing peer-reviewed Lab Protocol articles, which describe protocols hosted on protocols.io. Read more information on sharing protocols at https://plos.org/protocols?utm_medium=editorial-email&utm_source=authorletters&utm_campaign=protocols.

As the corresponding author, your ORCID iD is verified in the submission system and will appear in the published article. PLOS supports the use of ORCID, and we encourage all coauthors to register for an ORCID iD and use it as well. Please encourage your coauthors to verify their ORCID iD within the submission system before final acceptance, as unverified ORCID iDs will not appear in the published article. *Only*  the individual author can complete the verification step; PLOS staff *cannot*  verify ORCID iDs on behalf of authors.

We look forward to receiving your revised manuscript.

Kind regards,

Luisa Maria Diele-Viegas, Ph. D.

Academic Editor

PLOS One

Journal Requirements:

When submitting your revision, we need you to address these additional requirements.

1. Please ensure that your manuscript meets PLOS ONE's style requirements, including those for file naming. The PLOS ONE style templates can be found at

https://journals.plos.org/plosone/s/file?id=wjVg/PLOSOne_formatting_sample_main_body.pdf and https://journals.plos.org/plosone/s/file?id=ba62/PLOSOne_formatting_sample_title_authors_affiliations.pdf

2. Please include a complete copy of PLOS’ questionnaire on inclusivity in global research in your revised manuscript. Our policy for research in this area aims to improve transparency in the reporting of research performed outside of researchers’ own country or community. The policy applies to researchers who have travelled to a different country to conduct research, research with Indigenous populations or their lands, and research on cultural artefacts. The questionnaire can also be requested at the journal’s discretion for any other submissions, even if these conditions are not met.  Please find more information on the policy and a link to download a blank copy of the questionnaire here: https://journals.plos.org/plosone/s/best-practices-in-research-reporting. Please upload a completed version of your questionnaire as Supporting Information when you resubmit your manuscript.

3. Please note that PLOS One has specific guidelines on code sharing for submissions in which author-generated code underpins the findings in the manuscript. In these cases, we expect all author-generated code to be made available without restrictions upon publication of the work. Please review our guidelines at https://journals.plos.org/plosone/s/materials-and-software-sharing#loc-sharing-code and ensure that your code is shared in a way that follows best practice and facilitates reproducibility and reuse.

4. Please provide a complete Data Availability Statement in the submission form, ensuring you include all necessary access information or a reason for why you are unable to make your data freely accessible. If your research concerns only data provided within your submission, please write "All data are in the manuscript and/or supporting information files" as your Data Availability Statement.

5. Please amend your list of authors on the manuscript to ensure that each author is linked to an affiliation. Authors’ affiliations should reflect the institution where the work was done (if authors moved subsequently, you can also list the new affiliation stating “current affiliation:….” as necessary).

6. We note that Figure 1 in your submission contain [map/satellite] images which may be copyrighted. All PLOS content is published under the Creative Commons Attribution License (CC BY 4.0), which means that the manuscript, images, and Supporting Information files will be freely available online, and any third party is permitted to access, download, copy, distribute, and use these materials in any way, even commercially, with proper attribution. For these reasons, we cannot publish previously copyrighted maps or satellite images created using proprietary data, such as Google software (Google Maps, Street View, and Earth). For more information, see our copyright guidelines: http://journals.plos.org/plosone/s/licenses-and-copyright.

We require you to either (1) present written permission from the copyright holder to publish these figures specifically under the CC BY 4.0 license, or (2) remove the figures from your submission:

a. You may seek permission from the original copyright holder of Figure 1 to publish the content specifically under the CC BY 4.0 license.

We recommend that you contact the original copyright holder with the Content Permission Form (http://journals.plos.org/plosone/s/file?id=7c09/content-permission-form.pdf) and the following text:

“I request permission for the open-access journal PLOS ONE to publish XXX under the Creative Commons Attribution License (CCAL) CC BY 4.0 (http://creativecommons.org/licenses/by/4.0/). Please be aware that this license allows unrestricted use and distribution, even commercially, by third parties. Please reply and provide explicit written permission to publish XXX under a CC BY license and complete the attached form.”

Please upload the completed Content Permission Form or other proof of granted permissions as an "Other" file with your submission.

In the figure caption of the copyrighted figure, please include the following text: “Reprinted from [ref] under a CC BY license, with permission from [name of publisher], original copyright [original copyright year].”

b. If you are unable to obtain permission from the original copyright holder to publish these figures under the CC BY 4.0 license or if the copyright holder’s requirements are incompatible with the CC BY 4.0 license, please either i) remove the figure or ii) supply a replacement figure that complies with the CC BY 4.0 license. Please check copyright information on all replacement figures and update the figure caption with source information. If applicable, please specify in the figure caption text when a figure is similar but not identical to the original image and is therefore for illustrative purposes only.

The following resources for replacing copyrighted map figures may be helpful:

USGS National Map Viewer (public domain): http://viewer.nationalmap.gov/viewer/

The Gateway to Astronaut Photography of Earth (public domain): http://eol.jsc.nasa.gov/sseop/clickmap/

Maps at the CIA (public domain): https://www.cia.gov/library/publications/the-world-factbook/index.html and https://www.cia.gov/library/publications/cia-maps-publications/index.html

NASA Earth Observatory (public domain): http://earthobservatory.nasa.gov/

Landsat: http://landsat.visibleearth.nasa.gov/

USGS EROS (Earth Resources Observatory and Science (EROS) Center) (public domain): http://eros.usgs.gov/#

Natural Earth (public domain): http://www.naturalearthdata.com/

7. If the reviewer comments include a recommendation to cite specific previously published works, please review and evaluate these publications to determine whether they are relevant and should be cited. There is no requirement to cite these works unless the editor has indicated otherwise.

[Note: HTML markup is below. Please do not edit.]

Reviewers' comments:

Reviewer's Responses to Questions

**Comments to the Author**

1. Is the manuscript technically sound, and do the data support the conclusions?

Reviewer #1: Yes

**********

2. Has the statistical analysis been performed appropriately and rigorously?

Reviewer #1: No

**********

3. Have the authors made all data underlying the findings in their manuscript fully available?

The PLOS Data policy

Reviewer #1: Yes

**********

4. Is the manuscript presented in an intelligible fashion and written in standard English?

Reviewer #1: Yes

**********

Reviewer #1: Dear Authors,

I would like to congratulate you on your work. The immense effort dedicated to field data collection in the Taï National Park—a region of inestimable ecological importance is clearly evident. Using a Bayesian framework to integrate taxonomic, functional, and compositional dimensions is a valuable and welcome contribution to West African forest ecology.

I am a strong believer in collaborative science and see great potential in this manuscript. To ensure the work meets the technical rigor required, I suggest a few adjustments that will strengthen the foundation of your conclusions:

L128-134: Temporal Scale Adjustment: The study presents a solid dataset from a 9 to 26-year chronosequence. However, the models project trajectories up to 200 years. I kindly suggest that the figures and discussions be restricted to the effectively observed temporal horizon (approximately 30 years). Such long-term projections can introduce uncertainties that may obscure the real, robust results you have already obtained.

L328-329: Uncertainty in Compositional Recovery: I noted that the recovery rate (ʎ) for floristic composition has a 90% credible interval starting at 0.00. This suggests that, statistically, recovery might be in a state of stagnation during the sampled period. A more transparent discussion of this uncertainty would help the reader grasp the complexity of local forest succession.

L111-113: Representativeness of Reference Sites: The forest's "target" state is defined by 15 primary forest plots. Given that the park covers over 5,000 km², it would be insightful to briefly comment on whether these plots adequately capture the natural variability of such a vast ecosystem.

L165-167: Functional Traits and Imputation: The 57% coverage for the LMA (leaf mass per area) trait warrants some caution. Since TRY database values and taxonomic averages were used to fill gaps, the rapid convergence observed in LMA might reflect species turnover rather than in situ functional adaptation.

L147-148: Taxonomic Documentation: Identifying 174 species is an impressive feat. As voucher specimens were not deposited in herbaria, it would be valuable to provide more detail on the specific regional floras or keys used to ensure the reproducibility of the inventory.

I hope you see these points as a supportive means to further elevate the quality of your excellent research. I look forward to seeing the revised version

**********

what does this mean?). If published, this will include your full peer review and any attached files.

If you choose “no”, your identity will remain anonymous but your review may still be made public.

**Do you want your identity to be public for this peer review?**  For information about this choice, including consent withdrawal, please see our Privacy Policy

Reviewer #1: No

**********

[NOTE: If reviewer comments were submitted as an attachment file, they will be attached to this email and accessible via the submission site. Please log into your account, locate the manuscript record, and check for the action link "View Attachments". If this link does not appear, there are no attachment files.]

To ensure your figures meet our technical requirements, please review our figure guidelines: https://journals.plos.org/plosone/s/figures

You may also use PLOS’s free figure tool, NAAS, to help you prepare publication quality figures: https://journals.plos.org/plosone/s/figures#loc-tools-for-figure-preparation.

NAAS will assess whether your figures meet our technical requirements by comparing each figure against our figure specifications.

# Author comment: Author response to Decision Letter 1

**DOI:** 10.1371/journal.pone.0356900.r002

3 Aug 2026

See the cover letter in PDF for a detailed response

Attachment Submitted filename: PlosOne_Response_Reviewers.pdf

# Peer review: Decision Letter 1

**DOI:** 10.1371/journal.pone.0356900.r003

**Authors:** Luisa Diele-Viegas

26 Aug 2026

Dear Dr. Hérault,

Thank you for submitting your manuscript to PLOS One. After careful consideration, we feel that it has merit but does not fully meet PLOS One’s publication criteria as it currently stands. Therefore, we invite you to submit a revised version of the manuscript that addresses the points raised during the review process.

Please submit your revised manuscript by Oct 10 2026 11:59PM. If you will need more time than this to complete your revisions, please reply to this message or contact the journal office at plosone@plos.org. When you're ready to submit your revision, log on to https://www.editorialmanager.com/pone/ and select the 'Submissions Needing Revision' folder to locate your manuscript file.

- A letter that responds to each point raised by the academic editor and reviewer(s). You should upload this letter as a separate file labeled 'Response to Reviewers'.
- A marked-up copy of your manuscript that highlights changes made to the original version. You should upload this as a separate file labeled 'Revised Manuscript with Track Changes'.
- An unmarked version of your revised paper without tracked changes. You should upload this as a separate file labeled 'Manuscript'.

If you would like to make changes to your financial disclosure, please include your updated statement in your cover letter. Guidelines for resubmitting your figure files are available below the reviewer comments at the end of this letter.

If applicable, we recommend that you deposit your laboratory protocols in protocols.io to enhance the reproducibility of your results. Protocols.io assigns your protocol its own identifier (DOI) so that it can be cited independently in the future. For instructions see: https://journals.plos.org/plosone/s/submission-guidelines#loc-laboratory-protocols. Additionally, PLOS One offers an option for publishing peer-reviewed Lab Protocol articles, which describe protocols hosted on protocols.io. Read more information on sharing protocols at https://plos.org/protocols?utm_medium=editorial-email&utm_source=authorletters&utm_campaign=protocols.

As the corresponding author, your ORCID iD is verified in the submission system and will appear in the published article. PLOS supports the use of ORCID, and we encourage all coauthors to register for an ORCID iD and use it as well. Please encourage your coauthors to verify their ORCID iD within the submission system before final acceptance, as unverified ORCID iDs will not appear in the published article. *Only*  the individual author can complete the verification step; PLOS staff *cannot*  verify ORCID iDs on behalf of authors.

We look forward to receiving your revised manuscript.

Kind regards,

Luisa Maria Diele-Viegas, Ph. D.

Academic Editor

PLOS One

Journal Requirements:

If the reviewer comments include a recommendation to cite specific previously published works, please review and evaluate these publications to determine whether they are relevant and should be cited. There is no requirement to cite these works unless the editor has indicated otherwise.

Please review your reference list to ensure that it is complete and correct. If you have cited papers that have been retracted, please include the rationale for doing so in the manuscript text, or remove these references and replace them with relevant current references. Any changes to the reference list should be mentioned in the rebuttal letter that accompanies your revised manuscript. If you need to cite a retracted article, indicate the article’s retracted status in the References list and also include a citation and full reference for the retraction notice.

[Note: HTML markup is below. Please do not edit.]

Reviewers' comments:

Reviewer's Responses to Questions

**Comments to the Author**

Reviewer #1: All comments have been addressed

Reviewer #2: (No Response)

**********

2. Is the manuscript technically sound, and do the data support the conclusions?

Reviewer #1: Yes

Reviewer #2: Yes

**********

3. Has the statistical analysis been performed appropriately and rigorously?

Reviewer #1: Yes

Reviewer #2: No

**********

4. Have the authors made all data underlying the findings in their manuscript fully available?

The PLOS Data policy

Reviewer #1: Yes

Reviewer #2: No

**********

5. Is the manuscript presented in an intelligible fashion and written in standard English?

Reviewer #1: Yes

Reviewer #2: Yes

**********

Reviewer #1: Reviewer Report – Manuscript PONE-D-26-18781

Title: Regenerating forests around Taï National Park sustain biodiversity despite constrained floristic recovery

1. General Overview and Strengths:

This revised manuscript provides a valuable and multidimensional assessment of forest regeneration in and around Taï National Park (Côte d'Ivoire), the largest remaining continuous block of Upper Guinean rainforest.

The dataset is substantial and addresses an ecologically critical, underrepresented region. The findings convey a clear and balanced conservation message: while alpha diversity and leaf/regeneration traits rebound relatively quickly, floristic composition and structural attributes (wood density) lag behind, emphasizing that secondary forests complement but cannot substitute for intact old-growth forests.

2. Specific Minor Points and Corrections Required:

a) Trait Terminology Inconsistency:

- In the Abstract (L-34):

the authors mention that "specific leaf area and seed mass recovered rapidly...". However, throughout the rest of the text, methods, tables, and figures, the trait investigated is "Leaf Mass per Area (LMA)". Since SLA is the mathematical inverse of LMA, please replace "specific leaf area" with "leaf mass per area (LMA)" in the Abstract for consistency.

b) Trait Coverage Discrepancy:

- In Methods (L- 176):

LMA coverage is stated as "56% of individuals", whereas in the Discussion (Line 506) and the Response to Reviewers, it is reported as "57%". Please verify and harmonize this percentage across the text.

d) Phrasing and Typographical Corrections:

- Abstract (L- 29):

Add the missing opening parenthesis for the composition recovery rate: "than floristic composition (\lambda = 0.03)".

- Abstract (L- 30): Correct the subject-verb agreement: change "and these recovery varied" to "and this recovery varied" or "and these recovery rates varied".

- Figure 3 vs. Table 1: In Fig. 3, the covariate label is displayed as "Animals", while Table 1 and the main text use "Mammals". Harmonizing these labels will improve clarity.

e) Ecological Discussion Note:

- L 365–367 / Fig. 3: Mammal presence had a positive effect on compositional convergence toward old-growth conditions but was negatively associated with alpha diversity recovery. A brief explanatory sentence in the Discussion would be helpful (e.g., efficient zoocoric dispersal accelerating dominance of heavy-seeded mature-forest specialists at the expense of high numbers of generalist pioneer stems).

Reviewer #2: The manuscript titled “Regenerating forests around Taï National Park sustain biodiversity despite constrained floristic recovery” was well designed and the manuscript structure was well structed. However, the introduction would still need some improvement, most especially as it relates to sentence structures and construction. Most of your arguments were hard to comprehend (please see the attached document for my comments). Then, I noticed the earlier reviewers showed their concerns about the model project of 200 years while the datasets used was from a period of 30 years, and from the revised version it appears the authors have not addressed those concerns, please revisit them and reconcile before your next submission. Then, the methodology would also need some improvement, as earlier raised. Also try to give the manuscript to an English editor to help in some of the grammars. Otherwise, the study is good and very well need in West Africa a largely understudied region as far as forest conservation is concern. Well done.

**********

what does this mean?). If published, this will include your full peer review and any attached files.

If you choose “no”, your identity will remain anonymous but your review may still be made public.

**Do you want your identity to be public for this peer review?**  For information about this choice, including consent withdrawal, please see our Privacy Policy

Reviewer #1: **Yes:**  ANDRE LUIZ SOARES NUNES

Reviewer #2: No

**********

[NOTE: If reviewer comments were submitted as an attachment file, they will be attached to this email and accessible via the submission site. Please log into your account, locate the manuscript record, and check for the action link "View Attachments". If this link does not appear, there are no attachment files.]

To ensure your figures meet our technical requirements, please review our figure guidelines: https://journals.plos.org/plosone/s/figures

You may also use PLOS’s free figure tool, NAAS, to help you prepare publication quality figures: https://journals.plos.org/plosone/s/figures#loc-tools-for-figure-preparation.

NAAS will assess whether your figures meet our technical requirements by comparing each figure against our figure specifications.

Attachment Submitted filename: PONE-D-26-18781_R1_reviewer-reviewed.pdf

# Author comment: Author response to Decision Letter 2

**DOI:** 10.1371/journal.pone.0356900.r004

4 Sep 2026

Please see the attached PDF file with detailed responses

Attachment Submitted filename: PONE-D-26-18781_Response_to_Reviewers_2.pdf

# Editor report: Decision Letter 2

**DOI:** 10.1371/journal.pone.0356900.r005

**Authors:** Luisa Diele-Viegas

17 Sep 2026

Regenerating forests around Taï National Park sustain biodiversity despite constrained floristic recovery

PONE-D-26-18781R2

Dear Dr. Hérault,

We’re pleased to inform you that your manuscript has been judged scientifically suitable for publication and will be formally accepted for publication once it meets all outstanding technical requirements.

Within one week, you’ll receive an e-mail detailing the required amendments. When these have been addressed, you’ll receive a formal acceptance letter and your manuscript will be scheduled for publication.

An invoice will be generated when your article is formally accepted. Please note, if your institution has a publishing partnership with PLOS and your article meets the relevant criteria, all or part of your publication costs will be covered. Please make sure your user information is up-to-date by logging into Editorial Manager at Editorial Manager® and clicking the ‘Update My Information' link at the top of the page. For questions related to billing, please contact billing support.

If your institution or institutions have a press office, please notify them about your upcoming paper to help maximize its impact. If they’ll be preparing press materials, please inform our press team as soon as possible -- no later than 48 hours after receiving the formal acceptance. Your manuscript will remain under strict press embargo until 2 pm Eastern Time on the date of publication. For more information, please contact onepress@plos.org.

Kind regards,

Luisa Maria Diele-Viegas, Ph. D.

Academic Editor

PLOS One

Additional Editor Comments (optional):

Reviewers' comments:

# Editor report: Acceptance letter

**DOI:** 10.1371/journal.pone.0356900.r006

**Authors:** Luisa Diele-Viegas

PONE-D-26-18781R2

PLOS One

Dear Dr. Hérault,

I'm pleased to inform you that your manuscript has been deemed suitable for publication in PLOS One. Congratulations! Your manuscript is now being handed over to our production team.

At this stage, our production department will prepare your paper for publication. This includes ensuring the following:

* All references, tables, and figures are properly cited

* All relevant supporting information is included in the manuscript submission,

* There are no issues that prevent the paper from being properly typeset

You will receive further instructions from the production team, including instructions on how to review your proof when it is ready. Please keep in mind that we are working through a large volume of accepted articles, so please give us a few days to review your paper and let you know the next and final steps.

Lastly, if your institution or institutions have a press office, please let them know about your upcoming paper now to help maximize its impact. If they'll be preparing press materials, please inform our press team within the next 48 hours. Your manuscript will remain under strict press embargo until 2 pm Eastern Time on the date of publication. For more information, please contact onepress@plos.org.

You will receive an invoice from PLOS for your publication fee after your manuscript has reached the completed accept phase. If you receive an email requesting payment before acceptance or for any other service, this may be a phishing scheme. Learn how to identify phishing emails and protect your accounts at https://explore.plos.org/phishing.

If we can help with anything else, please email us at customercare@plos.org.

Thank you for submitting your work to PLOS One and supporting open access.

Kind regards,

PLOS One Editorial Office Staff

on behalf of

Dr. Luisa Maria Diele-Viegas

Academic Editor

PLOS One
